# Allosteric modulation of the solute carrier transporter SLC39A8 potentiates manganese and cadmium uptake

**DOI:** 10.1172/JCI191096

**Published:** 2025-11-03

**Authors:** Kelly L. Damm-Ganamet, Clara Moon, Alan D. Wickenden, Mark Tichenor, Yunhui Ge, Eduardo V. Mercado-Marin, Brian Chiou, Ayla Manughian-Peter, Taraneh Mirzadegan, Jennifer D. Venable, Ramnik J. Xavier, Jennifer E. Towne, Daniel B. Graham, Jacqueline Perrigoue

**Affiliations:** 1 Therapeutics Discovery and; 2 Discovery Immunology, Johnson & Johnson, San Diego, California, USA.; 3 Broad Institute of MIT and Harvard, Cambridge, Massachusetts, USA.; 4 Center for Computational and Integrative Biology, Department of Molecular Biology, Massachusetts General Hospital, Harvard Medical School, Boston, Massachusetts, USA.; 5 Discovery Immunology, Johnson & Johnson, Spring House, Pennsylvania, USA.

**Keywords:** Genetics, Therapeutics, Genetic diseases, Inflammatory bowel disease, Transport

## Abstract

Solute carrier (SLC) transporters govern the selective transport of diverse molecules across cell membranes, controlling fundamental metabolic and cellular processes. Despite genetic evidence implicating SLC transporters in a variety of human diseases, this family of proteins represents an underexplored target class for therapeutic drug discovery. Here, we discovered a selective potentiator of SLC39A8, a metal transporter associated with inflammatory bowel disease, schizophrenia, and cardiovascular and metabolic disorders. We conducted a drug repurposing screen, identifying efavirenz as a potentiator of manganese and cadmium uptake by SLC39A8 and subsequently generated structure-activity relationships to guide design of analogs. Computational pocket identification methodology and molecular dynamic simulations revealed a ligandable, cryptic pocket that, together with functional mutagenesis, indicated direct target engagement and allosteric modulation. Our findings demonstrate how the combination of experimental data and computational tools represents a powerful synergy that can enhance scientific outcomes. This integrated approach allowed for iterative feedback where insights from experiments informed the model refinements and computational predictions guided future experimental designs. Furthermore, our data established that SLC39A8 transporter activity can be increased pharmacologically, potentially opening avenues for SLC transporter drug discovery.

## Abstract 2

The significance of this work lies in its exploration of SLC transporters as viable therapeutic targets, which has implications for several diseases. By identifying efavirenz as a selective potentiator of SLC39A8, this research not only showcases the potential of drug repurposing as an efficient strategy in discovery but also provides valuable insights into the role of this transporter in regulating essential metal ion transport. Furthermore, the combination of computational tools and experimental findings illustrates how comprehensive insights can be achieved, leading to the identification of an allosteric, cryptic pocket and opening new pathways for the development of highly selective drugs that target this region. Ultimately, this research contributes to expanding the therapeutic landscape for addressing a spectrum of diseases linked to SLC transporters.

## Introduction

Solute carrier (SLC) transporters are the second largest family of membrane proteins in the human genome, encompassing 52 gene families comprising over 400 distinct human transporter genes. SLC transporters are the molecular target of several approved inhibitor drugs, such as selective serotonin reuptake inhibitors for depression and sodium/glucose cotransporter 2 inhibitors for diabetes. However, only limited examples of transporter potentiators have been described (1–3). Since reduced function of SLC transporters is more commonly linked to disease, identification of positive modulators could dramatically increase the therapeutic potential of this target class.

SLC39A8, also known as ZIP8, is a metal transporter that plays an essential role in zinc (Zn) and manganese (Mn) homeostasis. Rare mutations in *SLC39A8* cause severe congenital disorders of glycosylation (CDG type IIn) or mitochondrial dysfunction (Leigh syndrome) (4). Hypomorphic and loss-of-function alleles of *SLC39A8* cause a severe diminution of systemic Mn levels in humans (4–6) and mice (7, 8) that in turn disrupts developmental processes and the homeostatic function of several organ systems. The widespread utilization of Mn across biological processes stems from its function as a key cofactor for a variety of enzymes, including glycosyltransferases, lyases, hydrolases, and metalloenzymes (9–12), and is consistent with the pleiotropic effects of *SLC39A8* variants.

GWASs have linked rs13107325, a single nucleotide polymorphism (SNP) in *SLC39A8*, with diverse clinical phenotypes, including risk of inflammatory bowel disease (IBD), schizophrenia, and Parkinson’s disease, as well as cardiovascular and metabolic phenotypes. This pleiotropic missense variant of *SLC39A8*, encoding A391T, exhibits a partial loss of function associated with a reduction in systemic and tissue Mn levels in humans (13, 14) and mouse models (15, 16). Most individuals harboring the A391T variant are healthy, suggesting epistatic or extrinsic environmental triggers may be required to elicit a disease phenotype. The A391T variant may therefore represent a common underlying genetic predisposition associated with a distinct spectrum of triggers. For example, oxidative stress is counteracted by Mn-dependent SOD. Neurons are particularly sensitive to oxidative stress, and *SLC39A8* has been implicated in mitochondrial dysfunction due to defective SOD (4, 17, 18). In the context of IBD, the *SLC39A8* A391T variant has been implicated in susceptibility to intestinal inflammation due to impaired barrier defense. *SLC39A8* A391T knockin mice exhibit reduced systemic Mn levels that can be exacerbated or mimicked by restricting Mn intake in the diet (15). Consequently, glycoprotein barrier structures, including the glycocalyx and colonic mucus layer, are compromised, rendering A391T mice highly susceptible to colonic inflammation induced by epithelial injury (15, 16). These findings are consistent with the strict Mn dependence of glycosyltransferase enzymes that help assemble these barrier structures (19–21). Together, the pleiotropic effects of *SLC39A8* variants in human disease and the mechanistic link to disruption of Mn homeostasis suggest that potentiators of Mn uptake or increased expression of this transporter may have therapeutic benefit across a variety of indications.

SLC39A8 contains a highly structurally conserved transmembrane domain (TMD) with 8 transmembrane helices (TMs) and a large, N-terminal extracellular domain. There are no crystal structures of human SLC39A8, but structures of the TMD from the related prokaryotic Zn transporter ZIP from *Bordetella bronchiseptica* (BbZIP) have been solved (22–26) in addition to a cryo–electron microscopy structure (27). The structures demonstrate a single inward-facing conformation (IFC) as an apo or metal-bound state (with interacting Zn^2+^ and/or Cd^2+^). An ion-binding region is observed where the metal is coordinated by TM4 and TM5, and a substantial cavity between the 2 bundles provides access to this region from the intracellular side. The proposed cavity entrance is occluded at the extracellular side by highly conserved hydrophobic residues. A recent study utilized AlphaFold2 homology models to generate multiple conformations of representative SLC39 transporters and hypothesized that, like other SLC transporters, they function with an “elevator-type” mechanism (23), utilizing distinct outward and inward conformations (28). This mechanism allows substrates and ions to pass across the membrane by alternating access to the extracellular (outward) and intracellular (inward) spaces. Mutagenesis and uptake assays support the elevator-type transport mechanism hypothesized from computational modeling and demonstrate the regulated substrate translocation and the role of specific domains in Zn transport (23, 24, 29).

While SLCs are considerably underexplored as small-molecule drug targets, there are SLC classes targeted by approved drugs, establishing precedence and demonstrating the druggability of the family (30). The first reported inhibitor of SLC39A8 was found from a screen of a GDB-17 fragment library by Pujol-Gimenez at al. and confirmed using a cell-based assay (IC_50_ = 15.5 ± 1.2 mM) (31). However, there are currently no small-molecule SLC39A8 potentiators reported in the literature. Here, we demonstrate that disease-linked mutations lead to the partial loss of both SLC39A8 expression and function. Furthermore, we identify a small-molecule potentiator that increases the function of SLC39A8 and disease-linked variants by a putative allosteric mechanism that involves residues located at the interface of TMDs 1, 4, and 5. To our knowledge, this is the first example of a pharmacologic potentiator of this SLC transporter class and expands therapeutic entry points for treating diseases linked to defects in Mn homeostasis.

## Results

### Disease-associated variants decrease expression and function of SLC39A8.

**Figure 1:**
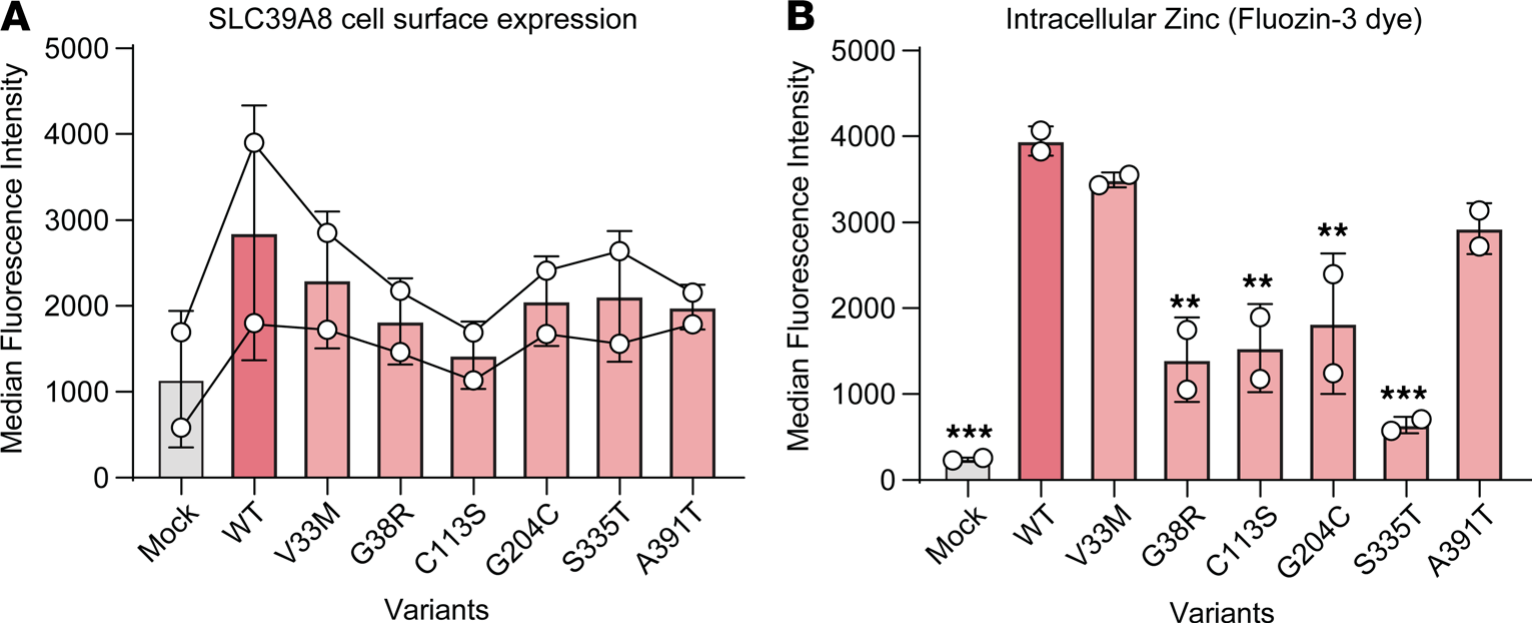
Disease-associated variants decrease expression and function of SLC39A8. HEK293T cells were transiently transfected with FLAG-tagged WT SLC39A8 or the indicated disease-associated mutant. (**A**) Cell surface expression of the SLC39A8 proteins was detected by flow cytometry using an anti-FLAG–Alexa 647 antibody. The median fluorescence intensity is shown for the SLC39A8-expressing population of cells (*n* = 2 experiments; lines connect data points from each experiment). (**B**) Intracellular Zn was measured using FluoZin-3 cell permeant Zn indicator dye. The median fluorescence intensity is shown for the SLC39A8-expressing cell population (*n* = 2 experiments). One-way ANOVA, *P* = 0.0001. Dunnett’s multiple-comparison test; asterisks denote significance compared with WT (**P < 0.005, ***P < 0.0005). Data in **A** and **B** are presented as mean ± SEM.

Several variants in *SLC39A8* have been associated with rare monogenic diseases, such as CDG type IIn and Leigh syndrome (4–6), as well as more common polygenic diseases including IBD, cardiometabolic disease, schizophrenia, and Parkinson’s disease, among others (32–34). To determine the functional impact of these variants, WT SLC39A8 and 6 different FLAG-tagged variants were transiently transfected into HEK293T cells and analyzed for SLC39A8 gene expression, cell surface protein expression, and intracellular Zn uptake by flow cytometry. While the parental HEK293T cells showed negligible *SLC39A8* mRNA expression, transfection with WT or CDG variant constructs led to a robust upregulation of *SLC39A8* gene expression to similar levels (Supplemental Figure 1; supplemental material available online with this article; https://doi.org/10.1172/JCI191096DS1). All variants showed varying levels of decreased cell surface SLC39A8 protein expression compared with WT *SLC39A8* (Figure 1A). This largely correlated with a decrease in intracellular Zn uptake, measured by the FluoZin-3 Zn-selective indicator dye (Figure 1B). Notably, S335T was associated with a moderately reduced expression of *SLC39A8* and a disproportionate decrease in Zn uptake, suggesting an additional level of functional impairment beyond that conferred by expression. Overall, the largest effect size with respect to diminished intracellular Zn levels was associated with variants from CDG and Leigh syndrome. The exception to this observation was V33M, which was reported in a compound heterozygous individual that also carried G204C and S335T (17). The more common pleiotropic variant A391T exhibited a partial reduction in expression and commensurate reduction in intracellular Zn, which is consistent with the incomplete penetrance of phenotypes associated with this variant and the notion that the variant is not pathogenic on its own but requires an environmental or epistatic trigger. Together, these data show that various disease-associated mutations in *SLC39A8* lead to reduced SLC39A8 protein expression on the cell surface, resulting in impaired metal ion uptake and suggesting a therapeutic role for SLC39A8 potentiation in disease.

### Small-molecule screen identifies a potentiator of SLC39A8.

**Figure 2:**
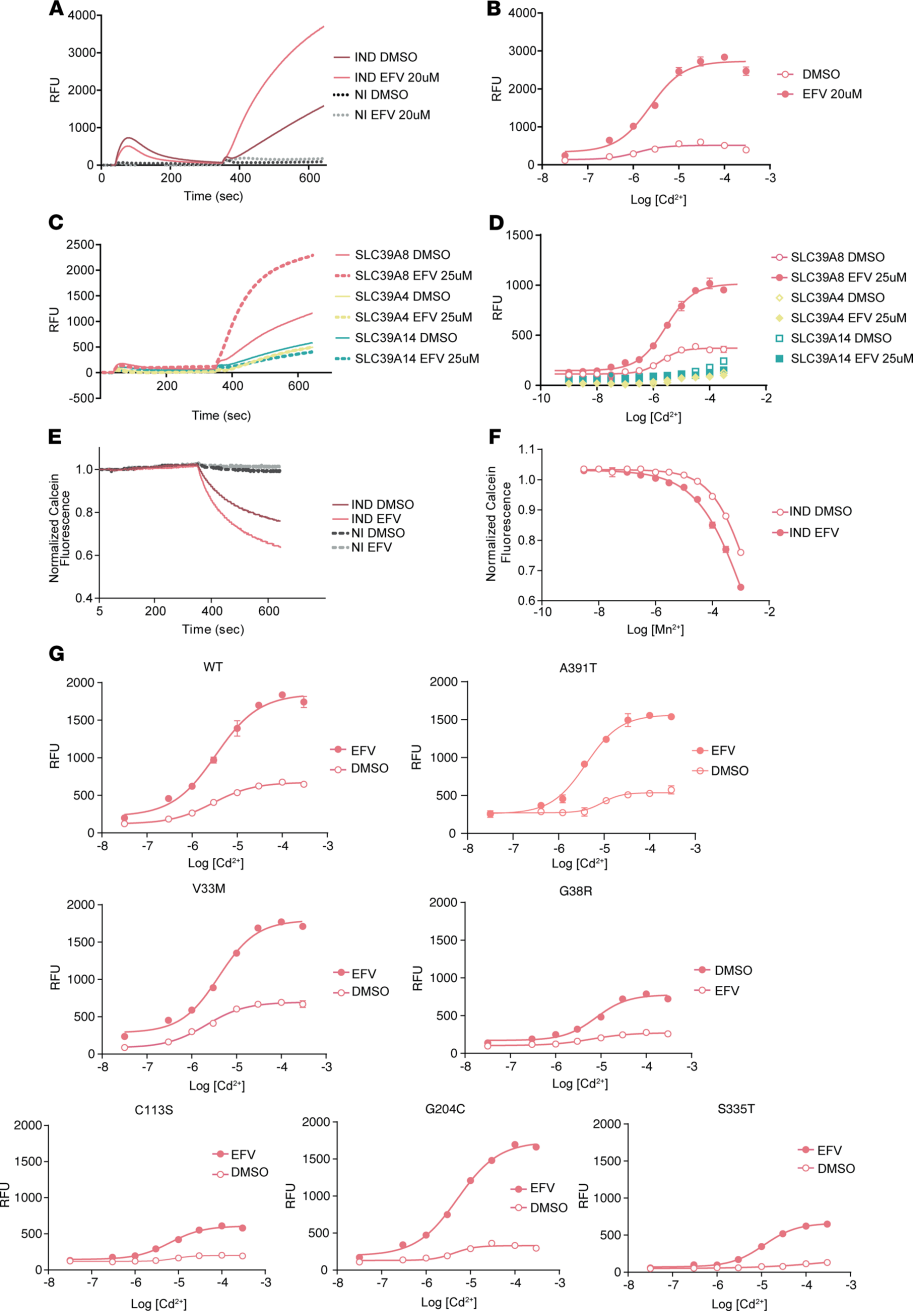
EFV potentiates SLC39A8 activity. EFV was identified from a screen of the Prestwick library as an SLC39A8 potentiator. (**A** and **B**) EFV potentiates Cd uptake, as measured by Calcium 6 fluorescence. EFV markedly increases apparent V*_max_* and modestly decreases Cd. (**C** and **D**) EFV has no effect on Cd uptake in cells expressing SLC39A4 or SLC39A14. (**E** and **F**) EFV potentiates Mn uptake by SLC39A8, as measured by calcein quenching. (**G**) EFV rescues Cd uptake in cells expressing disease-associated SLC39A8 variants. Data presented in **B**, **D**, **F**, and **G** are presented as mean ± SEM. Replicates: **A**, *n* = 3–4; **B**, *n* = 2; and **C**–**F**, *n* = 3. Concentrations used: **A**, 30 μM Cd; **C**, 100 μM Cd; and **E**, 1 mM Mn. IND, induced; NI, noninduced; RFU, relative fluorescence units.

We developed a screening system to identify chemical matter able to potentiate the activity of SLC39A8 that might serve as chemical starting points to develop compounds that restore Mn homeostasis in cells. We first engineered HEK293-T-REx cells for inducible expression of SLC39A8 and developed a metal uptake assay strictly dependent on SLC39A8 induction. We selected cadmium (Cd) as a surrogate for Mn uptake because this metal is a substrate for SLC39A8 and can be readily detected with FLIPR (Fluorescent Imaging Plate Reader) Calcium 6 fluorescent dye. In addition, we expected the Cd background activity to be lower than Mn as it is typically observed to be rarer in reagents. We observed a robust, time-dependent increase in Calcium 6 fluorescence upon addition of Cd in induced SLC39A8 HEK293-T-REx cells that was largely absent in control (noninduced) cells (Figure 2A). With this system, we screened a diverse collection of FDA-approved drugs (Prestwick Chemical Library) for molecules that increased Cd-induced fluorescence in cells expressing SLC39A8. As a result of screening, we identified efavirenz (EFV), a non-nucleoside reverse transcriptase inhibitor, as a putative pharmacologic potentiator of SLC39A8 based on its ability to increase Cd uptake in an SLC39A8-dependent manner (Figure 2, B and C).

To determine the relative selectivity of EFV for SLC39A8, the effects of EFV on the closely related metal transporters SLC39A4 and SLC39A14 were evaluated. In contrast to the findings with cells for inducible expression of SLC39A8, EFV did not enhance Cd uptake in cells expressing other SLC39A family members (Figure 2, C and D, and Supplemental Figure 2). We also assessed the ability of EFV to enhance Cd uptake in cells expressing various SLC39A8 orthologs. Our findings demonstrate that EFV was able to enhance Cd uptake in cells expressing mouse SLC39A8 but not chicken, frog, or zebrafish (Supplemental Figure 3, A–E). This suggests that although EFV is marketed as an inhibitor of HIV reverse transcriptase, it may have additional unanticipated effects potentiating metal uptake mediated by SLC39A8.

To further characterize the pharmacologic effects of EFV, the kinetics of Cd-induced fluorescence were measured during the early, linear phase of uptake as an estimate of initial uptake rate. A plot of the estimated initial rate against Cd concentration revealed that EFV (20 μM) increased the apparent maximal uptake rate (V*_max_*) while slightly reducing Cd affinity (*K_m_*) (Figure 2, B and D, and Supplemental Table 1). To confirm these initial observations, we evaluated the ability of EFV to enhance SLC39A8 activity in an orthogonal Mn uptake assay. HEK293-T-REx cells with inducible expression of SLC39A8 exhibited a robust, time-dependent quenching of calcein on addition of Mn (Figure 2, E and F) that was absent in noninduced cells (Figure 2E). Consistent with an effect on SLC39A8, EFV increased Mn-induced calcein quenching (Figure 2E). Due to the low affinity of Mn as a SLC39A8 substrate, the effects of EFV on *K_m_* and V*_max_* for Mn transport could not be determined (Figure 2F). Taken together, these results indicate that EFV potentiates Cd^2+^ and Mn^2+^ uptake by a mechanism that requires the expression of human or mouse SLC39A8. Since mutations in SLC39A8 have been linked to several severe human diseases, we were interested to know whether EFV could enhance function of these disease-relevant *SLC39A8* variants. Six different variants were transiently transfected into HEK293T cells and analyzed for function using the Cd uptake assay. Remarkably, EFV increased the function of each disease-relevant variant (Figure 2G). In several cases, EFV restored function to WT levels or above.

### Potentiation of SLC39A8 through an allosteric mechanism.

Homology models of human SLC39A8 were generated using the prokaryotic BbZIP crystal structure (Protein Data Bank [PDB] ID 5TSB) (22) to probe for potential EFV binding sites. As the sequence identity between hSLC39A8 and BbSLC39A8 is low (14% ID), a sequence alignment was constructed manually utilizing literature information (22) and functionally important amino acids that are highly conserved across the SLC family to guide residue correspondence (Supplemental Figure 4A). The resulting model demonstrates reasonable agreement with the overall tertiary structure, while major differences are localized to flexible loop regions (Supplemental Figure 4B). Utilizing the computational tools SiteMap, SiteFinder, and FTMap, the surface of the protein was mapped for both potential binding pockets and energetically favorable hot spots; the 6 identified sites are shown in Supplemental Figure 4C.

**Figure 3:**
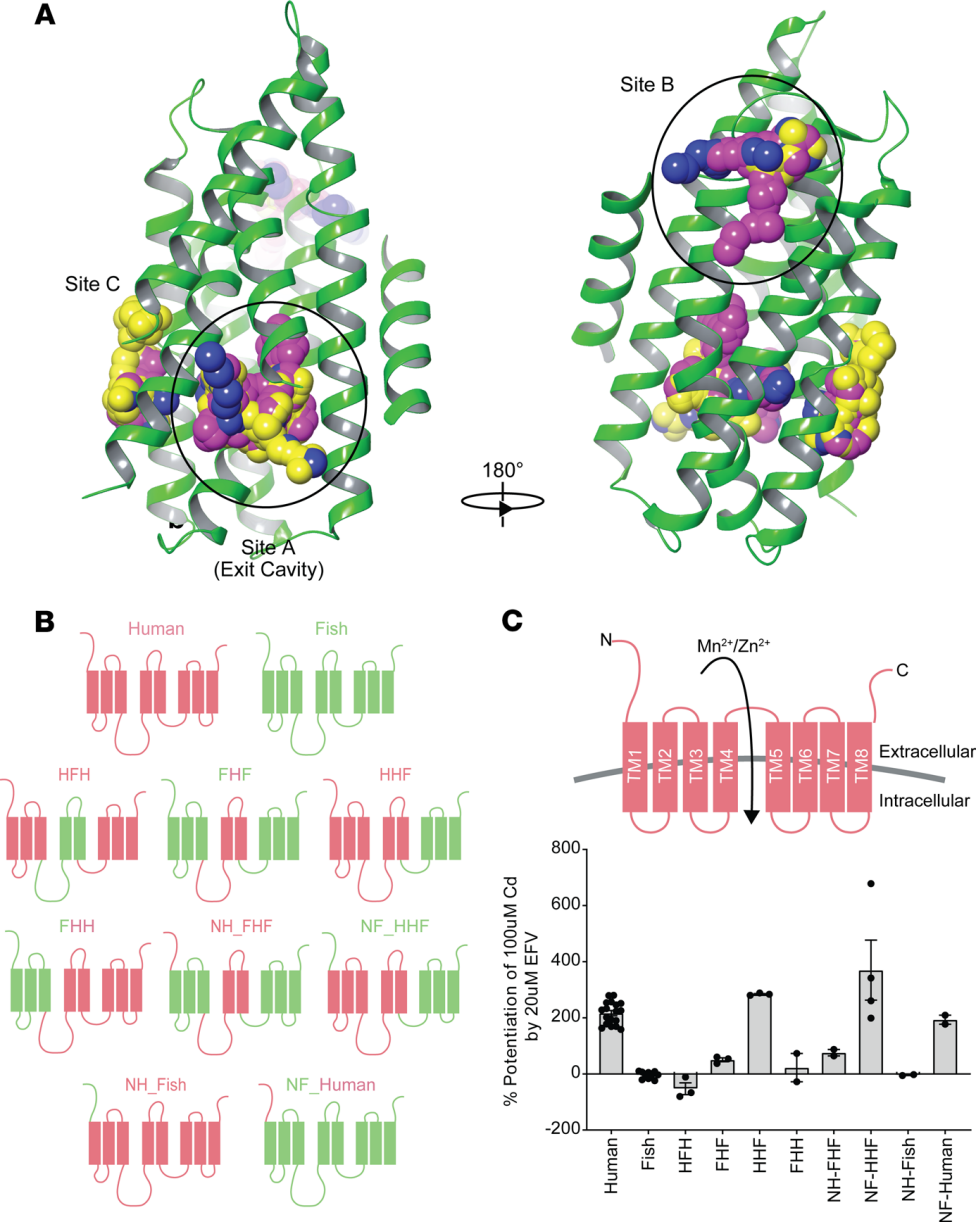
Homology model identifies potential binding pockets on SLC39A8, and mermaid (fish–human) chimeras identify TMDs required for EFV activity. (**A**) Three sites (sites A–C) were identified on the homology model of hSLC39A8 as the potential binding pocket for EFV. Site A corresponds to the putative metal exit cavity, while site B was predicted to be the EFV binding site based on experimental data. SiteMap spheres are shown in pink, SiteFinder spheres in blue, and FTMap probes in yellow. (**B**) Designed mermaid chimera constructs; human (H) TM domains are shown in red, while zebrafish (F) are green. N indicates N-terminus region of the indicated species (H or F). (**C**) Impact of mermaid chimeras on EFV potentiation on SLC39A8 (average of *n* = 2–18 experiments). Data are presented as mean ± SEM.

While 6 potential sites were identified, only 3 of the sites (sites A–C) were predicted by all 3 computational tools utilized (Figure 3A and Table 1). We commonly find that sites identified by multiple methods are more likely to be real binding pockets (35), so we hypothesized that the EFV binding pocket was likely site A, B, or C. The putative metal exit cavity (site A) was ranked the most ligandable pocket by all computational methods (Figure 3A), which was not surprising given the inward-open conformation of the model. To probe whether it is the EFV binding pocket, nonconserved residues in site A among human, chicken, frog, and zebrafish were selected for subsequent mutagenesis studies considering the species selectivity of EFV that we observed. The mutagenesis data revealed that mutations in this site did not alter EFV sensitivity, suggesting that this is not the EFV binding site (Supplemental Figure 5). Due to this finding, our study focused on site B or C as the putative binding site.

We exploited our observation that EFV does not potentiate zebrafish SLC39A8 and generated 8 human–zebrafish SLC39A8 chimeras (“mermaid” chimeras). Initially, we divided the predicted human protein into N-terminus plus 3 transmembrane regions (N, TM1/2/3, TM4/5, and TM6/7/8; Figure 3B), replaced each region with the corresponding zebrafish region, and measured the ability of EFV to potentiate Cd uptake. Maximal EFV effect required human TM1/2/3, and a partial effect was observed in chimeras containing human TM4/5 (Figure 3C). These data suggest that a putative EFV binding site could be in the N-terminal half of the SLC39A8 protein, potentially distributed across TM1/2/3 and TM4/5 of human SLC39A8.

Structural data revealed that site B is at the interface of TM1, TM5, and the TM4/5 loop region, while site C is located at the interface of TM4 and TM6. Taken in context with the experimental data described above, site B appeared to be the more likely site of the 2 remaining possibilities. In further support of site B, there are multiple residue differences between human and zebrafish making up the predicted pocket: Y131A, S125A, L334F, and C326F (human–zebrafish). Site B was identified by SiteMap as the fourth-ranked pocket with SiteScore of 0.61, by SiteFinder as the second-ranked pocket with a propensity for ligand binding (PLB) score of 0.72, and was also mapped by FTMap, with 7 different probe types, suggesting the presence of hot spots. Based on benchmark work for all 3 methods (36–39), this pocket in its modeled apo conformation does not meet strict criteria to be considered ligandable. However, a challenge when utilizing apo structural information is that pockets that are not well defined or cryptic in nature can be difficult to predict by traditional binding site identification tools. Furthermore, even if these sites are identified, their evaluation can be skewed, and they may be ranked quite low as their size, functionality, and solvent exposure are generally not optimized for ligand binding. Given this, and together with functional data from mermaid chimeras, we considered site B a viable candidate EFV binding site.

**Figure 4:**
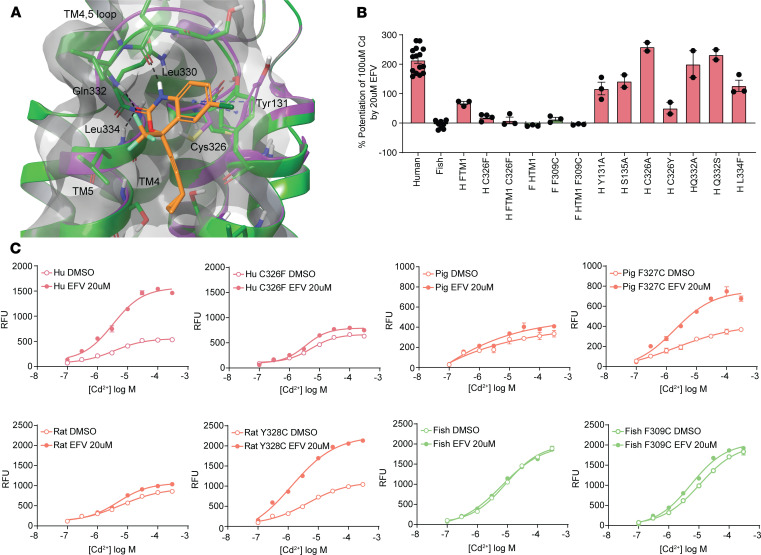
IFD and point mutation studies predict EFV fits in the TM1/4/5 loop region with key residues. (**A**) Predicted docking model for EFV (orange) utilizing IFD. The initial protein conformation from a homology model is shown in purple, and the induced fit is in green. Black dotted lines illustrate important hydrogen bonds between the EFV and protein, and blue indicates a π–π interaction. (**B**) Point mutations identifying key residues in site B that contribute to EFV potentiation of Cd uptake (average of *n* = 1–15 experiments). Data are presented as mean ± SEM. (**C**) Species with large, bulky residues at the equivalent position to C326 in human SLC39A8 (rat and pig) were insensitive to EFV, and EFV sensitivity was created by mutation to cysteine. *n* = 3 replicates; data are presented as mean ± SEM. RFU, relative fluorescence units; H, human; F, zebrafish; HFTM1, fish transmembrane region 1 on a human background; FHTM1, human transmembrane region 1 on a fish background.

A binding model of EFV in site B was predicted using induced fit molecular docking (IFD) studies (Figure 4A). While it is highly likely that the pocket is not fully optimized and would undergo additional rearrangement in the presence of EFV, the predicted binding pose has a reasonable docking score and forms multiple interactions with the protein. Three hydrogen bonds are predicted between EFV and the backbone atoms of Leu330, Gln332, and Leu334, and the side chain of Tyr131 reorients to optimize π–π stacking with aromatic rings of EFV. Interestingly, at the back of the proposed binding site is Cys326, which undergoes substantial rearrangement in the IFD and moves away from the binding site to accommodate EFV binding. Additionally, the TM4,5 loop folds down slightly.

Molecular dynamics (MD) simulations were run to determine if the predicted binding pose from IFD was energetically stable. After 700 ns simulations, we found that the ligand stayed in the pocket in 2 of the 3 replicates we simulated (Supplemental Figure 6, A and B). In the replicate where the ligand escaped, the unbinding happened after 40 ns of the second stage of the simulation (no restraints between the ligand and protein), which is much longer than the common length used for examining binding poses (15 ns). Given the length of the total simulation time, we can say with confidence that the ligand remains bound in a stable fashion to this pocket. However, it was necessary for the binding site to “induce fit” around the ligand for the ligand to remain bound. Lastly, the SiteScore for site B increased from 0.61 (homology model) to 1.28 (last snapshot of MD simulation), indicating that the conformational sampling by MD created a more ligandable pocket (a score greater than 1.0 suggests the site is promising as a binding pocket) (Supplemental Figure 6C) (37, 38).

Utilizing the predicted binding mode, residues were identified for subsequent mutagenesis studies based on both differences between species (zebrafish, chicken, frog, and mouse) and the predicted binding interactions. For residues within 4 Å of the predicted EFV pose, all residues were conserved within mouse, but there were 4 differences in zebrafish (Q332S, C326F, L334F, and S135A), 2 in chicken (C326F and S135A), and 1 in frog (C326F). Mutants based on the binding mode and species differences were generated to test the binding site hypothesis, and the data support the computational model (Figure 4B). Tyr131 and Ser135 (alanine in zebrafish and chicken) and Leu334 (phenylalanine in zebrafish) play key roles in EFV activity, supporting their role in a putative binding site. Additionally, while small side chain groups were tolerated, bulky substituents were detrimental at Cys326 (phenylalanine in zebrafish, chicken, and frog), suggesting a steric effect. Interestingly, reverse mutations did not impart EFV sensitivity to zebrafish SLC39A8, suggesting that additional key EFV residues in site B may be involved.

Additional species sequences were examined to identify others with large, bulky residues at the homologous 326 position to test our hypothesis that Cys326 forms part of an EFV binding site and the ability of cysteine to reorient away from the binding site drives species selectivity, as seen in the IFD and the MD simulation (Supplemental Figure 6D). Rat and pig were found to fit this criterion, with a tyrosine and phenylalanine at the homologous 326 position, respectively. As predicted, both rat and pig SLC39A8 were EFV insensitive, and EFV sensitivity could be created by replacing these residues with cysteine in pig F327C and rat Y328C, respectively (Figure 4C).

The data support a model where EFV binds to SLC39A8 by wedging itself between TM1 and TM5 and underneath the TM4/5 loop to potentiate the transporter activity of SLC39A8. While an unequivocal demonstration of direct binding requires biophysical assays or structural data such as cryo–electron microscopy or crystallography, site-directed mutagenesis is a widely utilized method for identifying residues important for ligand binding. Utilizing a combination of computational techniques and experiments allowed for the identification of the binding pocket and demonstrated target engagement between EFV and SLC39A8.

### Initial SAR evaluation of SLC39A8 potentiators.

**Figure 5:**
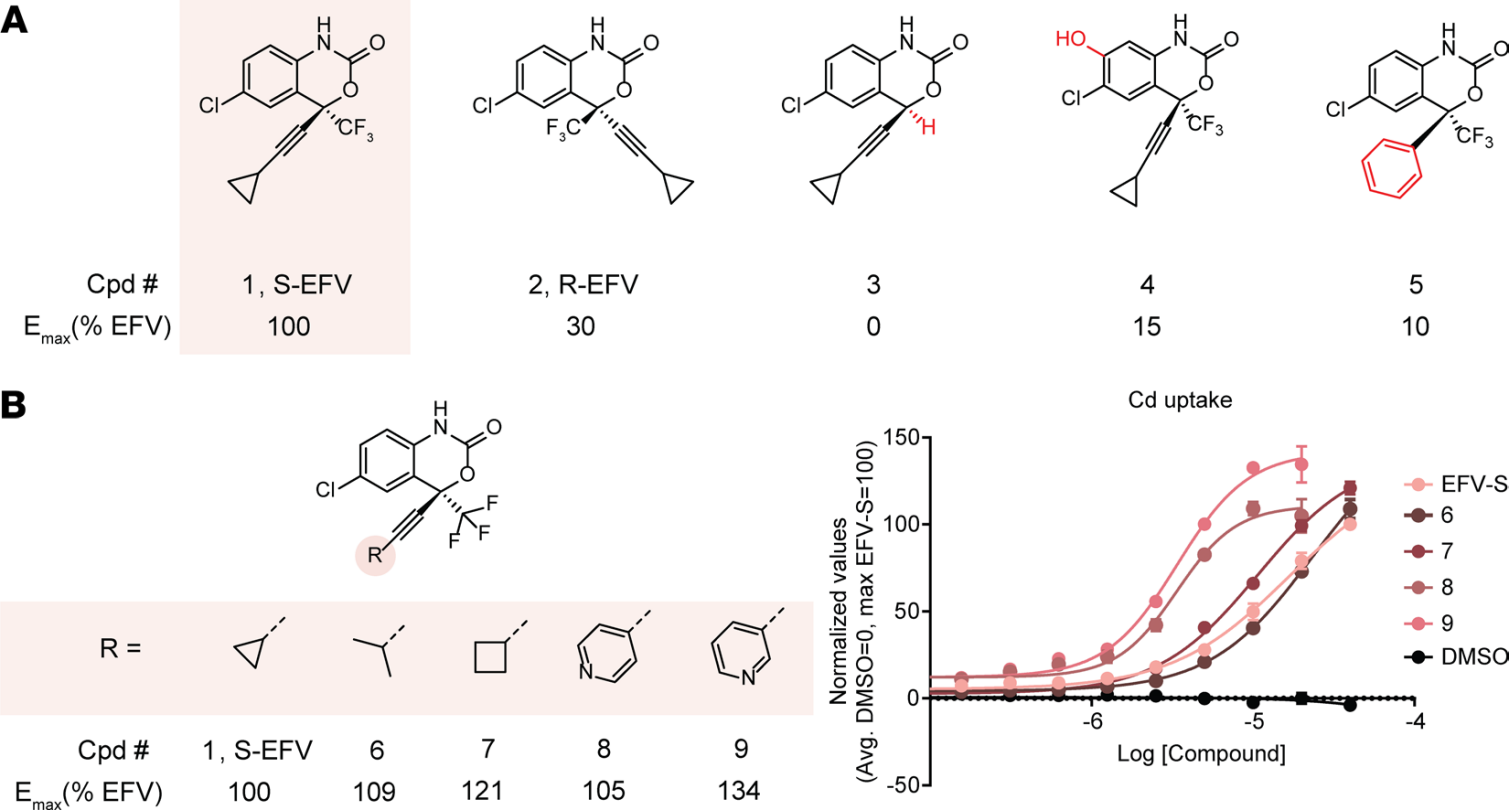
SARs of EFV analogs. (**A**) Comparison of active compounds with changes that decrease activity. (**B**) SARs of EFV analogs inducing Cd uptake. *n* = 4–8; data are presented as mean ± SEM. Stereochemistry is tentatively assigned.

A series of close analogs of EFV were synthesized to identify key structural features for potentiation of Cd uptake and to establish structure-activity relationships (SARs) for potency (EC_50_) and efficacy (E_max_) (Figure 5A). Initial SARs demonstrated that the bicyclic core region of EFV (1) was essential for its potentiation activity at SLC39A8. This aligned with the predicted binding mode where a π–π interaction between Tyr131 and the bicyclic core of EFV was observed throughout >70% of the MD simulation, demonstrating its importance. Inversion of the stereochemistry diminished the E_max_ by 70% (2), and removal of the trifluoromethyl group (3) eliminated potentiation activity, potentially through loss of a key lipophilic interaction with Leu334 based on the MD binding model. Substitution of the aromatic ring (4) also substantially decreased potentiation activity. The EFV alkyne is predicted to occupy a narrow region of the pocket surrounded by TM helices 1, 4, and 5, and replacement with a phenyl (5) decreased potentiation activity by 90%.

The predicted binding pose of EFV indicated that the subpocket between TM4 and TM5 could accommodate larger substituents than the alkyne cyclopropyl group for potency optimization. Opening of the cyclopropyl ring to an isopropyl group (6) was tolerated, and substitution with a larger cyclobutyl substituent (7) caused an encouraging increase in potency and maintained E_max_. The binding pocket occupied by the cyclopropyl group in the proposed binding mode contains residues capable of making hydrogen bond–donating interactions with the ligand including Ser337 on TM helix 5 and Ser135 on TM helix 1. The EFV cyclopropyl group was replaced with 4-pyridyl (8) and 3-pyridyl (9) substituents as hydrogen bond acceptors, and isomer 9 was identified as the preferred isomer, resulting in an increase in both potency at potentiating Cd uptake and E_max_ (134%) relative to EFV (Figure 5B). These studies defined SARs for EFV analogs that are consistent with the docking model and produced a SLC39A8 potentiator (9), demonstrating improved potency and E_max_ relative to EFV.

### Effect of SLC potentiators of epithelial barrier function.

**Figure 6:**
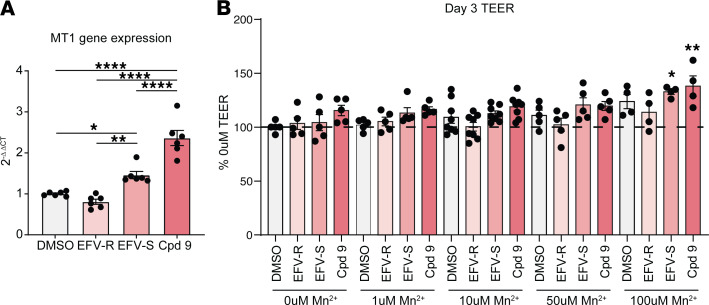
EFV-S and compound 9 increase *MT1* gene expression and TEER in primary murine IECs. (**A**) Differentiated WT murine IEC monolayers treated with DMSO or 20 μM compound for 24 hours. Reverse transcription qPCR analysis for *MT1* is shown as fold change (2^–ΔΔCT^) compared with DMSO control group within each genotype. Data are presented as the mean of 3 independent experiments (*n* = 6 per group). One-way ANOVA; *P* < 0.0001. Tukey’s multiple-comparison test; *P = 0.032, **P = 0.0019, ****P < 0.0001. Data are presented as mean ± SEM. (**B**) Differentiated WT murine IEC monolayers treated with various concentrations of exogenous Mn for 48 hours in the presence or absence of 10 μM EFV-R, EFV-S, or compound 9. TEER measurements (Ω*cm^2^) for the untreated control group were set to 100%, and other treatment groups were normalized to the control group. Data are presented as the mean of 4 independent experiments (*n* = 4–8 per group). One-way ANOVA; *P* < 0.0001. Šidák’s multiple-comparison test. Asterisks indicate groups that differ significantly from the 0 μM Mn DMSO group; *P = 0.0131, **P = 0.0010. Data are presented as mean ± SEM.

It has previously been shown that the A391T missense variant of *SLC39A8* leads to Mn deficiency in the mouse colon associated with impaired intestinal barrier function and persistent interaction with commensal microbes that prime inflammation (15). We hypothesized that EFV may conversely promote epithelial barrier function, based on its ability to potentiate Mn uptake in HEK293T cells. To model the intestinal barrier, we established primary intestinal epithelial cell (IEC) cultures and determined if EFV treatment can elicit expression of the metal response gene metallothionein 1 (*MT1*). MT1 is involved in the homeostasis of free intracellular Zn/copper, detoxification of heavy metals such as Cd/mercury, and free radical scavenging. In response to increased influx of Zn into the cytoplasm, metal-responsive transcription factor 1 translocates into the nucleus to act as a transcription factor at metal response elements upstream of *MT1* and other genes (40). WT mouse IEC monolayers were treated both apically and basolaterally with DMSO, 20 μM EFV-R, 20 μM EFV-S, or 20 μM compound 9 for 24 hours before harvesting cells for RNA. Gene expression analysis showed that MT1 was upregulated about 1.5-fold with EFV-S and over 2-fold with compound 9, but not with EFV-R, compared with DMSO (Figure 6A). This is in line with our observation that EFV-S and Compound 9 can potentiate the activity of SLC39A8, whereas EFV-R (Compound 2) is an inactive enantiomer (Supplemental Figure 7).

To more directly define the effects of EFV on barrier function, we established and utilized primary IEC monolayer cultures. This culture system allows for the formation of a 2-dimensional planar structure with tight junction assembly that impedes solute diffusion in a manner that can be quantified by measuring transepithelial electrical resistance (TEER). WT mouse IECs were seeded in Transwell inserts and differentiated on day 1 after seeding. Cells were treated with various concentrations of Mn in the presence or absence of DMSO, 10 μM EFV-R, 10 μM EFV-S, or 10 μM Compound 9 starting on day 1. Treatments were applied both apically and basolaterally. TEER was measured on day 3. In these cells, the highest concentration of exogenous Mn tested (100 μM) led to an increase in TEER (Figure 6B). While EFV-S alone did not modulate TEER, EFV-S treatment in the presence of 1, 10, or 50 μM Mn led to a trending increase in TEER. Compound 9 showed a trending increase in TEER in the presence or absence of exogenous Mn. Importantly, the inactive enantiomer EFV-R did not show this same increase in TEER. Taken together, our results suggest that modulation of Mn uptake with small-molecule potentiators may restore Mn homeostasis. Moreover, we establish the feasibility of potentiating SLC39A8 by exploiting a binding pocket.

## Discussion

Due to their core functions in transporting solutes across cell membranes, SLC transporters control a wide variety of essential cellular functions. Accordingly, they represent vulnerabilities to the development of disease arising from genetic diversification and evolutionary selection pressures. Genetic variants of SLC transporters have been linked to Mendelian inheritance of a variety of rare diseases, while more common variants have been identified in GWASs of several prevalent diseases (41, 42). For these reasons, SLC transporters have become a subject of increasing interest for the purposes of drug development (43, 44). To date, several SLC inhibitors have been granted approval for clinical use, although they target a small fraction of the potentially druggable SLC family and mainly consist of neurotransmitter reuptake inhibitors for neuropsychiatric disorders, diuretics to control blood pressure, and glucose transport inhibitors to treat diabetes (30, 45). An abundance of targeting strategies for SLC transporters have emerged at various stages of clinical development, expanding the scope of applications to immune-mediated diseases, among others (30, 46, 47). These examples establish the tractability of SLC transporters as drug targets, and proteome-wide ligandability mapping with chemoproteomics approaches has provided further support for the druggability of this target class (48). While most established targeting strategies inhibit SLC transport function, it is theoretically possible to leverage small molecules to potentiate substrate transport and thus reverse a functional defect that may arise from genetics or cellular dysfunction. Here, we identify a potentiator of SLC39A8-mediated cellular uptake of Mn. The approach was guided by insights from human genetics demonstrating that loss of function variants of *SLC39A8* linked to CDG and Leigh syndrome and common variants linked to autoimmune, neurologic, and metabolic diseases reduces systemic Mn levels (4–6, 32). Thus, potentiating SLC39A8 may have a therapeutic benefit in reestablishing Mn homeostasis across several human pathologies.

SLC39A8 belongs to a subfamily of transporters of divalent metal ions, although it specifically regulates cellular uptake of Mn, Zn, iron (Fe), and Cd, with higher affinity for Mn than Zn and Fe (17, 49, 50). Here, we performed a drug repurposing screen and identified EFV as a potentiator of Cd and Mn transport into cells. Originally developed and currently used to treat HIV-1 infection, EFV is a non-nucleoside reverse transcriptase inhibitor (51, 52). Given its activity as a potentiator of SLC39A8, it is possible that at least a subset of reported side effects could be due to its ability to modulate Mn homeostasis. The most common side effects of EFV are skin reactions and neuropsychiatric adverse events that are typically transient (53). These acute neuropsychiatric symptoms are thought to be due to effects of EFV on the serotonergic system (54, 55), whereas other delayed neurological symptoms such as ataxia and psychomotor impairment are not completely understood (56, 57). Notably, neurological symptoms and movement disorders have been attributed to hypermanganesemia brought about by environmental or occupational exposure, contaminants from illicit drug use, or genetic disorders (11, 58). Rare genetic variants in *SLC30A10* or *SLC39A14* have been linked to hypermanganesemia with dystonia and neurotoxicity (59–64). Conversely, genetic variants of SLC39A8 have been linked to hypomanganesemia and a host of physiological perturbations ranging from severe developmental disorders to Parkinson’s disease, IBD, and cardiovascular disease (4–6, 32, 34, 65–67). These strong genetic links between Mn levels and human disease provide further context and rationale for targeting SLC39A8.

Despite widespread interest and rationale from genetics to target SLC transporters, there remain generalizable challenges. The scope of functional diversity within the SLC family, along with paralog redundancy, creates obstacles for establishing quantitative and high-throughput assays. Progress in the field has come from screening strategies to identify paralog-specific inhibitors of SLC transporters (68). Similarly, we developed a phenotypic screen for SLC39A8 potentiators by employing fluorescent intracellular reporters for metal ions and identified EFV as selective for SLC39A8 over its paralogs SLC39A4 and SLC39A14. Another challenge in targeting SLC transporters has been a lack of structural and biophysical data to assist in optimization of chemical probes, although considerable progress has been made employing x-ray crystallography, cryo–electron microscopy, and computational modeling (69–71). Here, we utilized a hybrid approach to define the EFV binding site on SLC39A8. This was achieved through iterative structural modeling, MD simulations, and functional mutagenesis.

The structure utilized for SLC39A8 was a homology model built from the prokaryotic BbZIP crystal structure (22, 23). We used this structure in conjunction with computational tools to identify the EFV binding site, establish direct binding to SLC39A8, and enable compound optimization. However, only apo structural information was available, and this can cause a challenge for pocket detection as some binding sites are not available in the apo state and only become present in the holo state. By combining approaches, we were able to filter 6 putative EFV sites down to 2 likely candidates. Incorporating experimental mermaid chimera data was necessary to further guide selection of the EFV binding site from the last 2 remaining sites. Deeper analysis combining molecular docking guided the residues for selection in the mutagenesis studies, while the mutagenesis data provided further support that site B is the EFV binding site. This integrated approach facilitated iterative feedback, with insights from experiments shaping model modifications, while computational predictions steered the design of future experiments. Such multidisciplinary or hybrid approaches allow for viewing systems from multiple vantage points and can augment modeling of small-molecule binding sites on target proteins.

Taken together, our data suggest that EFV binds in an induced pocket at the interface of TM1/4/5. In the apo state, the pocket was identified but not considered to be ligandable based on benchmark studies for the 3 methods utilized. Employing MD can be a useful tool for conformational sampling to identify changes necessary to accommodate ligand binding. We examined snapshots from the MD simulation of SLC39A8 in complex with EFV to assess its ability to sample the protein and open the pocket. We then observed that site B was considered ligandable, as the ranking and scores improved from conformational sampling (Supplemental Figure 5C). This provided additional evidence that site B requires conformational changes to accommodate EFV binding. Given that the structure utilized for these studies is a homology model built from the prokaryotic BbZIP crystal structure and the conformational flexibility of SLCs, this is not a surprising finding and illustrates the utility of introducing dynamics to further understand the system.

We sought to determine whether the EFV binding site B would have been identified if the predicted AlphaFold (72, 73) model of SLC39A8 had been used rather than the homology model described above. Utilizing SiteMap and SiteFinder, we found that site B would not have been identified with the AlphaFold model (Supplemental Figure 8A). An overlay of the AlphaFold- and Prime-generated models is provided in Supplemental Figure 8B, and there is much overlap between the conformations with a root mean square deviation (RMSD) of 3.314. However, upon closer examination, it is apparent that the TM4/5 loop is shorter in the AlphaFold model, as residues making up the loop in the Prime model are predicted to be part of the helical secondary structure of TM4 in the AlphaFold model. Additionally, residue side chains occupied the pocket in the AlphaFold model. Specifically, Leu330 completely overlapped with EFV in its predicted binding pose (Supplemental Figure 8C). This illustrates the necessity of incorporating as much structural information as possible, whether in the form of multiple crystal structures or snapshots from MD simulations, when assessing targets for potential binding sites.

It is notable that EFV directly potentiates SLC39A8 through binding to a cryptic pocket, which raises several mechanistic questions more generally relevant to positively modulating transporter activity. Structural analyses indicate that ZIP family transporters exhibit a 2-domain architecture comprising a scaffold domain and a transport domain (23, 26, 27, 74). Whereas the scaffold domain (TM2, TM3, TM7, or TM8) remains static, the transport domain (TM1, TM4, TM5, or TM6) undergoes a conformational switch from an IFC to an outward-facing conformation (OFC), allowing for transport through an elevator-type mechanism. Furthermore, comparisons of the metal-free and -bound states of the IFC suggest that the domains move as rigid bodies in relation to one another in a metal-dependent manner (23). We propose that EFV binding to a cryptic pocket in the transport domain (Supplemental Figure 9, A and B) may lower the activation energy for actuating the elevator mechanism by stabilizing a conformation that shifts the equilibrium to promote switching between the IFC and OFC. This mechanism of action may represent a viable approach for potentiating other transporters, given that the elevator-type mechanism is thought to represent a conserved strategy amongst the ZIP family for transporting metal ions across cell membranes (23, 28). With this insight, future work optimizing the pharmacokinetic properties of compound 9 will be valuable in the development of in vivo probes to modulate cellular Mn uptake. Taken together, our findings reinforce the notion of SLC transporters as promising drug targets and, more importantly, demonstrate the feasibility of potentiating their functions with small molecules.

## Methods

### Experimental methods

#### Sex as a biological variable.

For mouse-derived IEC spheroid studies, described below, sex was not considered as a biological variable.

#### Materials.

Culture media, serum, antibiotics, zeocin, blasticidin, and buffers for cell culture were obtained from Thermo Fisher Scientific. EFV was purchased from Sigma-Aldrich (catalog SML0536) and prepared as a 20 mM stock solution in DMSO. Cd and Mn were prepared as 1 M stock solutions in distilled water. All drug dilutions were made in assay buffer on the day of the experiment. All cDNA constructs were synthesized by GenScript and cloned into pcDNA3 or pcDNA4/TO (Invitrogen).

#### Cell culture and stable cell line generation.

HEK293T (ATCC; CRL-3216) and HEK293-T-REx cells (Thermo Fisher Scientific; R71007) were maintained at 37°C in a humidified 5% CO_2_ incubator in culture medium (DMEM supplemented with penicillin [100 U/mL], streptomycin [100 mg/mL], and 10% tetracycline-free FBS) plus zeocin (200 μg/mL) for HEK293-T-REx. For transient expression, HEK293 cells were transfected using Lipofectamine 2000 (Thermo Fisher Scientific; 11668019) according to the manufacturer’s instructions and maintained in growth medium for 48 hours. For stable expression, cells were transfected with pcDNA4-TO expression constructs using Lipofectamine 2000 (Promega) according to the manufacturer’s protocol and maintained in selection medium containing 200 μg/mL zeocin and 5 μg/mL blasticidin. Stable pools of antibiotic-resistant cells were maintained under constant selection, and transporter activity was confirmed in the Cd uptake assay after overnight incubation with 1 μg/mL tetracycline.

#### Transient transfection and flow cytometry analysis of SLC39A8 disease mutants.

HEK293T cells (5 × 10^6^) were transfected with 5 μg DNA using Lipofectamine 2000 and incubated for 48 hours at 37°C, 5% CO_2_. Constructs of the various N-terminal FLAG (DYKDDDDK)–tagged mutants can be found in the Supplemental Methods. Cells were then harvested and stained for flow cytometry analysis. Briefly, cells were washed, resuspended at 1 × 10^6^ cells/mL, and transferred into 1 mL aliquots in Eppendorf tubes. After centrifugation at 2,000*g* for 5 minutes, media was removed and cells resuspended in 100 μL assay buffer (HBSS with 20 mM HEPES plus 0.5% BSA) and incubated for 15 minutes. The anti-FLAG–Alexa647 antibody (Cell Signaling Technology; 3916) was added at 1:100 and incubated for 1 hour at room temperature in the dark. Cells were washed and resuspended in 1 mL of buffer, into which FluoZin-3 cell permeant Zn indicator dye (Thermo Fisher Scientific; F24195) was added at 1:1,000 dilution for 30 minutes at room temperature in the dark. Cells were washed twice and transferred to tubes for FACS on the FACSCanto (BD Biosciences). Data were analyzed using FlowJo software (v10.8.1)

#### Functional assays.

Stable HEK293-T-REx cells (human SLC39A8, SLC39A4, and SLC39A14) were removed from the culture flask; plated at 20,000 cells/well in poly-d-lysine–coated, black, clear-bottom, 384-well plates (BioCoat; Corning); and induced overnight with 30 ng/mL tetracycline. On the day of the experiment, cells were washed twice with assay buffer (HBSS; Corning) to remove culture medium. Transiently transfected cells were removed from the culture dish, plated at 40,000 cells/well in poly-d-lysine–coated, black, clear-bottom, 384-well plates (BioCoat), and allowed to settle for 1 hour at 37°C. Cells were loaded with either FLIPR Calcium 6 fluorescent dye (Molecular Devices) for 1 hour at 37°C to measure Cd uptake or with calcein-AM (Thermo Fisher Scientific) (final concentration, 0.5 mM) for 30 minutes at room temperature to measure Mn uptake. Plates were read on the FLIPR Tetra screening system (Molecular Devices) to measure emission at 515–575 nm, after excitation at 470–495 nm. Compounds were added on-line (12.5 μL of 5× compound solution in HBSS) with addition of Cd or Mn (12.5 μL of 6× solution) after 5 minutes, and fluorescence was measured for an additional 5 minutes.

Experiments were performed in triplicate or quadruplicate at least twice for each condition, with data presented as averages ± SEM. Metal-induced changes in fluorescence were measured at the earliest time point when the metal response could be clearly distinguished from any addition artifacts (typically 30–60 seconds after metal addition).

#### Establishment of mouse IEC spheroids.

Mouse colonic spheroids were established using previously described methods (75) from C57BL/6NTac-Slc39a8tm1a(EUCOMM)Wtsi/Cnrm mice carrying a targeted allele of Slc39a8 (obtained from the laboratory of Daren Knoell, University of Nebraska Medical Center, Omaha, Nebraska, USA). Briefly, a 2 cm^2^ piece of mouse colonic tissue was harvested and rinsed with cold PBS to remove debris. The tissues were minced with fine scissors in a 35 mm petri dish and incubated with 1 mL collagenase solution for 30 minutes, with intermittent pipetting. The released cells were passed through a 70 μm cell strainer, rinsed with 9 mL of washing medium, and centrifuged at 200*g* for 5 minutes. The cell pellet was gently resuspended in 1 mL of washing medium, transferred to a 1.5 mL Eppendorf tube, and centrifuged at 300*g* for 5 minutes. After removing the supernatant, the remaining cell pellet was resuspended in Matrigel extracellular matrix and seeded in 24-well plates (30 μL/well) on ice. A pipette tip was used to spread the Matrigel droplet in the center of the well without touching the walls. Plates were then taken off ice, flipped upside down, and incubated at 37°C for 10 minutes to allow the Matrigel to solidify with the cells suspended within. Next, 50% L-WRN medium (Analytical Biological Services, C01-0770) containing 10 μM Y27632 was added (500 μL/well), and cells were incubated at 37°C. Media was changed every 2 days (50% L-WRN without additional inhibitors) until cells were ready for passage.

#### Passage and expansion of spheroids.

Cells were passaged according to the protocol of Miyoshi and Stappenbeck (75). Briefly, media was aspirated from wells, and cells and Matrigel from 3 wells were resuspended in 800 μL of PBS by vigorous pipetting and then transferred into a 15 mL conical tube (combining a maximum of 18 wells in 1 conical tube). Cells were centrifuged at 300*g* for 5 minutes, and supernatants were aspirated.

Five milliliters of 0.5 mM PBS-EDTA solution was added to each conical tube, which was then vigorously shaken by hand to help break up the Matrigel (~10–15 times). Cells were centrifuged at 300*g* for 5 minutes, and supernatants were carefully aspirated.

One milliliter of 1× trypsin-EDTA was added to each conical tube, and cells were resuspended by gentle pipetting. Cells were incubated at 37°C for 2 minutes and then 1 mL of washing media was added to each conical tube to neutralize the trypsin. Cells were then vigorously pipetted 10–15 times and passed through a 70 μm strainer into a fresh 15 mL conical tube. Cells were centrifuged at 300*g* for 5 minutes, and supernatants were aspirated.

Cell pellets were gently resuspended in 1 mL of washing media and transferred to a 1.5 mL Eppendorf tube. The tube was placed in an empty 50 mL conical tube and centrifuged at 300*g* for 5 minutes. The supernatant was removed by pipetting, the remaining pellet was resuspended in Matrigel extracellular matrix, and cells were seeded in 24-well plates (30 μL per well) on ice. A pipette tip was used to spread the Matrigel droplet in the center of the well without touching the walls.

Plates were then incubated upside down at 37°C for 10 minutes to allow for the Matrigel to solidify with the cells suspended within. Then, 50% L-WRN medium containing 10 μM Y27632 was added (500 μL per well), and cells were incubated at 37°C. Media was changed every 2 days (50% L-WRN without additional inhibitors) until cells were ready for passage, at a 1:4 to 1:8 ratio.

#### Seeding submerged Transwell monolayers.

To prepare cells to seed into Transwells, the passaging protocol (above) was utilized with the following modifications (76). After trypsinization, cells were pipetted 100 times and passed through a 40 μm strainer. After washes, cells were resuspended in 50% L-WRN + 10 μM Y27632 medium and seeded in the apical compartment of Transwell inserts (Corning 3413) that were precoated in attachment factor (Thermo Fisher Scientific; S006100). One hundred microliters of cells was added to the apical compartment, and 600 μL of medium was added to the basolateral compartment. Depending on the density, ~3–6 wells of spheroids (~2 × 10^5^ cells) were combined into 1 Transwell insert.

#### Differentiation of submerged Transwell monolayers.

On day 1 after seeding, media was changed to various differentiation media as required by the assays. The 600 μL of medium in the basolateral compartment was aspirated. The apical compartment medium was gently pipetted 2–3 times to resuspend any detached cells and then discarded. Two hundred microliters of differentiated medium (10 μM DAPT and 1 μg/mL LPS, in primary culture medium) was added to the apical compartment and 600 μL to the basolateral compartment. Compound/metal ion treatments were added on day 1 after seeding for TEER and on day 2 after seeding for RNA readouts. Cells were harvested on day 3.

#### TEER measurements.

An EVOM2 epithelial volt/ohm meter (World Precision Instruments) was used to measure TEER to assess barrier integrity of the Transwell monolayer daily starting day 1 after seeding. Transwell plates were placed in the tissue culture hood for 15 minutes to normalize temperature. EVOM2 chopstick probes were first washed in 70% ethanol, air-dried, and then serially washed in 4–5 wells of primary culture medium (~600–800 μL medium in a 24-well plate). Probes were then inserted vertically into the Transwells so that the shorter probe was suspended in the apical compartment (submerged in medium) and the longer probe was in the basolateral compartment (touching the bottom of the plate). Probes are held vertically, and the resulting resistance was measured in ohms. The probes were then serially washed in wells of primary culture medium before measuring the next Transwell. Note: the resistance of a blank Transwell (no cells) in the same medium was also measured each time to subtract the resistance conferred by the Transwell membrane from the total resistance.

For TEER calculations, the resistance (R) of a blank (no cell) Transwell was subtracted from the resistance of the experimental wells (cells + membrane) to obtain the resistance of the cells. This was then multiplied by the growth area of the Transwell insert (0.33 cm^2^ for Corning 3413) to get the R*A (Ω*cm^2^).

#### RNA extraction.

Media was removed, and apical and basolateral compartments of the Transwell inserts were gently washed with PBS. The Transwell insert was then securely placed in a prelabeled 1.5 mL Eppendorf tube. Using a p1000 pipette, 200 μL of Qiazol reagent (Qiagen; 79306) was added directly to the cells in the apical compartment and gently pipetted while scraping the membrane to lyse and resuspend cells. Once most cells were lysed, the pipette tip was used to puncture the membrane and transfer the cell lysate into the Eppendorf tube below. The insert was then discarded, and the lysates were frozen at –80°C until later use.

RNA extraction was performed according to manufacturer’s instructions using the Direct-zol RNA mini prep kit (Zymo Research; R2052). RNA was eluted in 25 μL water, and concentrations were measured by NanoDrop spectrophotometer (Thermo Fisher Scientific).

#### Reverse Transcription qPCR/low-density array.

One hundred nanograms of RNA/sample was used for cDNA synthesis using the High-Capacity cDNA Reverse Transcription kit (Thermo Fisher Scientific; 4374966) in a total volume of 20 μL. The cDNA synthesis PCR cycle was as follows: step 1, 10 minutes at 25°C; step 2, 120 minutes at 37°C; step 3, 5 minutes at 85°C; and step 4, hold at 4°C.

Quantitative PCR (qPCR) was performed in the TLDA (TaqMan Low-Density Array) platform from Thermo Fisher Scientific. For each sample, 20 μL of cDNA was combined with 30 μL water and 50 μL of the 2× TaqMan universal PCR master mix (Applied Biosystems; 4304437). The mix was then pipetted into a single port of the TLDA card. Once all samples were added, the card was centrifuged at 331*g* for 5 minutes. The individual wells were then sealed using a card sealer, and the sample ports were cut off along the edge of the card. The card was then run using the Quant Studio 12K Flex machine with qPCR cycling protocol as follows: step 1, 2 minutes at 50°C; step 2, 10 minutes at 95°C; step 3, 15 seconds at 95°C; and step 4, 1 minute at 60°C; repeat steps 3–4 for 40 cycles. MT1 The TaqMan primer/probe set ID is Mm00496660_g1. Data were analyzed using the Applied Biosystems Expression Suite Software.

### Computational methods

#### Homology model generation.

The homology modeling tool Prime (77, 78), from the Schrödinger suite of programs (79), was used to generate a model of the IFC and OFC of human SLC39A8 using default parameters. A Basic Local Alignment Search Tool (BLAST) (80) search was conducted in the PDB (81) using the SLC39A8 sequence to determine which homologs were available in the public domain with structural information. At the time of this work, no human SLC structures were available, but a crystal structure of SLC39A4 has since been solved (25). The prokaryotic BbZIP (14% sequence identity) crystal structure PDB ID 5TSB (22, 23) was utilized as the template. Because of the low sequence identity, the sequence correspondence was determined manually utilizing information from an alignment of SLC39A4 and BbZIP (22) and functionally important amino acids that are highly conserved across the SLC family (alignment provided in Supplemental Figure 2). The sequence identity between SLC39A4 and SLC39A8 is 31%. The crystal structure was first prepared using the Protein Preparation Wizard within Maestro (79), including adding hydrogens, filling in missing side chains, optimizing hydrogen bonds, and using a restrained minimization of all protein atoms. Upon completion of the model-building calculations, the final model was optimized, and energy was minimized with a truncated-Newton energy minimization using the OPLS (optimized potentials for liquid simulations) 2000 all-atom force field (82).

The resulting models were robust; tertiary and secondary structures were maintained with small differences in flexible loop areas. Furthermore, the RMSD between the inward-facing model and 5TSB was found to be 0.57 Å (Supplemental Figure 4). Residues not resolved in the BbZIP structure were not modeled because it is very difficult to accurately predict the structure of these residues de novo. To assess the structural quality of the model, protein reports were generated within Maestro to examine steric clashes and bond length deviations, etc. Based on this assessment, the models appeared to be of good structural quality and were suitable for structure-based application.

#### Pocket evaluation and identification.

SiteMap (37, 38), Site Finder (36), and FTMap (39) were used to assess the hSLC39A8 homology model and to identify hot spots and pockets amenable for small-molecule binding. The computational tools utilized here complement one another as they have different underlying methodologies. SiteMap is an energy-based method that identifies potential binding sites through detecting, characterizing (hydrophobic and hydrophilic potentials), and evaluating the cavities (volume, hydrophobicity, hydrophilicity, H-bond donor/acceptor, etc.). Site Finder is a geometry-based method, using α spheres (hydrophobic or hydrophilic) to probe regions of atom packing on the protein as potential binding sites. Additionally, FTMap was utilized to detect hot spots, areas with a high PLB, on the protein surface of both models. Default parameters were employed for all 3 tools. Protonate 3D (83) was used before running Site Finder.

#### IFD.

The binding mode for EFV was predicted utilizing the IFD module in the Schrödinger suite of programs using default paraments. The grid center was determined by centroid of Cys326. EFV was prepared using the LigPrep module (84) in the Schrödinger suite. Compared with static docking modules, IFD provides flexibility of the protein binding site to conform to the shape and binding mode of the ligand.

#### MD simulations.

Three replicates of MD simulations were performed, starting from the pose from IFD, using the Desmond (85) module of the Schrödinger suite. The protein-ligand complex was placed in an orthorhombic box with a buffer region of 10 Å. The OPLS4 force field (82) was used in combination with the TIP3P explicit solvent model. Counter ions were added along with 0.15 M salt (NaCl) to neutralize the system and provide physiological conditions. The system was minimized and equilibrated using the default settings. Other parameters not mentioned here follow the default settings in the Desmond module.

Two loops (residues 88–173 and 284–307) in the homology model were missing, so harmonic restraints were applied to C atoms between Met87 and Thr174, and between Met283 and Gln308 to ensure the protein did not fall apart in simulations. The force constant was 100 kcal/mol/Å^2^, and the equilibrium distances were 16.16 and 12.08 Å for Met87–Thr174 and Met283–Gln308, respectively. These restraints were applied throughout simulations (including both equilibration and production phases).

The simulation was designed into 2 stages. First, to allow the protein and ligand time to stabilize, we restrained the ligand in the binding pocket for 500 ns. To do that, a harmonic restraint (100 kcal/mol/Å^2^) between the center of mass of the protein binding site and the ligand was applied. All atoms of the protein binding site (defined as any atoms within 5 Å from the ligand; default in Maestro) and the ligand were selected as 2 groups of the harmonic restraint. The second stage simulations (200 ns/replicate) started from the structure extracted from the last frame of the 500 ns simulations, and the center of mass harmonic restraints was removed.

### Statistics

Statistical analyses were performed using 1-way ANOVA with Dunnett’s or Tukey’s multiple-comparison test, as indicated. *P* values < 0.05 were considered significant.

### Study approval

Animals were cared for in accordance with the National Research Council’s Guide for the Care and Use of Laboratory Animals at Massachusetts General Hospital (MGH) in an AAALAC-accredited facility. All animal procedures were conducted in accordance with protocol 2003N000158, approved by the MGH Institutional Animal Care and Use Committee.

### Data availability

All data supporting the findings of this study are available within the article, its supplemental information, and the Supporting Data Values file.

## Author contributions

KLDG, ADW, JP, JDV, JET, DBG, and RJX conceived the study. KLDG, ADW, CM, MT, EVMM, and TM designed methods. KLDG, ADW, CM, MT, BC, and AMP performed analysis. KLDG, ADW, CM, YG, EVMM, BC, and AMP conducted investigations. KLDG, ADW, and CM performed validation. KLDG, ADW, CM, MT, and YG visualized data. KLDG, ADW, JP, CM, MT, YG, EVMM, and DBG wrote the original draft. KLDG, ADW, JP, CM, MT, YG, EVMM, and DBG reviewed and edited the article. DBG and RJX acquired funding. TM, JDV, JP, JET, DBG, and RJX supervised the study.

## Funding support

This work is the result of NIH funding, in whole or in part, and is subject to the NIH Public Access Policy. Through acceptance of this federal funding, the NIH has been given a right to make the work publicly available in PubMed Central.

- The Leona M. and Harry B. Helmsley Charitable Trust (to RJX, DBG).
- NIH P30 DK043351 (to RJX).

## Supplementary Material

Supplemental data

Supporting data values

## Figures and Tables

**Table 1:**
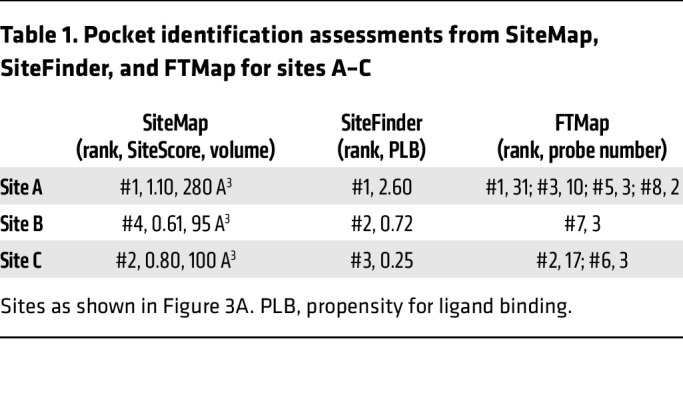
Pocket identification assessments from SiteMap, SiteFinder, and FTMap for sites A–C

## Version 1 11/03/2025

Electronic publication
