# Supplementary material for: Allosteric modulation of the solute carrier transporter SLC39A8 potentiates manganese and cadmium uptake
Source: J Clin Invest. 2025 Nov 3;135(21):e191096. doi: 10.1172/JCI191096 (PMC12578388; doi:10.1172/JCI191096)
Supplement: Supplemental data [file jci-135-191096-s280.pdf]

Allosteric modulation of solute carrier transporter SLC39A8 potentiates manganese and cadmium uptake

Kelly L. Damm-Ganamet[1], Clara Moon[2], Alan D. Wickenden[1], Mark Tichenor[1], Yunhui Ge[1], Eduardo V. Mercado-Marin[1], Brian Chiou[1], Ayla Manughian-Peter[2], Taraneh Mirzadegan[1], Jennifer D. Venable[1], Ramnik J. Xavier[3,4], Jennifer E. Towne[2], Daniel B. Graham[3,4,*], Jacqueline Perrigoue[5,*]

[1]Therapeutics Discovery, Johnson & Johnson, 3210 Merryfield Row, San Diego, CA, 92121, USA

[2]Discovery Immunology, Johnson & Johnson, 3210 Merryfield Row, San Diego, CA, 92121, USA

[3]Broad Institute of MIT and Harvard, Cambridge, MA, 02142, USA

[4]Center for Computational and Integrative Biology, Department of Molecular Biology, Massachusetts General Hospital, Harvard Medical School, Boston, MA, 02114, USA

[5]Discovery Immunology, Johnson & Johnson, 1400 McKean Rd, Spring House, PA, 19477, USA

**Corresponding authors: Jacqueline Perrigoue (jperrigo@its.jnj.com) and Daniel B. Graham (dgraham@broadinstitue.org)*

Supplemental Information contains:

Supplemental Figures 1-9

Supplemental Table 1

Supplemental materials and Methods including chemistry, plasmid constructs, and cell culture reagents

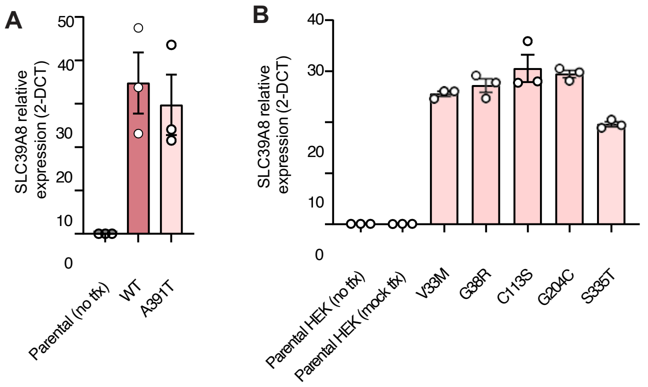

Supplemental Figure 1. *SLC39A8* gene expression in HEK293T cells after transient transfection. HEK293T cells were transiently transfected to express WT or CDG mutant *SLC39A8*. **A.** For parental HEK293T as well as WT and A391T transfected cells, *SLC39A8* mRNA expression was quantified using SYBR chemistry with custom designed primers as codon-optimized constructs were utilized. Data were normalized to the housekeeping gene *GAPDH.* **B.** *SLC39A8* mRNA expression was quantified using Taqman chemistry with commercially available probes for parental HEK293T cells as well as V33M, G38R, C113S, G204C, and S335T variant transfected cells. Data were normalized to GAPDH. No tfx = no transfection; mock tfx = mock transfection.

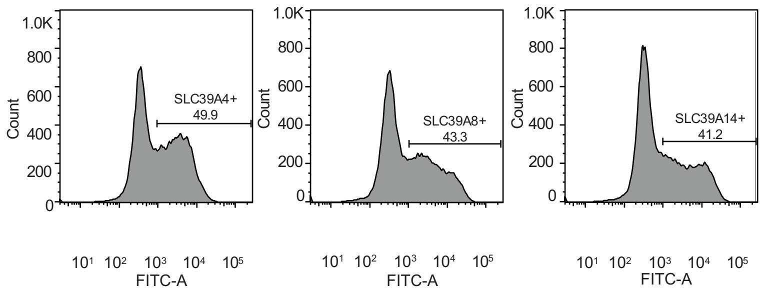

Supplemental Figure 2. Functional expression of SLC39A4, SLC39A8, and SLC39A14 by fluozin-3 uptake by flow cytometry. HEK293T cells were transiently transfected to express SLC39A4, SLC39A8, or SLV39A14. Intracellular zinc was measured using FluoZin-3 cell permeant Zn indicator dye to assess zinc uptake by these transporters.

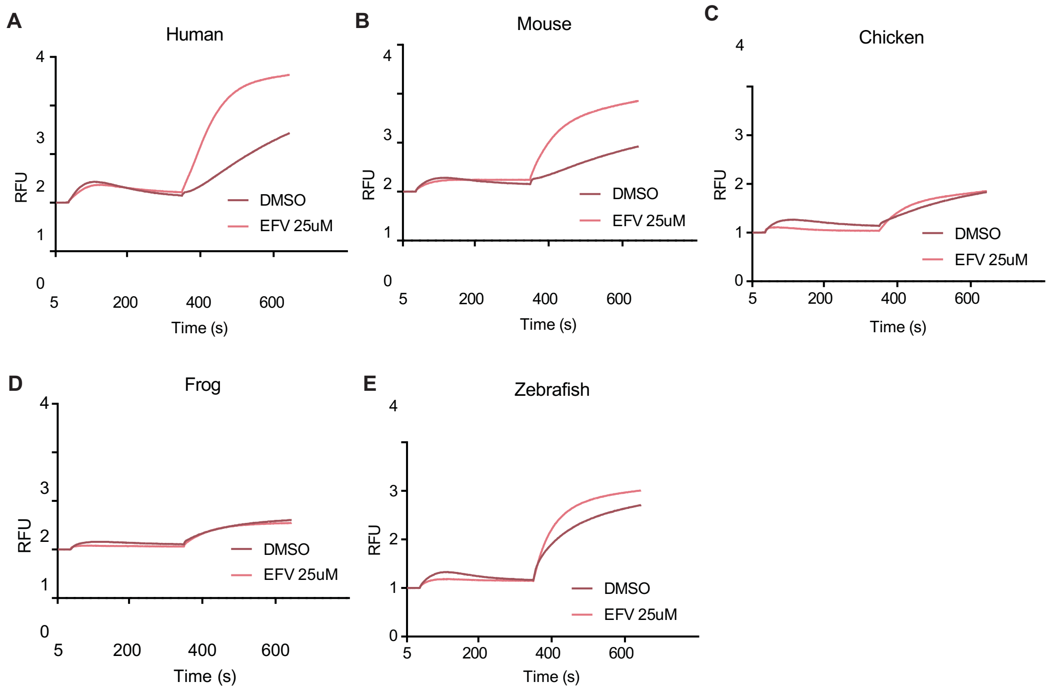

Supplemental Figure 3. Efavirenz potentiates human and mouse SLC39A8. A-E. Efavirenz potentiates human and mouse SLC39A8 (**A-B**), but not chicken (**C**), frog (**D**), or zebrafish (**E**).

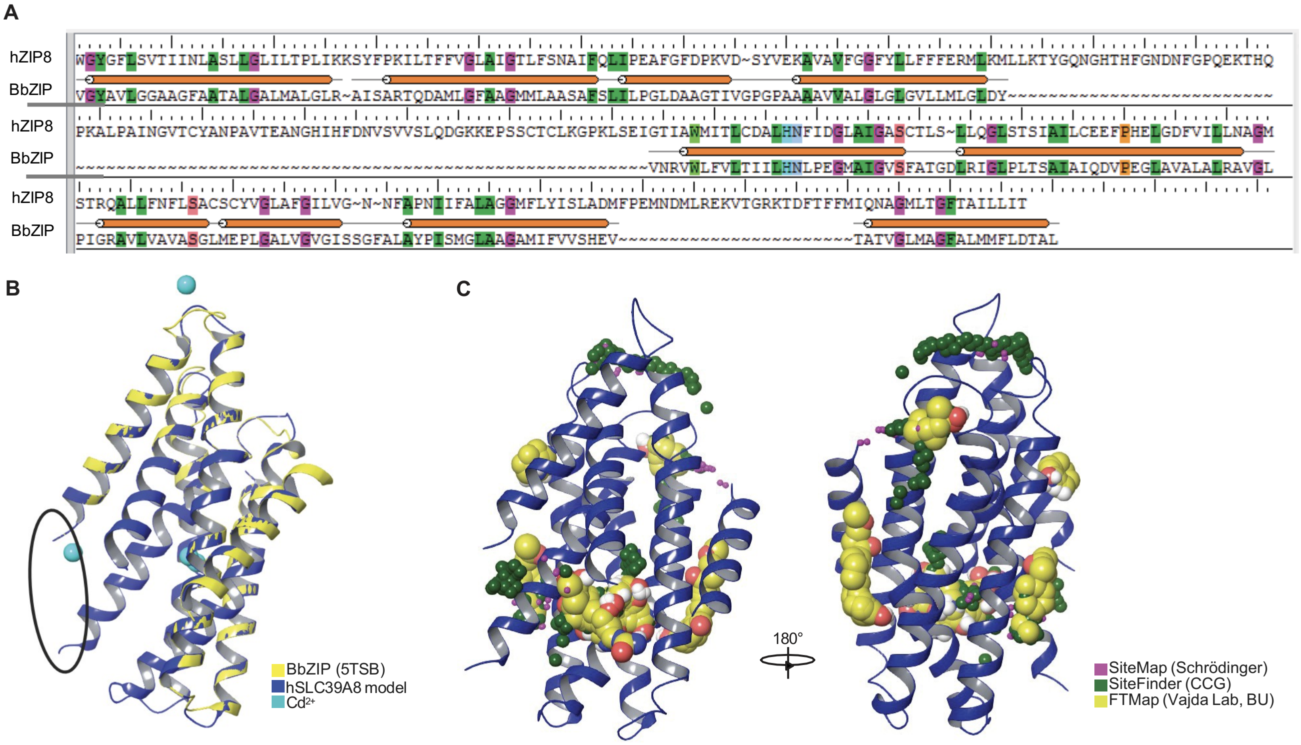

Supplemental Figure 4. Homology model of hSLC39A8 TMD. A. Residue correspondence between hSLC39A8 and BbZIP8 in TMD. PDB ID 5TSB used at template structure. **B.** Resulting homology model of hSLC39A8 TMD (blue) overlaid with BbZIP TMD (yellow). The overall tertiary structure is maintained with major differences in flexible loop regions of TM Secondary structure helices maintained. Break in model at location of hSLC39A8 insertion highlighted. Cd2 + ions shown in cyan. **C.** Six sites identified on homology model of hSLC39A8 as potential binding pocket for Efavirenz. SiteMap spheres are shown in green, SiteFinder in pink and in yellow.

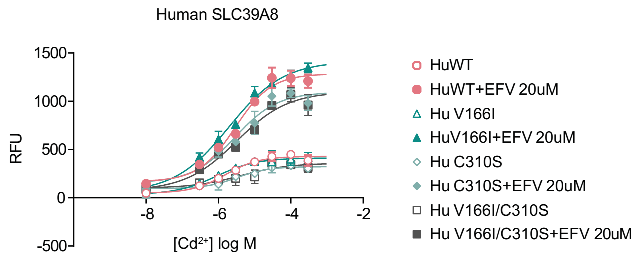

Supplemental Figure 5. Mutagenesis data reveals mutations in putative metal exit pathway does not alter EFV sensitivity. SLC39A8 mutations V166I, C301S, and V166I/C301S do not alter EFV sensitivity by cadmium uptake. Hu = human; EFV = efavirenz; WT = wild type; RFU = relative fluorescence units.

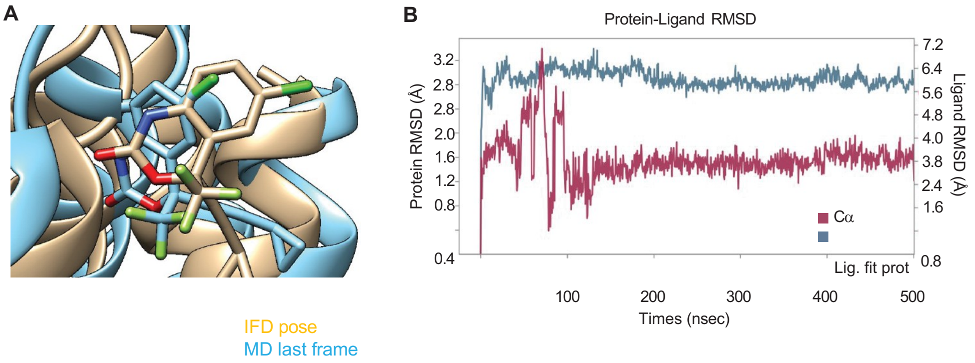

C

	SiteMap (Rank, SiteScore, Volume)	SiteFinder (Rank, PLB)
Site B (homology model)	#4, 0.61, 95 A^3	#2, 0.72
Site B (IFD conformation)	#4, 0.62, 95 A^3	#2, 1.32
Site B (MD snapshot)	#1, 1.28, 107 A^3	#2, 2.01

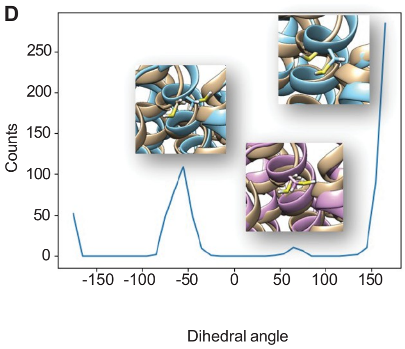

Supplemental Figure 6. Representative molecular dynamics stimulation of EFV in complex with hSLC39A8. Induced fit docking pose used as starting coordinates. **A.** Overlay of IFD pose with last frame of MD simulation; RMSD is 3.127 Ang. **B.** Protein and ligand RMSD across 500 ns MD trajectory. **C.** Pocket identification assessments from SiteMap, SiteFinder and FTMap for Sites A-C. SiteMap and SiteFinder ranking and scores improve after conformational sampling. **D.** Cys326 turns away from pocket in majority simulation.

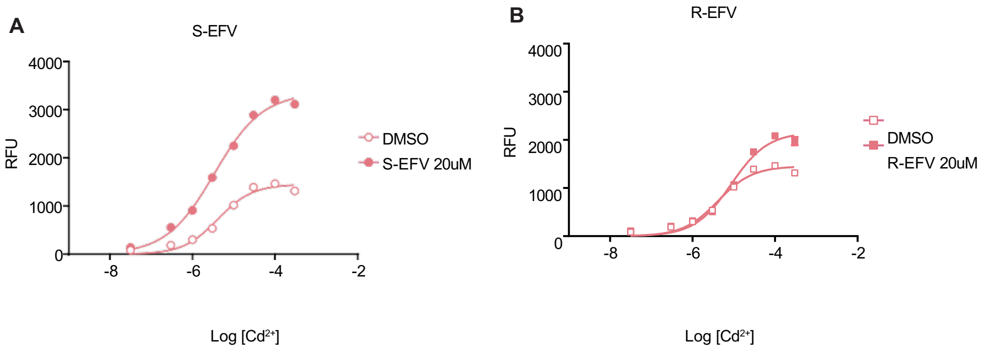

Supplemental Figure 7. Potentiation of SLC39A8 by R-EFV and S-EFV. R-EFV is less effective at potentiating SLC39A8, showing a 70% reduction in Emax compared to S-EFV. EFV = efavirenz; RFU = relative fluorescence units.

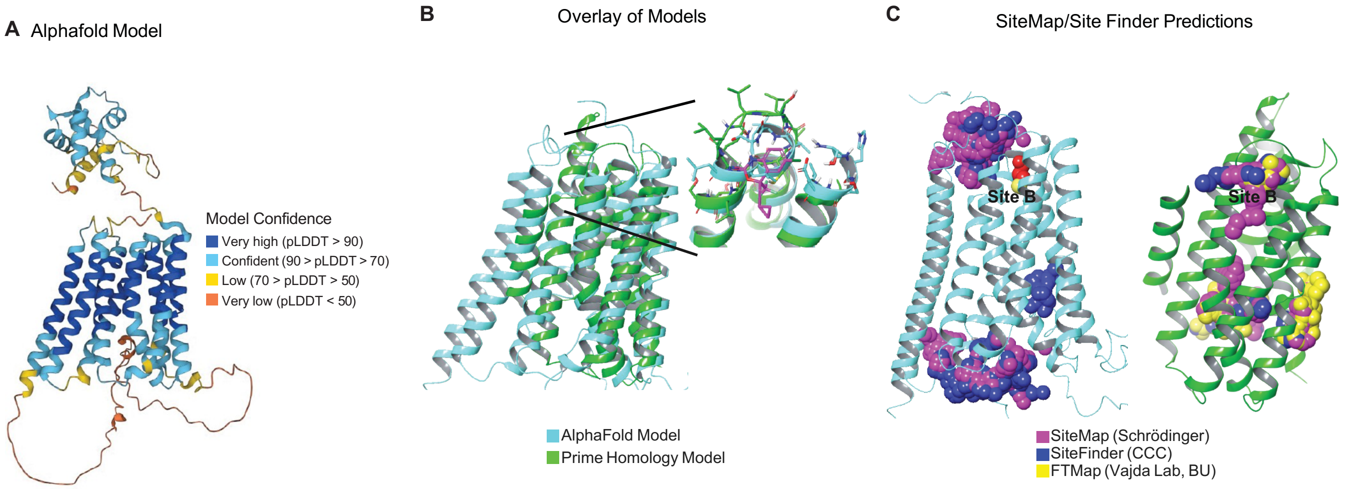

Supplemental Figure 8. Comparison of Prime homology model to AlphaFold prediction. A. AlphaFold model. **B.** Overlay of Prime (green) and AlphaFold (cyan) models. RMSD = 3.31 (Å). Site B is shown zoomed in. **C.** SiteMap and SiteFinder predictions for Prime (green) and AlphaFold (cyan) models. Site B is highlighted on both models and Cys326 is shown in red spheres on the AlphaFold model to demonstrate where the pocket would be located had it been identified by SiteMap or SiteFinder. SiteMap spheres are shown in pink, SiteFinder in blue and FTMap probes in yellow.

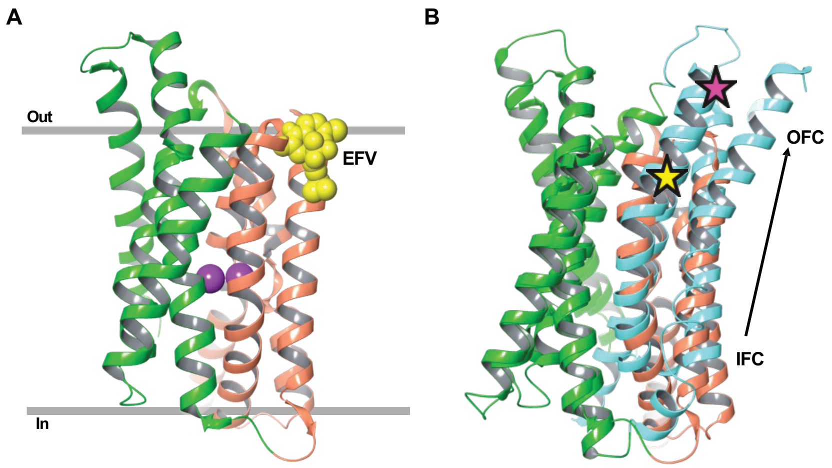

Supplemental Figure 9. Mapping of putative EFV binding site onto the transport domain for both inward- facing and outward-facing conformations. A. ZIP proteins function with a two-domain structure, comprising a static scaffold domain (green) and a transport domain (pink), shown here in the inward-facing conformation (IFC). The putative EFV (shown in yellow sphere representation) binding site is located in the transport domain, facing away from the scaffold interface. Metal ions (shown in purple sphere representation) occupy the putative metal binding site. **B.** The scaffold domain remains static (green) between the inward-facing (IFC) and outward-facing (OFC) conformations whereas the transport domain (IFC pink; OFC cyan) moves vertically (highlighted with arrow) to facilitate the transfer of zinc ions (purple) across the membrane. The putative EFV binding pocket location is highlighted on the IFC (yellow star) and OFC (fuchsia star). The OFC was modeled using the ZIP13 AlphaFold prediction.

Supplemental Table 1: Summary of cadmium uptake experiments.

Cadmium uptake experiments

n=28	Control	Efavirenz (20uM)	P-value
Cd EC50 (uM)	3.4 +/- 0.29	4.5 +/- 0.39	0.0014
Slope	1.2 +/- 0.06	0.99 +/- 0.04	0.0012
Emax	607 +/- 71	1710 +/- 187	<0.0001

Supplementary Information: Chemistry Methods

General materials and methods for chemical synthesis

All solvents and chemicals were used as purchased without further purification. Nuclear magnetic resonance spectra were obtained on Bruker model DRX spectrometers. Chemical shifts (δ) are expressed in parts per million, relative to internal standard tetramethylsilane; coupling constants (J) are in Hz. The following abbreviations are used to describe peak patterns when appropriate: s (singlet), d (doublet), t (triplet), m (multiplet). HPLC−MS chromatograms and spectra were obtained using one of the following methods: (1) Agilent 1200 HPLC and G6100 system on X-Bridge ShieldRP18 (50 × 2.1 mm, 5 μm) and a gradient system of 0.05% NH_4OH in H_2O/CH_3CN, 100:0 to 5:95 over 7.5 min, then 100:0 for 2.5 min at a temperature of 40°C; (2) Agilent 1200 HPLC and G6100 system on Phenomenex Luna-C18 (50 × 2 mm, 5 μm) and a gradient system of 0.1% TFA in H_2O/0.05% TFA in CH_3CN, 100:0 to 15:85 over 7.5 min, then 100:0 for 2.5 min at a temperature of 50°C; or (3) Agilent 1100 HPLC and G1367A system on X-Bridge C18 (100 × 3 mm, 3.5 μM) and a gradient system of 20 mM NH_4OH in H_2O/CH_3CN 90:10 over 2 min, then 0:100 for 1 min at a flow rate of 2.4 mL/min at a temperature of 45°C. All compounds tested were a minimum of 95% purity as determined by HPLC.

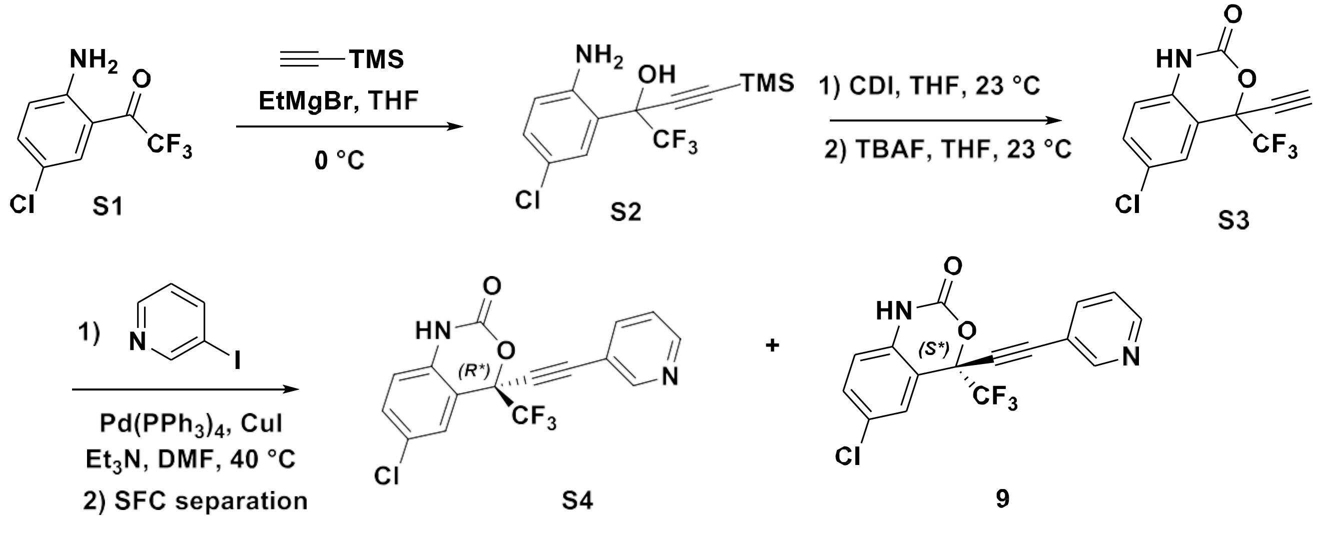

S2: 2-(2-amino-5-chlorophenyl)-1,1,1-trifluoro-4-(trimethylsilyl)but-3-yn-2-ol

To a solution of trimethylsilylacetylene (4400 mg, 44.8 mmol) in THF (45 mL) was cooled to 0 °C and then ethylmagnesium bromide (3 M in diethylether, 15 mL, 44.8 mmol) was added slowly and the resulting solution was stirred at 0 °C for 1 hour and then warmed to 23 °C for 1 hour. The mixture was then cooled to 0 °C and 1-(2-amino-5-chlorophenyl)-2,2,2-trifluoroethanone (5000 mg, 23.4 mmol, **S1**) was added portion wise over 5 minutes. The reaction was stirred at 0 °C for 1 hour and then warmed to 23 °C and stirred for 2 hours. The reaction was then quenched by the addition of sat. aq. NH_4Cl (40 mL). The layers were then separated and the aqueous was extracted with EtOAc (3 x 30 mL). The combined organics were dried with $MgSO_4$, filtered, and concentrated in vacuo. The crude material was purified by silica gel chromatography using 0-100% EtOAc in hexanes to give the title compound (6.16 g, 85% yield) as a white solid. ^{1}H NMR (600 MHz, DMSO) δ 8.22 (s, 1H), 7.39 (d, J = 2.5 Hz, 1H), 7.11 (dd, J = 8.7, 2.6 Hz, 1H), 6.68 (d, J = 8.7 Hz, 1H), 5.64 (s, 2H), 0.24 (s, 9H). LCMS (ESI) mass calcd for $C_{13}H_{15}ClF_3NOSi$, 321.06; m/z found; 322.1 $[M+H]^+$.

S3: 6-chloro-4-ethynyl-4-(trifluoromethyl)-1,4-dihydro-2H-benzo[d][1,3]oxazin-2-one

To a solution of 2-(2-amino-5-chlorophenyl)-1,1,1-trifluoro-4-(trimethylsilyl)but-3-yn-2-ol (**S2**) (6.00 g, 18.6 mmol) in THF (150 mL) was added CDI (7.56 g, 46.6 mmol) and the resulting mixture was then heated to 55 °C for 16 hours. The mixture was concentrated in vacuo then diluted with water (150 mL), sat. aq. $NaHCO_3$ (100 mL) and EtOAc (100 mL). The layers were separated and the aqueous extracted with EtOAc (2 x 100 mL). The combined organics were washed with 0.4 N HCl (2 x 50 mL), followed by 10 wt% brine (1 x 50 mL) then dried with $MgSO_4$, filtered

and to the organic layer was concentrated in vacuo. The crude material was diluted in THF (150 mL) and cooled to 0 °C and then TBAF (1.0 M in THF, 18.4 mL, 18.4 mmol) was added. After 1 hour, the mixture was warmed to 23 °C and then concentrated in vacuo. The crude material was dissolved in EtOAc (150 mL) and washed with 13 wt% NaCl (2 x 50 mL), then the organics were dried with $MgSO_4$, filtered, and concentrated in vacuo. The crude material was purified by silica gel chromatography using 0-60% EtOAc in hexanes to give the title compound (4.20 g, 83 % yield) as a white powder. ^1H NMR (600 MHz, DMSO) δ 11.18 (s, 1H), 7.60 (dd, J = 8.6, 2.3 Hz, 1H), 7.48 (d, J = 2.3 Hz, 1H), 7.03 (d, J = 8.6 Hz, 1H), 4.61 (s, 1H). LCMS (ESI) mass calcd for $C_{11}H_5ClF_3NO_2$, 274.9; m/z found; 276.0 $[M+H]^+$.

Synthesis of compound S4 and 9: (R)-6-chloro-4-(pyridin-3-ylethynyl)-4-(trifluoromethyl)-1,4-dihydro-2H-benzo[d][1,3]oxazin-2-one and (S)-6-chloro-4-(pyridin-3-ylethynyl)-4-(trifluoromethyl)-1,4-dihydro-2H-benzo[d][1,3]oxazin-2-one.

To a solution of 6-chloro-4-ethynyl-4-(trifluoromethyl)-1,4-dihydro-2H-benzo[d][1,3]oxazin-2-one (**S3**) (3.80 g, 13.8 mmol), 3-iodopyridine (3.10 g, 15.2 mmol), triethylamine (5.3 mL, 38.4 mmol), $Pd(PPh_3)_4$ (796 mg, 0.69 mmol) and CuI (131 mg, 0.69 mmol) in DMF (40 mL) was evacuated and backfilled with inert N_2 (3x) and then the mixture was stirred at 40 °C for 4 hours. The mixture was cooled to 23 °C and diluted with water (120 mL) and EtOAc (40 mL). The layers were separated and the aqueous extracted with EtOAc (2 x 40 mL). The combined organics were dried with $MgSO_4$, filtered, and concentrated in vacuo. The crude material was purified by silica gel chromatography using 0-50% EtOAc in hexanes to give racemate (3.56 g, 73% yield) as a pale-yellow solid. The racemate was purified by chiral SFC (Stationary phase: Chiralpak AD-H 5μm

250 x 30 mm, Mobile phase: 15% methanol: isopropanol (1:1) with 0.2% isopropylamine, 85% CO_2) to give compound **S4** (1.15 g) and compound **9** (1.30 g) as the first and second eluting peaks, respectively. The stereochemistry was tentatively assigned S for the active enantiomer, corresponding to the active S-enantiomer of EFV. ^{1}H NMR and LCMS were identical for both compounds: ^{1}H NMR (600 MHz, DMSO) δ 11.21 (s, 1H), 8.87 (s, 1H), 8.70 (d, J = 4.3 Hz, 1H), 8.13 (dt, J = 7.9, 1.9 Hz, 1H), 7.73 (d, J = 2.3 Hz, 1H), 7.61 (dd, J = 8.6, 2.3 Hz, 1H), 7.52 (ddd, J = 7.9, 4.9, 0.8 Hz, 1H), 7.06 (d, J = 8.6 Hz, 1H). LCMS (ESI) mass calcd for $C_{16}H_8ClF_3N_2O_2$, 352.0; m/z found; 353.0 $[M+H]^+$.

Abbreviations: TMS – trimethylsilyl, CDI – 1,1'-carbonyldiimidazole, TBAF – tetrabutylammonium fluoride, THF – tetrahydrofuran, DMF - *N,N*-dimethylformamide

Supplementary Information: qPCR methods for supplemental Figure S1

RNA Extraction

Frozen cell pellets were placed on ice and lysed in Qiagen RLT Plus Lysis Buffer (Qiagen 1053393) containing 0.01% β-mercaptoethanol (Sigma-Aldrich 444203). Cell pellets were homogenized using a p1000 pipet. The RNA extraction was performed according to the manufacturer's instructions using the RNeasy Miniprep Plus Kit (Qiagen 95134). Samples were eluted in 35 µl nuclease free water, and concentrations were measured by Nanodrop.

qRT-PCR: Taqman Chemistry

cDNA synthesis and qPCR were performed in a single reaction using the qScript XLT One-Step RT-qPCR ToughMix (QuantaBio 95134) and Taqman primer-probe sets (ThermoFisher 431182), using 20 ng RNA for each reaction. The reaction was performed in a total volume of 20 µl as follows: step 1) 15 min at 50°C; step 2) 1 min 95°C; step 3) 15 sec at 95°C; step 4) 1 min at 60°C; repeat steps 3-4 for 40 cycles. The resulting data was analyzed using the Applied Biosystems Expression Suite Software. Data were normalized to housekeeping GAPDH. Taqman probe information can be found in **Supplemental Table S2**.

qRT-PCR: SYBR Chemistry

100 ng of RNA was used for each sample to perform cDNA synthesis using the High-Capacity cDNA Reverse Transcription kit (Thermo 4374966). This reaction was performed in a total volume of 20 µl. The cDNA synthesis PCR cycle was as follows: step 1) 10 min at 25°C; step 2) 120 min at 37°C; step 3) 5 min at 85°C; step 4) hold at 4°C.

The resulting cDNA was used for qPCR. The reaction was performed using SsoAdvanced Universal SYBR Green Supermix (BioRad 1725272) and custom primers from Integrated DNA Technologies as follows: step 1) 30 sec at 95°C; step 2) 15 sec at 95°C; step 3) 30 sec at 60°C; repeat steps 2-3 for 40 cycles; step 4) 15 sec at 95°C, followed by continuous melt curve from 60°C -95°C, 0.05°C/s. The resulting data was analyzed using the Applied Biosystems Expression Suite Software. Data were normalized to housekeeping GAPDH. Primer sequence information can be found in **Supplemental Table 2**.

Supplemental Table 2. **SLC39A8 detection reagents**

Gene name	Taqman Probe ID	Forward Primer (SYBR)	Reverse primer (SYBR)
SLC39A8	Hs00223357_m1	CAACACCTGCTGGAACAGA	GAACTTGCTGCTGGTGATCT
GAPDH	Hs99999905_m1	GGAGCGAGATCCCTCCAAAAT	GGCTGTTGTCATACTTCTCATGG

Supplementary Information: Plasmid Construct

1. SLC39A2 (Human)

Gene name: SLC39A2, Length: 945 bp
Sequence:
GGTACCACCATGGAGCAGCTGCTGGGCATCAAGCTGGGCTGCCTGTTTGCACTGCTGGCCCTGACCCTGGGATGC
GGACTGACACCTATCTGTTTTAAGTGGTTCCAGATCGACGCCGCCAGAGGCCACCACAGACTGGTGCTGAGGCTG
CTGGGCTGTATCAGCGCCGGCGTGTTTCTGGGAGCAGGCTTCATGCACATGACCGCAGAGGCCCTGGAGGAGATC
GAGTCCCAGATCCAGAAGTTCATGGTGCAGAACCGCTCCGCCTCTGAGCGGAATAGCTCCGGCGACGCAGATAGC
GCCCACATGGAGTACCCTTATGGCGAGCTGATCATCTCTCTGGGCTTCTTTTTCGTGTTTTTCCTGGAGAGCCTGGC
CCTGCAGTGCTGTCCAGGCGCAGCAGGCGGCTCCACAGTGCAGGATGAGGAGTGGGGAGGCGCCCACATCTTTG
AGCTGCACTCTCACGGCCACCTGCCAAGCCCTTCCAAGGGACCACTGCGGGCCCTGGTGCTGCTGCTGTCTCTGAG
CTTTCACAGCGTGTTCGAGGGCCTGGCAGTGGGACTGCAGCCTACCGTGGCAGCAACAGTGCAGCTGTGCCTGGC
CGTGCTGGCACACAAGGGACTGGTGGTGTTTGGCGTGGGCATGAGACTGGTGCACCTGGGCACCTCTAGCAGGT
GGGCCGTGTTCTCCATCCTGCTGCTGGCCCTGATGTCTCCACTGGGCCTGGCCGTGGGCCTGGCCGTGACAGGCG
GCGACTCCGAGGGCGGCAGAGGACTGGCACAGGCCGTGCTGGAGGGCGTGGCCGCCGGCACCTTTCTGTACGTG
ACATTCCTGGAGATCCTGCCCAGAGAGCTGGCAAGCCCAGAGGCACCACTGGCAAAGTGGTCCTGCGTGGCCGCC
GGCTTTGCCTTCATGGCCTTCATCGCCCTGTGGGCCTAATCTAGA

1. SLC39A4 (Human)

Gene name: SLC39A4, Length: 1959 bp
Sequence:
GGTACCACCATGGCCTCTCTGGTGAGCCTGGAGCTGGGACTGCTGCTGGCCGTGCTGGTGGTGACCGCAACAGCA
AGCCCACCTGCAGGACTGCTGTCTCTGCTGACAAGCGGACAGGGCGCCCTGGATCAGGAGGCCCTGGGAGGACT
GCTGAACACCCTGGCAGACAGGGTGCACTGCGCAAATGGACCCTGCGGCAAGTGTCTGTCTGTGGAGGATGCCCT
GGGCCTGGGCGAGCCAGAGGGCAGCGGACTGCCACCCGGCCCTGTGCTGGAGGCAAGATACGTGGCAAGGCTG
AGCGCCGCCGCCGTGCTGTATCTGAGCAACCCTGAGGGCACCTGTGAGGATGCCAGAGCCGGACTGTGGGCATC
CCACGCAGACCACCTGCTGGCCCTGCTGGAGTCCCCAAAGGCCCTGACACCCGGCCTGTCTTGGCTGCTGCAGAG
GATGCAGGCCAGAGCCGCAGGACAGACCCCAAAGATGGCATGCGTGGACATCCCCCAGCTGCTGGAGGAGGCCG
TGGGAGCAGGCGCCCCAGGCAGCGCCGGAGGCGTGCTGGCCGCCCTGCTGGATCACGTGCGGTCTGGCAGCTGT
TTCCACGCCCTGCCATCCCCCCAGTACTTTGTGGACTTCGTGTTTCAGCAGCACAGCTCCGAGGTGCCCATGACCCT
GGCAGAGCTGAGCGCCCTGATGCAGAGGCTGGGAGTGGGAAGGGAGGCACACTCCGATCACTCTCACAGGCACA
GGGGAGCCTCTAGCCGGGACCCTGTGCCACTGATCTCCTCTAGCAATTCCTCTAGCGTGTGGGATACAGTGTGCCT
GTCCGCCAGAGACGTGATGGCAGCCTATGGCCTGTCTGAGCAGGCAGGAGTGACCCCTGAGGCCTGGGCCCAGC
TGAGCCCAGCCCTGCTGCAGCAGCAGCTGTCCGGAGCATGTACATCCCAGTCTAGGCCTCCAGTGCAGGATCAGC
TGAGCCAGTCCGAGAGATACCTGTATGGCTCTCTGGCCACCCTGCTGATCTGCCTGTGCGCCGTGTTCGGACTGCT
GCTGCTGACCTGCACAGGCTGTAGAGGCGTGACCCACTACATCCTGCAGACATTTCTGTCCCTGGCAGTGGGAGC
AGTGACCGGCGACGCCGTGCTGCACCTGACACCCAAGGTGCTGGGACTGCACACCCACAGCGAGGAGGGACTGT
CCCCACAGCCTACATGGAGGCTGCTGGCAATGCTGGCAGGACTGTATGCCTTCTTTCTGTTCGAGAACCTGTTTAA
TCTGCTGCTGCCCCGCGACCCTGAGGATCTGGAGGACGGCCCTTGCGGACACTCCTCTCACTCCCACGGCGGACAC
TCCCACGGCGTGTCTCTGCAGCTGGCACCTTCTGAGCTGAGGCAGCCAAAGCCACCTCACGAGGGCAGCCGGGCC
GATCTGGTGGCCGAGGAGAGCCCAGAGCTGCTGAACCCAGAGCCACGGAGACTGAGCCCAGAGCTGAGGCTGCT
GCCTTACATGATCACCCTGGGCGATGCCGTGCACAATTTCGCAGACGGACTGGCAGTGGGAGCAGCCTTTGCCAG
CTCCTGGAAGACCGGACTGGCCACATCCCTGGCCGTGTTCTGCCACGAGCTGCCACACGAGCTGGGCGACTTTGC
CGCCCTGCTGCACGCAGGACTGAGCGTGCGCCAGGCCCTGCTGCTGAACCTGGCATCCGCCCTGACCGCCTTCGCC
GGCCTGTACGTGGCCCTGGCCGTGGGCGTGAGCGAGGAGTCCGAGGCCTGGATTCTGGCCGTGGCCACAGGCCT
GTTCCTGTATGTGGCCCTGTGCGATATGCTGCCTGCCATGCTGAAGGTGCGGGACCCTAGACCCTGGCTGCTGTTT

CTGCTGCACAATGTGGGACTGCTGGGAGGATGGACAGTGCTGCTGCTGCTGAGCCTGTATGAGGACGACATCACC
TTCTAATCTAGA

2. SLC39A14 (Human)

Gene name: SLC39A14, Length: 1494 bp
Sequence:
GGTACCACCATGAAGCTGCTGCTGCTGCACCCTGCCTTTCAGAGCTGTCTGCTGCTGACCCTGCTGGGACTGTGGA
GGACCACACCAGAGGCACACGCCAGCTCCCTGGGCGCCCCTGCCATCAGCGCCGCCTCCTTCCTGCAGGACCTGAT
CCACAGATACGGCGAGGGCGATAGCCTGACCCTGCAGCAGCTGAAGGCCCTGCTGAATCACCTGGACGTGGGAG
TGGGAAGGGGAAACGTGACACAGCACGTGCAGGGCCACAGAAATCTGTCCACCTGCTTTTCTAGCGGCGATCTGT
TTACAGCCCACAACTTCTCTGAGCAGAGCAGGATCGGCTCCTCTGAGCTGCAGGAGTTCTGTCCAACCATCCTGCA
GCAGCTGGACTCCAGGGCCTGCACATCTGAGAACCAGGAGAATGAGGAGAACGAGCAGACCGAGGAGGGCCGC
CCATCTGCCGTGGAAGTGTGGGGATACGGACTGCTGTGCGTGACAGTGATCTCCCTGTGCTCTCTGCTGGGAGCA
TCCGTGGTGCCCTTTATGAAGAAGACCTTCTACAAGCGCCTGCTGCTGTACTTCATCGCCCTGGCCATCGGCACACT
GTATTCCAACGCCCTGTTCCAGCTGATCCCCGAGGCCTTTGGCTTCAATCCTCTGGAGGACTACTACGTGAGCAAG
AGCGCCGTGGTGTTTGGCGGCTTCTACCTGTTCTTTTTCACAGAGAAGATCCTGAAGATCCTGCTGAAGCAGAAGA
ACGAGCACCACCACGGCCACAGCCACTATGCCTCCGAGTCTCTGCCCTCCAAGAAGGATCAGGAGGAGGGCGTGA
TGGAGAAGCTGCAGAATGGCGACCTGGATCACATGATCCCTCAGCACTGTAGCTCCGAGCTGGACGGCAAGGCCC
CAATGGTGGATGAGAAAGTGATCGTGGGCAGCCTGTCCGTGCAGGACCTGCAGGCCTCTCAGAGCGCCTGCTACT
GGCTGAAGGGCGTGCGGTATTCCGACATCGGCACCCTGGCCTGGATGATCACACTGTCTGACGGCCTGCACAACT
TCATCGATGGCCTGGCCATCGGCGCCTCTTTTACCGTGAGCGTGTTCCAGGGCATCTCCACATCTGTGGCCATCCTG
TGCGAGGAGTTTCCTCACGAGCTGGGCGATTTCGTGATCCTGCTGAACGCCGGCATGTCTATCCAGCAGGCCCTGT
TTTTCAATTTTCTGAGCGCCTGCTGTTGCTACCTGGGACTGGCCTTCGGCATCCTGGCAGGCAGCCACTTTTCCGCC
AATTGGATCTTCGCCCTGGCCGGCGGCATGTTTCTGTATATCAGCCTGGCCGACATGTTCCCCGAGATGAACGAGG
TGTGCCAGGAGGATGAGCGGAAGGGCTCCATCCTGATCCCTTTTATCATCCAGAATCTGGGCCTGCTGACCGGCTT
CACAATCATGGTGGTGCTGACCATGTATAGCGGCCAGATCCAGATCGGCTAATCTAG

3. SLC39A8 SPECIES

tr|F1NBL5|F1NBL5_CHICK Uncharacterized protein OS=Gallus gallus GN=SLC39A8 PE=4 SV=2
>CHICKEN
MLSGVAVRGSPAATTFAEDVLRVFGSNRSLSAGQLSALLSQLGAAPALSAVLPLPHLHHN
QCLTGEEIFSLHGIPNNSHISNSDFSIICPAVLQQLIFHPCDHQENSVDASRPHSLQVWG
FGFLAVTIINLASLLGFILTPLLKKSYFPKILTYFVGLAVGTLFSNAIFQLIPEAFGFDS
HVDNYIEKAVAVFGGFYILFFVERILKVILKIYNQPGHNDVENGEENQSKDKTSPSKPPS
SSNGVTCYANSAIVESNGNFGFDSISVVSAQEEAVQGSLCKCLGGRPLSKIGTIAWMVTL
SDAVHNFLDGLAIGASFTLSLFQGLSTSIAILCEEFPHELGDFVILLNAGMSIRQALFFN
FLSACSCYIGMALGILVGNNFAPTIIFAVAGGMFLYISLADMFPEMNDMLREKVTGRKMD
LTFFLIQNAGLLTGFAAILLITLYEGNIQL

>tr|F1R5W1|F1R5W1_DANRE Solute carrier family 39 (zinc transporter), member 8 OS=Danio rerio GN=slc39a8 PE=4 SV=2
>ZF
MEESFLQNVLGFYGEESSLSVGNLELFLQLITSRRAAAVEDEGNPLKSAECLSLSELLSA
FGLSNASVVSVSNLEMMCPAILNQVLIPACPYTSSNLNASASISDHKVWGYGFLAVTVIN
LAALLGLFLVPFTKKKYFPKVLMYFIGLAIGTLFSNAVLQLIPEALGLDPKDDNYVLNVV
GIFGGFYILFVTERILKMVLKTDTELGHSHFPPLQSSDVTISTISNDVVISNISGDIITN

NTNHEQNSISEKSNNPSESPAVEQNACALFACRWLKGSAMSNIKTVAWMITVSDALHNFI
DGLAIGASFTLSLLSGFSTSIAIFCEEFPHELGDFVILLNSGMSVGQAAFFNLLSAMCCY
LGLALGILLGSNFAPNAIFAIAGGMFLYISLADMFPEMNSIMASHTKDYQERVVFFLIQN
AGLLTGFTIILLITLFAGDINLQ

>tr|A0A5G3H9Q7|A0A5G3H9Q7_XENTR Solute carrier family 39 member 8 OS=Xenopus tropicalis OX=8364 GN=slc39a8 PE=4 SV=1
MSFPGYRLFLALYGAVFVILVGANDAGDMFTQDILQLYGHDGRLSSSNVSRLMNGCDARD
ELHNLEHLHYNRCLSTEDIFSIYNIKNDQITNVTFETFCPAILHQIFFHPCTFESQDEST
QRPSPAQVWGFSFLSVTIINLTSLLGLFITPLIKKPYFPKILTYFVGLAIGTLFSNAIFQ
LIPEAFGFDPKVDNYVPKAVAIFGGFYILLFVERLLKLILNIYGEGVHTHLEIDHVPHQE
LHIESPANKIPNGNIIYSNPAVAEINGVNHLDNIKVSSKDAVEEVYCCKVLKWRPLKSIG
TLAWMITLSDALHNFIDGLAIGASFTLSVLQGLSTSIAILCEEFPHEFGDFAILINAGMS
IPQALTFNFLSACSCYIGLVFGILVGNNFEPSIIFAIAGGMFLYIGLADMFPELNEMLKD
KIKGRRSDLIYFSIQNAGLLSGFAIILLITLYSKEIKLN

>RAT ZIP8
MAPGRAVAGLLLLAATGLGRPSEGPELAFTEDVLRVFGANQSLSAAQLGRLLERLGAAPQ
QGALELGQLHFNQCLSAEDIFSLHGFSNVTQITSSNFTAICPAILQQLNFHPCEDPQKHS
VKPSFSEVWGYGFLSVTIINLASLLGLILTPLIKKSYFPKILTYFVGLAIGTLFSNAIFQ
LIPEAFGFNPKIDNYVEKAVAVFGGFYMLFFVERTLKMLLKTYGQNDHTHFRNDDFGSKE
KAHQPKTLPLPPVNGVTCYANPAVTEPNGHIHFDTVSVVSLQDGKAESSSCTCLKGPKLS
EIGTIAWMITLCDALHNFIDGLAIGASYTLSLLQGLSTSIAILCEEFPHELGDFVILLNA
GMSTRQALLFNFLSACSCYVGLAFGILVGNNFAPNIIFALAGGMFLYISLADMFPEMNDM
LREKVTGRQTDFTFFMIQNAGMLTGFTAILLITLYAGDIELQ

>PIG ZIP8
MAPGRAVAGLLLLAAAGLGGETAGPELAFSEDVLSVFGANRSLSVAQLRRLLEQLGSGPV
EGAPELGQLHFNQCLSAEEIFSLHGFSNATQITSSNFSVICPAVLQQLNFHPCKDRPKHK
TKPSLSEVWGYGFLSVTVINLASLLGLILTPLIKKSYFPKILTYFVGLAIGTLFSNAIFQ
LIPEAFGFNPKVDNYVEKAVAVFGGFYIFFFVERMLKMLLKTYGQNGHTHFGNDDFGPSQ
EKTHQPKTLPAINGVTCYANPAVTEPNGHIHFDNVSVVSLQDGKKESSCCSCLKGPKLSE
IGTIAWMITLSDALHNFIDGLAIGASFTLSLLQGLSTSIAILCEEFPHELGDFVILLNAG
MSTRQALLFNFLSACSCYVGLALGILVGNNFAPNIIFALAGGMFLYISLADMFPEMNDML
REKVTGRKTDFTFFMIQNAGMLTGFIAILLITLYAGEIELE

>HUMAN SLC39A8
MAPGRAVAGLLLLAAAGLGGVAEGPGLAFSEDVLSVFGANLSLSAAQLQHLLEQMGAASR
VGVPEPGQLHFNQCLTAEEIFSLHGFSNATQITSSKFSVICPAVLQQLNFHPCEDRPKHK
TRPSHSEVW GYGFLSVTIINLASLLGLILTPLIKKSYFPKILTFFVGLAIGTLFSNAIFQ
LIPEAFGFDPKVDSYVEKAVAVFGGFYLLFFFERMLK MLLKTYGQNGHTHFGNDNFGPQE
KTHQPKALPAINGVTCYANPAVTEANGHIHFDNVSVVSLQDGKKEPSSCTCLKGPKLSEI
GTIAWMITLCDALHNFIDGLAIGASCTLSLLQGLSTSIAILCEEFPHELGDFVILLN AGM
STRQALLFNFLSACSCYVGLAFGILVGNNFAPNIIFALAGGMFLYISLADMFPEMNDMLR
EKVTGRKTDFTFFMIQNAGMLTGFTAILLITLYAGEIELE

>sp|Q91W10|S39A8_MOUSE Zinc transporter ZIP8 OS=Mus musculus OX=10090 GN=Slc39a8 PE=2 SV=1
>MOUSE
MAPGRAVAGLLLLAATSLGHPSEGPELAFSEDVLSVFGANRSLSAAQLGRLLERLGAASQ
QGALDLGQLHFNQCLSAEDIFSLHGFSNVTQITSSNFSAICPAILQQLNFHPCEDLRKHN
AKPSLSEVWGYGFLSVTIINLASLLGLILTPLIKKSYFPKILTYFVGLAIGTLFSNAIFQ
LIPEAFGFNPKIDNYVEKAVAVFGGFYMLFFVERTLKMLLKTYGQNDHTHFRNDDFGSKE
KTHQPKTLPLPAVNGVTCYANPAVTEPNGHIHFDTVSVVSLQDGKTEPSSCTCLKGPKLS
EIGTIAWMITLCDALHNFIDGLAIGASCTLSLLQGLSTSIAILCEEFPHELGDFVILLNA

GMSTRQALLFNFLSACSCYVGLAFGILVGNNFAPNIIFALAGGMFLYISLADMFPEMNDM
LREKVTGRQTDFTFFMIQNAGMLTGFTAILLITLYAGDIELQ

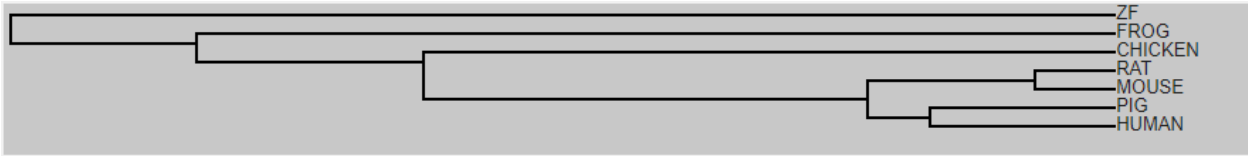

4. Chimera

Gene name: _HHF_SLC39A8-C-terminal, Length: 726 bp, Sequence:
CTGCTGAAGACCTACGGTCAGAACGGCCACACCCACTTCGGCAACGACAACTTTGGTCCGCAGGAGAAGACCCAC
CAACCGAAGGCGCTGCCGGCGATCAACGGTGTGACCTGCTATGCGAACCCGGCGGTTACCGAAGCGAACGGTCA
CATTCACTTCGACAACGTGAGCGTGGTTAGCCTGCAAGATGGCAAGAAAGAGCCGAGCAGCTGCACCTGCCTGAA
GGGTCCGAAACTGAGCGAAATCGGCACCATTGCGTGGATGATCACCCTGTGCGACGCGCTGCACAACTTTATCGA
TGGTCTGGCGATCGGTGCGAGCTGCACCCTGAGCCTGCTGCAGGGCCTGAGCACCAGCATCGCGATTCTGTGCGA
GGAGTTCCCGCACGAGCTGGGTGACTTTGTGATCCTGCTGAACAGCGGTATGAGCGTTGGCCAAGCGGCGTTCTT
TAACCTGCTGAGCGCGATGTGCTGCTACCTGGGTCTGGCGCTGGGCATTCTGCTGGGTAGCAACTTCGCGCCGAA
CGCGATCTTTGCGATTGCGGGTGGCATGTTCCTGTACATCAGCCTGGCGGACATGTTTCCGGAGATGAACAGCATT
ATGGCGAGCCACACCAAAGATTATCAGGAACGTGTGGTTTTCTTTCTGATCCAAAACGCGGGCCTGCTGACCGGTT
TCACCATCATTCTGCTGATCACCCTGTTTGCGGGTGACATCAACCTGCAG

Gene name: _FHH_SLC39A8-c-terminal, Length: 309 bp, Sequence:
GCGGGTATGAGCACCCGTCAGGCGCTGCTGTTCAACTTTCTGAGCGCGTGCAGCTGCTACGTGGGTCTGGCGTTT
GGTATCCTGGTTGGTAACAACTTCGCGCCGAACATCATTTTTGCGCTGGCGGGTGGCATGTTCCTGTATATTAGCC
TGGCGGACATGTTTCCGGAAATGAACGATATGCTGCGTGAGAAGGTGACCGGCCGTAAAACCGACTTCACCTTCT
TTATGATCCAAAACGCGGGCATGCTGACCGGTTTTACCGCGATCCTGCTGATTACCCTGTACGCGGGCGAGATTGA
ACTGGAG

Gene name: NF_Human SLC39A8-middle, Length: 264 bp, Sequence:
GGCTACGGCTTTCTGAGCGTGACCATCATCAACCTGGCCTCCCTGCTGGGCCTGATCCTGACACCACTGATCAAGA
AGTCTTATTTCCCCAAGATCCTGACCTTCTTTGTGGGCCTGGCCATCGGCACACTGTTTAGCAATGCCATCTTCCAG
CTGATCCCCGAGGCCTTTGGCTTCGACCCTAAGGTGGATAGCTACGTGGAGAAGGCCGTGGCCGTGTTTGGCGGC
TTCTATCTGCTGTTCTTTTTCGAGCGGATGCTGAAG

Gene name: NF_HHF SLC39A8-C-terminal, Length: 309 bp, Sequence:
GCAGGAATGTCTACCAGGCAGGCCCTGCTGTTCAACTTTCTGAGCGCCTGCTCCTGTTACGTGGGACTGGCCTTCG
GCATCCTGGTGGGCAACAATTTCGCCCCCAATATCATCTTTGCCCTGGCCGGCGGCATGTTCCTGTATATCAGCCTG
GCCGACATGTTTCCTGAGATGAACGATATGCTGCGGGAGAAGGTGACCGGCAGAAAGACCGACTTCACATTCTTT
ATGATCCAGAATGCCGGCATGCTGACCGGCTTTACAGCCATCCTGCTGATCACACTGTACGCCGGCGAGATCGAG
CTGGAG

Gene name: NH_FHF SLC39A8-middle, Length: 264 bp, Sequence:
GGCTACGGCTTCCTGGCCGTGACCGTGATCAACCTGGCCGCCCTGCTGGGACTGTTCCTGGTGCCCTTCACCAAGA
AGAAGTACTTCCCCAAGGTGCTGATGTACTTCATCGGCCTGGCCATCGGCACCCTGTTCAGCAATGCCGTGCTGCA

GCTGATCCCAGAGGCCCTGGGCCTGGACCCTAAGGACGATAACTACGTGCTGAATGTGGTGGGCATCTTCGGCGG
CTTTTATATCCTGTTTGTGACAGAGAGAATCCTGAAG

Gene name: NH_Fish SLC39A8-middle, Length: 645 bp, Sequence:
GGCTACGGCTTCCTGGCCGTGACCGTGATCAACCTGGCCGCCCTGCTGGGACTGTTCCTGGTGCCCTTCACCAAGA
AGAAGTACTTCCCTAAGGTGCTGATGTACTTCATCGGCCTGGCCATCGGCACCCTGTTTAGCAATGCCGTGCTGCA
GCTGATCCCTGAGGCCCTGGGCCTGGACCCCAAGGACGATAACTACGTGCTGAATGTGGTGGGCATCTTCGGCGG
CTTTTATATCCTGTTCGTGACAGAGAGAATCCTGAAGATGGTGCTGAAGACCGACACAGAGCTGGGCCACAGCCA
CTTTCCCCCTCTGCAGAGCAGCGACGTGACCATCTCTACAATCAGCAACGACGTGGTCATCAGCAATATCTCTGGC
GACATCATCACCAACAATACAAACCACGAGCAGAACAGCATCAGCGAGAAGTCCAACAATCCATCCGAGTCTCCA
GCAGTGGAGCAGAACGCATGCGCACTGTTCGCCTGTAGATGGCTGAAGGGCAGCGCCATGTCCAACATCAAGAC
CGTGGCCTGGATGATCACAGTGTCCGACGCCCTGCACAATTTCATCGATGGCCTGGCCATCGGCGCCTCTTTTACC
CTGTCTCTGCTGAGCGGCTTCAGCACATCCATCGCCATCTTT

5. TM1/4/5 and additional mutants

Gene name : HUMAN_Fish_TM1_SLC39A8_C326F

- Variant name: HUMAN_Fish_TM1_SLC39A8
- Variant name: HUMAN_SLC39A8_C326F

Gene name : _FISH_Human_TM1_SLC39A8_F309C

- Variant name: FISH Human TM1 SLC39A8
- Variant name: Fish SLC39A8 F309C
- Variant name: FISH Human TM1 SLC39A8 F309C_S315Q
- Variant name: FISH Human TM1 SLC39A8 F309C_F317L
- Variant name: FISH Human TM1 SLC39A8 F309C_S315Q_F317L

Gene name: SLC39A8, Sequence:

- Variant name: HUMAN_SLC39A8_C326A
- Variant name: HUMAN_SLC39A8_C326W
- Variant name: HUMAN_SLC39A8_C326Y
- Variant name: HUMAN_SLC39A8_Y131A
- Variant name: HUMAN_SLC39A8_S135A
- Variant name: HUMAN_SLC39A8_Q332S
- Variant name: HUMAN_SLC39A8_Q332A
- Variant name: HUMAN_SLC39A8_L334F

Gene name : HUMAN_Fish_TM1_SLC39A8_C326F, Sequence:
GGTACCACCATGGCCCCTGGCCGGGCCGTGGCCGGCCTGCTGCTGCTGGCTGCCGCCGGACTGGGAGGAGTGGC
AGAGGGCCCAGGCCTGGCCTTTTCCGAGGACGTGCTGAGCGTGTTCGGAGCAAACCTGTCCCTGTCTGCCGCACA
GCTGCAGCACCTGCTGGAGCAGATGGGAGCAGCCAGCCGGGTGGGCGTGCCCGAGCCTGGCCAGCTGCACTTCA
ACCAGTGCCTGACCGCCGAGGAGATCTTTAGCCTGCACGGCTTCTCCAATGCCACCCAGATCACAAGCTCCAAGTT
TAGCGTGATCTGCCCAGCCGTGCTGCAGCAGCTGAACTTCCACCCTTGTGAGGATCGGCCAAAGCACAAGACCAG

ACCTAGCCACTCCGAAGTGTGGGGCTACGGCTTTCTGGCCGTGACAGTGATCAATCTGGCCGCCCTGCTGGGACT
GTTTCTGGTGCCATTCACCAAGAAGTCTTATTTCCCCAAGATCCTGACCTTCTTTGTGGGCCTGGCCATCGGCACAC
TGTTTAGCAACGCCATCTTCCAGCTGATCCCCGAGGCCTTTGGCTTCGACCCTAAGGTGGATTCCTACGTGGAGAA
GGCCGTGGCCGTGTTTGGCGGCTTCTATCTGCTGTTCTTTTTCGAGAGGATGCTGAAGATGCTGCTGAAGACCTAC
GGCCAGAATGGCCACACACACTTTGGCAACGACAATTTCGGCCCCCAGGAGAAGACACACCAGCCAAAGGCCCTG
CCAGCCATCAACGGAGTGACCTGTTATGCCAATCCTGCCGTGACAGAGGCCAACGGCCACATCCACTTTGACAACG
TGAGCGTGGTGTCTCTGCAGGATGGCAAGAAGGAGCCTTCTAGCTGCACCTGTCTGAAGGGCCCAAAGCTGTCCG
AGATCGGCACCATCGCCTGGATGATCACACTGTGCGACGCCCTGCACAACTTTATCGATGGCCTGGCCATCGGCGC
CTCTTTCACCCTGAGCCTGCTGCAGGGCCTGTCTACAAGCATCGCCATCCTGTGCGAGGAGTTTCCCCACGAGCTG
GGCGACTTCGTGATCCTGCTGAACGCCGGCATGTCCACCAGACAGGCCCTGCTGTTTAATTTCCTGAGCGCCTGCT
CCTGTTACGTGGGACTGGCCTTCGGCATCCTGGTGGGCAACAATTTTGCCCCAAATATCATCTTCGCCCTGGCCGG
CGGCATGTTTCTGTATATCAGCCTGGCCGACATGTTCCCCGAGATGAACGATATGCTGAGGGAGAAGGTGACAGG
CCGCAAGACCGACTTCACCTTCTTCATGATCCAGAATGCCGGCATGCTGACCGGCTTCACAGCCATCCTGCTGATCA
CCCTGTACGCCGGCGAGATCGAGCTGGAGTAATCTAGA

Template Name: HUMAN_Fish_TM1_SLC39A8_C326F, Mutagenesis instruction: Variant name: HUMAN_Fish_TM1_SLC39A8, Variant sequence:
GGTACCACCATGGCCCCTGGCCGGGCCGTGGCCGGCCTGCTGCTGCTGGCTGCCGCCGGACTGGGAGGAGTGGC
AGAGGGCCCAGGCCTGGCCTTTTCCGAGGACGTGCTGAGCGTGTTCGGAGCAAACCTGTCCCTGTCTGCCGCACA
GCTGCAGCACCTGCTGGAGCAGATGGGAGCAGCCAGCCGGGTGGGCGTGCCCGAGCCTGGCCAGCTGCACTTCA
ACCAGTGCCTGACCGCCGAGGAGATCTTTAGCCTGCACGGCTTCTCCAATGCCACCCAGATCACAAGCTCCAAGTT
TAGCGTGATCTGCCCAGCCGTGCTGCAGCAGCTGAACTTCCACCCTTGTGAGGATCGGCCAAAGCACAAGACCAG
ACCTAGCCACTCCGAAGTGTGGGGCTACGGCTTTCTGGCCGTGACAGTGATCAATCTGGCCGCCCTGCTGGGACT
GTTTCTGGTGCCATTCACCAAGAAGTCTTATTTCCCCAAGATCCTGACCTTCTTTGTGGGCCTGGCCATCGGCACAC
TGTTTAGCAACGCCATCTTCCAGCTGATCCCCGAGGCCTTTGGCTTCGACCCTAAGGTGGATTCCTACGTGGAGAA
GGCCGTGGCCGTGTTTGGCGGCTTCTATCTGCTGTTCTTTTTCGAGAGGATGCTGAAGATGCTGCTGAAGACCTAC
GGCCAGAATGGCCACACACACTTTGGCAACGACAATTTCGGCCCCCAGGAGAAGACACACCAGCCAAAGGCCCTG
CCAGCCATCAACGGAGTGACCTGTTATGCCAATCCTGCCGTGACAGAGGCCAACGGCCACATCCACTTTGACAACG
TGAGCGTGGTGTCTCTGCAGGATGGCAAGAAGGAGCCTTCTAGCTGCACCTGTCTGAAGGGCCCAAAGCTGTCCG
AGATCGGCACCATCGCCTGGATGATCACACTGTGCGACGCCCTGCACAACTTTATCGATGGCCTGGCCATCGGCGC
CTCTTGCACCCTGAGCCTGCTGCAGGGCCTGTCTACAAGCATCGCCATCCTGTGCGAGGAGTTTCCCCACGAGCTG
GGCGACTTCGTGATCCTGCTGAACGCCGGCATGTCCACCAGACAGGCCCTGCTGTTTAATTTCCTGAGCGCCTGCT
CCTGTTACGTGGGACTGGCCTTCGGCATCCTGGTGGGCAACAATTTTGCCCCAAATATCATCTTCGCCCTGGCCGG
CGGCATGTTTCTGTATATCAGCCTGGCCGACATGTTCCCCGAGATGAACGATATGCTGAGGGAGAAGGTGACAGG
CCGCAAGACCGACTTCACCTTCTTCATGATCCAGAATGCCGGCATGCTGACCGGCTTCACAGCCATCCTGCTGATCA
CCCTGTACGCCGGCGAGATCGAGCTGGAGTAATCTAGA

Template Name: HUMAN_Fish_TM1_SLC39A8_C326F, Mutagenesis instruction: Variant name: HUMAN_SLC39A8_C326F, Variant sequence:
GGTACCACCATGGCCCCTGGCCGGGCCGTGGCCGGCCTGCTGCTGCTGGCTGCCGCCGGACTGGGAGGAGTGGC
AGAGGGCCCAGGCCTGGCCTTTTCCGAGGACGTGCTGAGCGTGTTCGGAGCAAACCTGTCCCTGTCTGCCGCACA
GCTGCAGCACCTGCTGGAGCAGATGGGAGCAGCCAGCCGGGTGGGCGTGCCCGAGCCTGGCCAGCTGCACTTCA
ACCAGTGCCTGACCGCCGAGGAGATCTTTAGCCTGCACGGCTTCTCCAATGCCACCCAGATCACAAGCTCCAAGTT
TAGCGTGATCTGCCCAGCCGTGCTGCAGCAGCTGAACTTCCACCCTTGTGAGGATCGGCCAAAGCACAAGACCAG

ACCTAGCCACTCCGAAGTGTGGGGCTACGGCTTTCTGAGCGTGACAATCATCAATCTGGCCAGCCTGCTGGGACT
GATCCTGACCCCACTGATCAAGAAGTCTTATTTCCCCAAGATCCTGACCTTCTTTGTGGGCCTGGCCATCGGCACAC
TGTTTAGCAACGCCATCTTCCAGCTGATCCCCGAGGCCTTTGGCTTCGACCCTAAGGTGGATTCCTACGTGGAGAA
GGCCGTGGCCGTGTTTGGCGGCTTCTATCTGCTGTTCTTTTTCGAGAGGATGCTGAAGATGCTGCTGAAGACCTAC
GGCCAGAATGGCCACACACACTTTGGCAACGACAATTTCGGCCCCCAGGAGAAGACACACCAGCCAAAGGCCCTG
CCAGCCATCAACGGAGTGACCTGTTATGCCAATCCTGCCGTGACAGAGGCCAACGGCCACATCCACTTTGACAACG
TGAGCGTGGTGTCTCTGCAGGATGGCAAGAAGGAGCCTTCTAGCTGCACCTGTCTGAAGGGCCCAAAGCTGTCCG
AGATCGGCACCATCGCCTGGATGATCACACTGTGCGACGCCCTGCACAACTTTATCGATGGCCTGGCCATCGGCGC
CTCTTTCACCCTGAGCCTGCTGCAGGGCCTGTCTACAAGCATCGCCATCCTGTGCGAGGAGTTTCCCCACGAGCTG
GGCGACTTCGTGATCCTGCTGAACGCCGGCATGTCCACCAGACAGGCCCTGCTGTTTAATTTCCTGAGCGCCTGCT
CCTGTTACGTGGGACTGGCCTTCGGCATCCTGGTGGGCAACAATTTTGCCCCAAATATCATCTTCGCCCTGGCCGG
CGGCATGTTTCTGTATATCAGCCTGGCCGACATGTTCCCCGAGATGAACGATATGCTGAGGGAGAAGGTGACAGG
CCGCAAGACCGACTTCACCTTCTTCATGATCCAGAATGCCGGCATGCTGACCGGCTTCACAGCCATCCTGCTGATCA
CCCTGTACGCCGGCGAGATCGAGCTGGAGTAATCTAGA

Gene name: _FISH_Human_TM1_SLC39A8_F309C, Sequence:
GGTACCACCATGGAGGAGTCCTTCCTGCAGAACGTGCTGGGCTTTTACGGCGAGGAGAGCAGCCTGTCCGTGGGC
AATCTGGAGCTGTTTCTGCAGCTGATCACCTCTCGGAGAGCTGCCGCCGTGGAGGATGAGGGAAATCCCCTGAAG
TCTGCCGAGTGCCTGAGCCTGTCCGAGCTGCTGAGCGCCTTCGGCCTGTCCAACGCCTCTGTGGTGTCTGTGAGCA
ATCTGGAGATGATGTGCCCTGCCATCCTGAACCAGGTGCTGATCCCAGCCTGTCCCTACACCTCTAGCAACCTGAA
TGCCTCCGCCTCTATCAGCGACCACAAAGTGTGGGGCTATGGCTTTCTGAGCGTGACCATCATCAATCTGGCCTCC
CTGCTGGGCCTGATCCTGACACCACTGATCAAGAAGAAGTACTTCCCCAAGGTGCTGATGTACTTCATCGGCCTGG
CCATCGGCACCCTGTTCAGCAACGCCGTGCTGCAGCTGATCCCAGAGGCCCTGGGCCTGGACCCCAAGGACGATA
ACTACGTGCTGAATGTGGTGGGCATCTTCGGCGGCTTTTATATCCTGTTTGTGACAGAGAGAATCCTGAAGATGGT
GCTGAAGACCGACACAGAGCTGGGCCACTCCCACTTCCCCCCTCTGCAGTCCTCTGACGTGACCATCAGCACAATC
TCCAACGACGTGGTCATCAGCAATATCAGCGGCGACATCATCACCAACAATACAAACCACGAGCAGAACAGCATC
AGCGAGAAGAGCAACAATCCTAGCGAGTCCCCAGCAGTGGAGCAGAACGCATGCGCACTGTTCGCATGTAGGTG
GCTGAAGGGCTCCGCCATGTCTAACATCAAGACCGTGGCCTGGATGATCACAGTGAGCGACGCCCTGCACAATTT
CATCGATGGACTGGCAATCGGAGCATCCTGCACCCTGAGCCTGCTGTCCGGCTTTTCTACAAGCATCGCCATCTTCT
GTGAGGAGTTTCCCCACGAGCTGGGCGATTTCGTGATCCTGCTGAACTCTGGCATGAGCGTGGGCCAGGCCGCCT
TCTTTAATCTGCTGTCTGCCATGTGCTGTTATCTGGGACTGGCCCTGGGCATCCTGCTGGGCAGCAACTTCGCCCCT
AATGCCATCTTTGCCATCGCCGGCGGCATGTTCCTGTACATCTCCCTGGCCGATATGTTTCCAGAGATGAACTCCAT
CATGGCCTCTCACACCAAGGACTATCAGGAGCGGGTGGTGTTCTTTCTGATCCAGAATGCCGGCCTGCTGACCGGC
TTCACAATCATCCTGCTGATCACACTGTTTGCCGGCGACATCAACCTGCAGTAATCTAGA

Template Name: _FISH_Human_TM1_SLC39A8_F309C, Mutagenesis instruction: Variant name: FISH Human TM1 SLC39A8, Variant sequence:
GGTACCACCATGGAGGAGTCCTTCCTGCAGAACGTGCTGGGCTTTTACGGCGAGGAGAGCAGCCTGTCCGTGGGC
AATCTGGAGCTGTTTCTGCAGCTGATCACCTCTCGGAGAGCTGCCGCCGTGGAGGATGAGGGAAATCCCCTGAAG
TCTGCCGAGTGCCTGAGCCTGTCCGAGCTGCTGAGCGCCTTCGGCCTGTCCAACGCCTCTGTGGTGTCTGTGAGCA
ATCTGGAGATGATGTGCCCTGCCATCCTGAACCAGGTGCTGATCCCAGCCTGTCCCTACACCTCTAGCAACCTGAA
TGCCTCCGCCTCTATCAGCGACCACAAAGTGTGGGGCTATGGCTTTCTGAGCGTGACCATCATCAATCTGGCCTCC
CTGCTGGGCCTGATCCTGACACCACTGATCAAGAAGAAGTACTTCCCCAAGGTGCTGATGTACTTCATCGGCCTGG

CCATCGGCACCCTGTTCAGCAACGCCGTGCTGCAGCTGATCCCAGAGGCCCTGGGCCTGGACCCCAAGGACGATA
ACTACGTGCTGAATGTGGTGGGCATCTTCGGCGGCTTTTATATCCTGTTTGTGACAGAGAGAATCCTGAAGATGGT
GCTGAAGACCGACACAGAGCTGGGCCACTCCCACTTCCCCCTCTGCAGTCCTCTGACGTGACCATCAGCACAATC
TCCAACGACGTGGTCATCAGCAATATCAGCGGCGACATCATCACCAACAATACAAACCACGAGCAGAACAGCATC
AGCGAGAAGAGCAACAATCCTAGCGAGTCCCCAGCAGTGGAGCAGAACGCATGCGCACTGTTCGCATGTAGGTG
GCTGAAGGGCTCCGCCATGTCTAACATCAAGACCGTGGCCTGGATGATCACAGTGAGCGACGCCCTGCACAATTT
CATCGATGGACTGGCAATCGGAGCATCCTTCACCCTGAGCCTGCTGTCCGGCTTTTCTACAAGCATCGCCATCTTCT
GTGAGGAGTTTCCCCACGAGCTGGGCGATTTCGTGATCCTGCTGAACTCTGGCATGAGCGTGGGCCAGGCCGCCT
TCTTTAATCTGCTGTCTGCCATGTGCTGTTATCTGGGACTGGCCCTGGGCATCCTGCTGGGCAGCAACTTCGCCCCT
AATGCCATCTTTGCCATCGCCGGCGGCATGTTCCTGTACATCTCCCTGGCCGATATGTTTCCAGAGATGAACTCCAT
CATGGCCTCTCACACCAAGGACTATCAGGAGCGGGTGGTGTTCTTTCTGATCCAGAATGCCGGCCTGCTGACCGGC
TTCACAATCATCCTGCTGATCACACTGTTTGCCGGCGACATCAACCTGCAGTAATCTAGA

Template Name: _FISH_Human_TM1_SLC39A8_F309C, Mutagenesis instruction: Variant name: Fish SLC39A8 F309C, Variant sequence:
GGTACCACCATGGAGGAGTCCTTCCTGCAGAACGTGCTGGGCTTTTACGGCGAGGAGAGCAGCCTGTCCGTGGGC
AATCTGGAGCTGTTTCTGCAGCTGATCACCTCTCGGAGAGCTGCCGCCGTGGAGGATGAGGGAAATCCCCTGAAG
TCTGCCGAGTGCCTGAGCCTGTCCGAGCTGCTGAGCGCCTTCGGCCTGTCCAACGCCTCTGTGGTGTCTGTGAGCA
ATCTGGAGATGATGTGCCCTGCCATCCTGAACCAGGTGCTGATCCCAGCCTGTCCCTACACCTCTAGCAACCTGAA
TGCCTCCGCCTCTATCAGCGACCACAAAGTGTGGGGCTATGGCTTTCTGGCCGTGACCGTGATCAATCTGGCCGCC
CTGCTGGGCCTGTTCCTGGTGCCATTCACCAAGAAGAAGTACTTCCCCAAGGTGCTGATGTACTTCATCGGCCTGG
CCATCGGCACCCTGTTCAGCAACGCCGTGCTGCAGCTGATCCCAGAGGCCCTGGGCCTGGACCCCAAGGACGATA
ACTACGTGCTGAATGTGGTGGGCATCTTCGGCGGCTTTTATATCCTGTTTGTGACAGAGAGAATCCTGAAGATGGT
GCTGAAGACCGACACAGAGCTGGGCCACTCCCACTTCCCCCTCTGCAGTCCTCTGACGTGACCATCAGCACAATC
TCCAACGACGTGGTCATCAGCAATATCAGCGGCGACATCATCACCAACAATACAAACCACGAGCAGAACAGCATC
AGCGAGAAGAGCAACAATCCTAGCGAGTCCCCAGCAGTGGAGCAGAACGCATGCGCACTGTTCGCATGTAGGTG
GCTGAAGGGCTCCGCCATGTCTAACATCAAGACCGTGGCCTGGATGATCACAGTGAGCGACGCCCTGCACAATTT
CATCGATGGACTGGCAATCGGAGCATCCTGCACCCTGAGCCTGCTGTCCGGCTTTTCTACAAGCATCGCCATCTTCT
GTGAGGAGTTTCCCCACGAGCTGGGCGATTTCGTGATCCTGCTGAACTCTGGCATGAGCGTGGGCCAGGCCGCCT
TCTTTAATCTGCTGTCTGCCATGTGCTGTTATCTGGGACTGGCCCTGGGCATCCTGCTGGGCAGCAACTTCGCCCCT
AATGCCATCTTTGCCATCGCCGGCGGCATGTTCCTGTACATCTCCCTGGCCGATATGTTTCCAGAGATGAACTCCAT
CATGGCCTCTCACACCAAGGACTATCAGGAGCGGGTGGTGTTCTTTCTGATCCAGAATGCCGGCCTGCTGACCGGC
TTCACAATCATCCTGCTGATCACACTGTTTGCCGGCGACATCAACCTGCAGTAATCTAGA

Template name: _FISH_Human_TM1_SLC39A8_F309C, Mutagenesis instruction: Variant name: FISH Human TM1 SLC39A8 F309C_S315Q, Variant sequence:
GGTACCACCATGGAGGAGTCCTTCCTGCAGAACGTGCTGGGCTTTTACGGCGAGGAGAGCAGCCTGTCCGTGGGC
AATCTGGAGCTGTTTCTGCAGCTGATCACCTCTCGGAGAGCTGCCGCCGTGGAGGATGAGGGAAATCCCCTGAAG
TCTGCCGAGTGCCTGAGCCTGTCCGAGCTGCTGAGCGCCTTCGGCCTGTCCAACGCCTCTGTGGTGTCTGTGAGCA
ATCTGGAGATGATGTGCCCTGCCATCCTGAACCAGGTGCTGATCCCAGCCTGTCCCTACACCTCTAGCAACCTGAA
TGCCTCCGCCTCTATCAGCGACCACAAAGTGTGGGGCTATGGCTTTCTGAGCGTGACCATCATCAATCTGGCCTCC
CTGCTGGGCCTGATCCTGACACCACTGATCAAGAAGAAGTACTTCCCCAAGGTGCTGATGTACTTCATCGGCCTGG
CCATCGGCACCCTGTTCAGCAACGCCGTGCTGCAGCTGATCCCAGAGGCCCTGGGCCTGGACCCCAAGGACGATA
ACTACGTGCTGAATGTGGTGGGCATCTTCGGCGGCTTTTATATCCTGTTTGTGACAGAGAGAATCCTGAAGATGGT

GCTGAAGACCGACACAGAGCTGGGCCACTCCCACTTCCCCCCTCTGCAGTCCTCTGACGTGACCATCAGCACAATC
TCCAACGACGTGGTCATCAGCAATATCAGCGGCGACATCATCACCAACAATACAAACCACGAGCAGAACAGCATC
AGCGAGAAGAGCAACAATCCTAGCGAGTCCCCAGCAGTGGAGCAGAACGCATGCGCACTGTTCGCATGTAGGTG
GCTGAAGGGCTCCGCCATGTCTAACATCAAGACCGTGGCCTGGATGATCACAGTGAGCGACGCCCTGCACAATTT
CATCGATGGACTGGCAATCGGAGCATCCTGCACCCTGAGCCTGCTGCAGGGCTTTTCTACAAGCATCGCCATCTTC
TGTGAGGAGTTTCCCCACGAGCTGGGCGATTTCGTGATCCTGCTGAACTCTGGCATGAGCGTGGGCCAGGCCGCC
TTCTTTAATCTGCTGTCTGCCATGTGCTGTTATCTGGGACTGGCCCTGGGCATCCTGCTGGGCAGCAACTTCGCCCC
TAATGCCATCTTTGCCATCGCCGGCGGCATGTTCCTGTACATCTCCCTGGCCGATATGTTTCCAGAGATGAACTCCA
TCATGGCCTCTCACACCAAGGACTATCAGGAGCGGGTGGTGTTCTTTCTGATCCAGAATGCCGGCCTGCTGACCGG
CTTCACAATCATCCTGCTGATCACACTGTTTGCCGGCGACATCAACCTGCAGTAATCTAGA

Template name: _FISH_Human_TM1_SLC39A8_F309C, Mutagenesis instruction: Variant name: FISH Human TM1 SLC39A8 F309C_F317L, Variant sequence:
GGTACCACCATGGAGGAGTCCTTCCTGCAGAACGTGCTGGGCTTTTACGGCGAGGAGAGCAGCCTGTCCGTGGGC
AATCTGGAGCTGTTTCTGCAGCTGATCACCTCTCGGAGAGCTGCCGCCGTGGAGGATGAGGGAAATCCCCTGAAG
TCTGCCGAGTGCCTGAGCCTGTCCGAGCTGCTGAGCGCCTTCGGCCTGTCCAACGCCTCTGTGGTGTCTGTGAGCA
ATCTGGAGATGATGTGCCCTGCCATCCTGAACCAGGTGCTGATCCCAGCCTGTCCCTACACCTCTAGCAACCTGAA
TGCCTCCGCCTCTATCAGCGACCACAAAGTGTGGGGCTATGGCTTTCTGAGCGTGACCATCATCAATCTGGCCTCC
CTGCTGGGCCTGATCCTGACACCACTGATCAAGAAGAAGTACTTCCCCAAGGTGCTGATGTACTTCATCGGCCTGG
CCATCGGCACCCTGTTCAGCAACGCCGTGCTGCAGCTGATCCCAGAGGCCCTGGGCCTGGACCCCAAGGACGATA
ACTACGTGCTGAATGTGGTGGGCATCTTCGGCGGCTTTTATATCCTGTTTGTGACAGAGAGAATCCTGAAGATGGT
GCTGAAGACCGACACAGAGCTGGGCCACTCCCACTTCCCCCCTCTGCAGTCCTCTGACGTGACCATCAGCACAATC
TCCAACGACGTGGTCATCAGCAATATCAGCGGCGACATCATCACCAACAATACAAACCACGAGCAGAACAGCATC
AGCGAGAAGAGCAACAATCCTAGCGAGTCCCCAGCAGTGGAGCAGAACGCATGCGCACTGTTCGCATGTAGGTG
GCTGAAGGGCTCCGCCATGTCTAACATCAAGACCGTGGCCTGGATGATCACAGTGAGCGACGCCCTGCACAATTT
CATCGATGGACTGGCAATCGGAGCATCCTGCACCCTGAGCCTGCTGTCCGGCCTGTCTACAAGCATCGCCATCTTC
TGTGAGGAGTTTCCCCACGAGCTGGGCGATTTCGTGATCCTGCTGAACTCTGGCATGAGCGTGGGCCAGGCCGCC
TTCTTTAATCTGCTGTCTGCCATGTGCTGTTATCTGGGACTGGCCCTGGGCATCCTGCTGGGCAGCAACTTCGCCCC
TAATGCCATCTTTGCCATCGCCGGCGGCATGTTCCTGTACATCTCCCTGGCCGATATGTTTCCAGAGATGAACTCCA
TCATGGCCTCTCACACCAAGGACTATCAGGAGCGGGTGGTGTTCTTTCTGATCCAGAATGCCGGCCTGCTGACCGG
CTTCACAATCATCCTGCTGATCACACTGTTTGCCGGCGACATCAACCTGCAGTAATCTAGA

Template name: _FISH_Human_TM1_SLC39A8_F309C, Mutagenesis instruction: Variant name: FISH Human TM1 SLC39A8 F309C_S315Q_F317L, Variant sequence:
GGTACCACCATGGAGGAGTCCTTCCTGCAGAACGTGCTGGGCTTTTACGGCGAGGAGAGCAGCCTGTCCGTGGGC
AATCTGGAGCTGTTTCTGCAGCTGATCACCTCTCGGAGAGCTGCCGCCGTGGAGGATGAGGGAAATCCCCTGAAG
TCTGCCGAGTGCCTGAGCCTGTCCGAGCTGCTGAGCGCCTTCGGCCTGTCCAACGCCTCTGTGGTGTCTGTGAGCA
ATCTGGAGATGATGTGCCCTGCCATCCTGAACCAGGTGCTGATCCCAGCCTGTCCCTACACCTCTAGCAACCTGAA
TGCCTCCGCCTCTATCAGCGACCACAAAGTGTGGGGCTATGGCTTTCTGAGCGTGACCATCATCAATCTGGCCTCC
CTGCTGGGCCTGATCCTGACACCACTGATCAAGAAGAAGTACTTCCCCAAGGTGCTGATGTACTTCATCGGCCTGG
CCATCGGCACCCTGTTCAGCAACGCCGTGCTGCAGCTGATCCCAGAGGCCCTGGGCCTGGACCCCAAGGACGATA
ACTACGTGCTGAATGTGGTGGGCATCTTCGGCGGCTTTTATATCCTGTTTGTGACAGAGAGAATCCTGAAGATGGT
GCTGAAGACCGACACAGAGCTGGGCCACTCCCACTTCCCCCCTCTGCAGTCCTCTGACGTGACCATCAGCACAATC
TCCAACGACGTGGTCATCAGCAATATCAGCGGCGACATCATCACCAACAATACAAACCACGAGCAGAACAGCATC

AGCGAGAAGAGCAACAATCCTAGCGAGTCCCCAGCAGTGGAGCAGAACGCATGCGCACTGTTCGCATGTAGGTG
GCTGAAGGGCTCCGCCATGTCTAACATCAAGACCGTGGCCTGGATGATCACAGTGAGCGACGCCCTGCACAATTT
CATCGATGGACTGGCAATCGGAGCATCCTGCACCCTGAGCCTGCTGCAGGGCCTGTCTACAAGCATCGCCATCTTC
TGTGAGGAGTTTCCCCACGAGCTGGGCGATTTCGTGATCCTGCTGAACTCTGGCATGAGCGTGGGCCAGGCCGCC
TTCTTTAATCTGCTGTCTGCCATGTGCTGTTATCTGGGACTGGCCCTGGGCATCCTGCTGGGCAGCAACTTCGCCCC
TAATGCCATCTTTGCCATCGCCGGCGGCATGTTCCTGTACATCTCCCTGGCCGATATGTTTCCAGAGATGAACTCCA
TCATGGCCTCTCACACCAAGGACTATCAGGAGCGGGTGGTGTTCTTTCTGATCCAGAATGCCGGCCTGCTGACCGG
CTTCACAATCATCCTGCTGATCACACTGTTTGCCGGCGACATCAACCTGCAGTAATCTAGA

Gene name: SLC39A8, Sequence:
GGTACCACCATGGCCCCGGGTCGCGCGGTGGCCGGGCTCCTGTTGCTGGCGGCCGCCGGCCTCGGAGGAGTGGC
GGAGGGGCCAGGGCTAGCCTTCAGCGAGGATGTGCTGAGCGTGTTCGGCGCGAATCTGAGCCTGTCGGCGGCGC
AGCTCCAGCACTTGCTGGAGCAGATGGGAGCCGCCTCCCGCGTGGGCGTCCCGGAGCCTGGCCAGCTGCACTTCA
ACCAGTGTTTAACTGCTGAAGAGATCTTTTCCCTTCATGGCTTTTCAAATGCTACCCAAATAACCAGCTCCAAATTCT
CTGTCATCTGTCCAGCAGTCTTACAGCAATTGAACTTTCACCCATGTGAGGATCGGCCCAAGCACAAAACAAGACC
AAGTCATTCAGAAGTTTGGGGATATGGATTCCTGTCAGTGACGATTATTAATCTGGCATCTCTCCTCGGATTGATTT
TGACTCCACTGATAAAGAAATCTTATTTCCCAAAGATTTTGACCTTTTTTGTGGGGCTGGCTATTGGGACTCTTTTTT
CAAATGCAATTTTCCAACTTATTCCAGAGGCATTTGGATTTGATCCCAAAGTCGACAGTTATGTTGAGAAGGCAGT
TGCTGTGTTTGGTGGATTTTACCTACTTTTCTTTTTTGAAAGAATGCTAAAGATGTTATTAAAGACATATGGTCAGA
ATGGTCATACCCACTTTGGAAATGATAACTTTGGTCCTCAAGAAAAAACTCATCAACCTAAAGCATTACCTGCCATC
AATGGTGTGACATGCTATGCAAATCCTGCTGTCACAGAAGCTAATGGACATATCCATTTTGATAATGTCAGTGTGG
TATCTCTACAGGATGGAAAAAAAGAGCCAAGTTCATGTACCTGTTTGAAGGGGCCCAAACTGTCAGAAATAGGGA
CGATTGCCTGGATGATAACGCTCTGCGATGCCCTCCACAATTTCATCGATGGCCTGGCGATTGGGGCTTCCTGCAC
CTTGTCTCTCCTTCAGGGACTCAGTACTTCCATAGCAATCCTATGTGAGGAGTTTCCCCACGAGTTAGGAGACTTTG
TGATCCTACTCAATGCAGGGATGAGCACTCGACAAGCCTTGCTATTCAACTTCCTTTCTGCATGTTCCTGCTATGTT
GGGCTAGCTTTTGGCATTTTGGTGGGCAACAATTTCGCTCCAAATATTATATTTGCACTTGCTGGAGGCATGTTCCT
CTATATTTCTCTGGCAGATATGTTTCCAGAGATGAATGATATGCTGAGAGAAAAGGTAACTGGAAGAAAAACCGA
TTTCACCTTCTTCATGATTCAGAATGCTGGAATGTTAACTGGATTCACAGCCATTCTACTCATTACCTTGTATGCAGG
AGAAATCGAATTGGAGTAATCTAGA

Template name: SLC39A8, Mutagenesis instruction: Variant name: HUMAN_SLC39A8_C326A, Variant sequence:
GGTACCACCATGGCCCCGGGTCGCGCGGTGGCCGGGCTCCTGTTGCTGGCGGCCGCCGGCCTCGGAGGAGTGGC
GGAGGGGCCAGGGCTAGCCTTCAGCGAGGATGTGCTGAGCGTGTTCGGCGCGAATCTGAGCCTGTCGGCGGCGC
AGCTCCAGCACTTGCTGGAGCAGATGGGAGCCGCCTCCCGCGTGGGCGTCCCGGAGCCTGGCCAGCTGCACTTCA
ACCAGTGTTTAACTGCTGAAGAGATCTTTTCCCTTCATGGCTTTTCAAATGCTACCCAAATAACCAGCTCCAAATTCT
CTGTCATCTGTCCAGCAGTCTTACAGCAATTGAACTTTCACCCATGTGAGGATCGGCCCAAGCACAAAACAAGACC
AAGTCATTCAGAAGTTTGGGGATATGGATTCCTGTCAGTGACGATTATTAATCTGGCATCTCTCCTCGGATTGATTT
TGACTCCACTGATAAAGAAATCTTATTTCCCAAAGATTTTGACCTTTTTTGTGGGGCTGGCTATTGGGACTCTTTTTT
CAAATGCAATTTTCCAACTTATTCCAGAGGCATTTGGATTTGATCCCAAAGTCGACAGTTATGTTGAGAAGGCAGT
TGCTGTGTTTGGTGGATTTTACCTACTTTTCTTTTTTGAAAGAATGCTAAAGATGTTATTAAAGACATATGGTCAGA
ATGGTCATACCCACTTTGGAAATGATAACTTTGGTCCTCAAGAAAAAACTCATCAACCTAAAGCATTACCTGCCATC
AATGGTGTGACATGCTATGCAAATCCTGCTGTCACAGAAGCTAATGGACATATCCATTTTGATAATGTCAGTGTGG

TATCTCTACAGGATGGAAAAAAAGAGCCAAGTTCATGTACCTGTTTGAAGGGGCCCAAACTGTCAGAAATAGGGA
CGATTGCCTGGATGATAACGCTCTGCGATGCCCTCCACAATTTCATCGATGGCCTGGCGATTGGGGCTTCCGCCAC
CTTGTCTCTCCTTCAGGGACTCAGTACTTCCATAGCAATCCTATGTGAGGAGTTTCCCCACGAGTTAGGAGACTTTG
TGATCCTACTCAATGCAGGGATGAGCACTCGACAAGCCTTGCTATTCAACTTCCTTTCTGCATGTTCCTGCTATGTT
GGGCTAGCTTTTGGCATTTTGGTGGGCAACAATTTCGCTCCAAATATTATATTTGCACTTGCTGGAGGCATGTTCCT
CTATATTTCTCTGGCAGATATGTTTCCAGAGATGAATGATATGCTGAGAGAAAAGGTAACTGGAAGAAAAACCGA
TTTCACCTTCTTCATGATTCAGAATGCTGGAATGTTAACTGGATTCACAGCCATTCTACTCATTACCTTGTATGCAGG
AGAAATCGAATTGGAGTAATCTAGA

Template name: SLC39A8, Mutagenesis instruction: Variant name: HUMAN_SLC39A8_C326W, Variant sequence:
GGTACCACCATGGCCCCGGGTCGCGCGGTGGCCGGGCTCCTGTTGCTGGCGGCCGCCGGCCTCGGAGGAGTGGC
GGAGGGGCCAGGGCTAGCCTTCAGCGAGGATGTGCTGAGCGTGTTCGGCGCGAATCTGAGCCTGTCGGCGGCGC
AGCTCCAGCACTTGCTGGAGCAGATGGGAGCCGCCTCCCGCGTGGGCGTCCCGGAGCCTGGCCAGCTGCACTTCA
ACCAGTGTTTAACTGCTGAAGAGATCTTTTCCCTTCATGGCTTTTCAAATGCTACCCAAATAACCAGCTCCAAATTCT
CTGTCATCTGTCCAGCAGTCTTACAGCAATTGAACTTTCACCCATGTGAGGATCGGCCCAAGCACAAAACAAGACC
AAGTCATTCAGAAGTTTGGGGATATGGATTCCTGTCAGTGACGATTATTAATCTGGCATCTCTCCTCGGATTGATTT
TGACTCCACTGATAAAGAAATCTTATTTCCCAAAGATTTTGACCTTTTTTGTGGGGCTGGCTATTGGGACTCTTTTTT
CAAATGCAATTTTCCAACTTATTCCAGAGGCATTTGGATTTGATCCCAAAGTCGACAGTTATGTTGAGAAGGCAGT
TGCTGTGTTTGGTGGATTTTACCTACTTTTCTTTTTTGAAAGAATGCTAAAGATGTTATTAAAGACATATGGTCAGA
ATGGTCATACCCACTTTGGAAATGATAACTTTGGTCCTCAAGAAAAAACTCATCAACCTAAAGCATTACCTGCCATC
AATGGTGTGACATGCTATGCAAATCCTGCTGTCACAGAAGCTAATGGACATATCCATTTTGATAATGTCAGTGTGG
TATCTCTACAGGATGGAAAAAAAGAGCCAAGTTCATGTACCTGTTTGAAGGGGCCCAAACTGTCAGAAATAGGGA
CGATTGCCTGGATGATAACGCTCTGCGATGCCCTCCACAATTTCATCGATGGCCTGGCGATTGGGGCTTCCTGGAC
CTTGTCTCTCCTTCAGGGACTCAGTACTTCCATAGCAATCCTATGTGAGGAGTTTCCCCACGAGTTAGGAGACTTTG
TGATCCTACTCAATGCAGGGATGAGCACTCGACAAGCCTTGCTATTCAACTTCCTTTCTGCATGTTCCTGCTATGTT
GGGCTAGCTTTTGGCATTTTGGTGGGCAACAATTTCGCTCCAAATATTATATTTGCACTTGCTGGAGGCATGTTCCT
CTATATTTCTCTGGCAGATATGTTTCCAGAGATGAATGATATGCTGAGAGAAAAGGTAACTGGAAGAAAAACCGA
TTTCACCTTCTTCATGATTCAGAATGCTGGAATGTTAACTGGATTCACAGCCATTCTACTCATTACCTTGTATGCAGG
AGAAATCGAATTGGAGTAATCTAGA

Template name: SLC39A8, Mutagenesis instruction: Variant name: HUMAN_SLC39A8_C326Y, Variant sequence:
GGTACCACCATGGCCCCGGGTCGCGCGGTGGCCGGGCTCCTGTTGCTGGCGGCCGCCGGCCTCGGAGGAGTGGC
GGAGGGGCCAGGGCTAGCCTTCAGCGAGGATGTGCTGAGCGTGTTCGGCGCGAATCTGAGCCTGTCGGCGGCGC
AGCTCCAGCACTTGCTGGAGCAGATGGGAGCCGCCTCCCGCGTGGGCGTCCCGGAGCCTGGCCAGCTGCACTTCA
ACCAGTGTTTAACTGCTGAAGAGATCTTTTCCCTTCATGGCTTTTCAAATGCTACCCAAATAACCAGCTCCAAATTCT
CTGTCATCTGTCCAGCAGTCTTACAGCAATTGAACTTTCACCCATGTGAGGATCGGCCCAAGCACAAAACAAGACC
AAGTCATTCAGAAGTTTGGGGATATGGATTCCTGTCAGTGACGATTATTAATCTGGCATCTCTCCTCGGATTGATTT
TGACTCCACTGATAAAGAAATCTTATTTCCCAAAGATTTTGACCTTTTTTGTGGGGCTGGCTATTGGGACTCTTTTTT
CAAATGCAATTTTCCAACTTATTCCAGAGGCATTTGGATTTGATCCCAAAGTCGACAGTTATGTTGAGAAGGCAGT
TGCTGTGTTTGGTGGATTTTACCTACTTTTCTTTTTTGAAAGAATGCTAAAGATGTTATTAAAGACATATGGTCAGA
ATGGTCATACCCACTTTGGAAATGATAACTTTGGTCCTCAAGAAAAAACTCATCAACCTAAAGCATTACCTGCCATC
AATGGTGTGACATGCTATGCAAATCCTGCTGTCACAGAAGCTAATGGACATATCCATTTTGATAATGTCAGTGTGG

TATCTCTACAGGATGGAAAAAAAGAGCCAAGTTCATGTACCTGTTTGAAGGGGCCCAAACTGTCAGAAATAGGGA
CGATTGCCTGGATGATAACGCTCTGCGATGCCCTCCACAATTTCATCGATGGCCTGGCGATTGGGGCTTCCTACAC
CTTGTCTCTCCTTCAGGGACTCAGTACTTCCATAGCAATCCTATGTGAGGAGTTTCCCCACGAGTTAGGAGACTTTG
TGATCCTACTCAATGCAGGGATGAGCACTCGACAAGCCTTGCTATTCAACTTCCTTTCTGCATGTTCCTGCTATGTT
GGGCTAGCTTTTGGCATTTTGGTGGGCAACAATTTCGCTCCAAATATTATATTTGCACTTGCTGGAGGCATGTTCCT
CTATATTTCTCTGGCAGATATGTTTCCAGAGATGAATGATATGCTGAGAGAAAAGGTAACTGGAAGAAAAACCGA
TTTCACCTTCTTCATGATTCAGAATGCTGGAATGTTAACTGGATTCACAGCCATTCTACTCATTACCTTGTATGCAGG
AGAAATCGAATTGGAGTAATCTAGA

Template name: SLC39A8, Mutagenesis instruction: Variant name: HUMAN_SLC39A8_Y131A, Variant sequence:
GGTACCACCATGGCCCCGGGTCGCGCGGTGGCCGGGCTCCTGTTGCTGGCGGCCGCCGGCCTCGGAGGAGTGGC
GGAGGGGCCAGGGCTAGCCTTCAGCGAGGATGTGCTGAGCGTGTTCGGCGCGAATCTGAGCCTGTCGGCGGCGC
AGCTCCAGCACTTGCTGGAGCAGATGGGAGCCGCCTCCCGCGTGGGCGTCCCGGAGCCTGGCCAGCTGCACTTCA
ACCAGTGTTTAACTGCTGAAGAGATCTTTTCCCTTCATGGCTTTTCAAATGCTACCCAAATAACCAGCTCCAAATTCT
CTGTCATCTGTCCAGCAGTCTTACAGCAATTGAACTTTCACCCATGTGAGGATCGGCCCAAGCACAAAACAAGACC
AAGTCATTCAGAAGTTTGGGGAGCCGGATTCCTGTCAGTGACGATTATTAATCTGGCATCTCTCCTCGGATTGATTT
TGACTCCACTGATAAAGAAATCTTATTTCCCAAAGATTTTGACCTTTTTTGTGGGGCTGGCTATTGGGACTCTTTTTT
CAAATGCAATTTTCCAACTTATTCCAGAGGCATTTGGATTTGATCCCAAAGTCGACAGTTATGTTGAGAAGGCAGT
TGCTGTGTTTGGTGGATTTTACCTACTTTTCTTTTTTGAAAGAATGCTAAAGATGTTATTAAAGACATATGGTCAGA
ATGGTCATACCCACTTTGGAAATGATAACTTTGGTCCTCAAGAAAAAACTCATCAACCTAAAGCATTACCTGCCATC
AATGGTGTGACATGCTATGCAAATCCTGCTGTCACAGAAGCTAATGGACATATCCATTTTGATAATGTCAGTGTGG
TATCTCTACAGGATGGAAAAAAAGAGCCAAGTTCATGTACCTGTTTGAAGGGGCCCAAACTGTCAGAAATAGGGA
CGATTGCCTGGATGATAACGCTCTGCGATGCCCTCCACAATTTCATCGATGGCCTGGCGATTGGGGCTTCCTGCAC
CTTGTCTCTCCTTCAGGGACTCAGTACTTCCATAGCAATCCTATGTGAGGAGTTTCCCCACGAGTTAGGAGACTTTG
TGATCCTACTCAATGCAGGGATGAGCACTCGACAAGCCTTGCTATTCAACTTCCTTTCTGCATGTTCCTGCTATGTT
GGGCTAGCTTTTGGCATTTTGGTGGGCAACAATTTCGCTCCAAATATTATATTTGCACTTGCTGGAGGCATGTTCCT
CTATATTTCTCTGGCAGATATGTTTCCAGAGATGAATGATATGCTGAGAGAAAAGGTAACTGGAAGAAAAACCGA
TTTCACCTTCTTCATGATTCAGAATGCTGGAATGTTAACTGGATTCACAGCCATTCTACTCATTACCTTGTATGCAGG
AGAAATCGAATTGGAGTAATCTAGA

Template name: SLC39A8, Mutagenesis instruction: Variant name: HUMAN_SLC39A8_S135A, Variant sequence:
GGTACCACCATGGCCCCGGGTCGCGCGGTGGCCGGGCTCCTGTTGCTGGCGGCCGCCGGCCTCGGAGGAGTGGC
GGAGGGGCCAGGGCTAGCCTTCAGCGAGGATGTGCTGAGCGTGTTCGGCGCGAATCTGAGCCTGTCGGCGGCGC
AGCTCCAGCACTTGCTGGAGCAGATGGGAGCCGCCTCCCGCGTGGGCGTCCCGGAGCCTGGCCAGCTGCACTTCA
ACCAGTGTTTAACTGCTGAAGAGATCTTTTCCCTTCATGGCTTTTCAAATGCTACCCAAATAACCAGCTCCAAATTCT
CTGTCATCTGTCCAGCAGTCTTACAGCAATTGAACTTTCACCCATGTGAGGATCGGCCCAAGCACAAAACAAGACC
AAGTCATTCAGAAGTTTGGGGATATGGATTCCTGGCCGTGACGATTATTAATCTGGCATCTCTCCTCGGATTGATTT
TGACTCCACTGATAAAGAAATCTTATTTCCCAAAGATTTTGACCTTTTTTGTGGGGCTGGCTATTGGGACTCTTTTTT
CAAATGCAATTTTCCAACTTATTCCAGAGGCATTTGGATTTGATCCCAAAGTCGACAGTTATGTTGAGAAGGCAGT
TGCTGTGTTTGGTGGATTTTACCTACTTTTCTTTTTTGAAAGAATGCTAAAGATGTTATTAAAGACATATGGTCAGA
ATGGTCATACCCACTTTGGAAATGATAACTTTGGTCCTCAAGAAAAAACTCATCAACCTAAAGCATTACCTGCCATC
AATGGTGTGACATGCTATGCAAATCCTGCTGTCACAGAAGCTAATGGACATATCCATTTTGATAATGTCAGTGTGG

TATCTCTACAGGATGGAAAAAAAGAGCCAAGTTCATGTACCTGTTTGAAGGGGCCCAAACTGTCAGAAATAGGGA
CGATTGCCTGGATGATAACGCTCTGCGATGCCCTCCACAATTTCATCGATGGCCTGGCGATTGGGGCTTCCTGCAC
CTTGTCTCTCCTTCAGGGACTCAGTACTTCCATAGCAATCCTATGTGAGGAGTTTCCCCACGAGTTAGGAGACTTTG
TGATCCTACTCAATGCAGGGATGAGCACTCGACAAGCCTTGCTATTCAACTTCCTTTCTGCATGTTCCTGCTATGTT
GGGCTAGCTTTTGGCATTTTGGTGGGCAACAATTTCGCTCCAAATATTATATTTGCACTTGCTGGAGGCATGTTCCT
CTATATTTCTCTGGCAGATATGTTTCCAGAGATGAATGATATGCTGAGAGAAAAGGTAACTGGAAGAAAAACCGA
TTTCACCTTCTTCATGATTCAGAATGCTGGAATGTTAACTGGATTCACAGCCATTCTACTCATTACCTTGTATGCAGG
AGAAATCGAATTGGAGTAATCTAGA

Template name: SLC39A8, Mutagenesis instruction: Variant name: HUMAN_SLC39A8_Q332S, Variant sequence:
GGTACCACCATGGCCCCGGGTCGCGCGGTGGCCGGGCTCCTGTTGCTGGCGGCCGCCGGCCTCGGAGGAGTGGC
GGAGGGGCCAGGGCTAGCCTTCAGCGAGGATGTGCTGAGCGTGTTCGGCGCGAATCTGAGCCTGTCGGCGGCGC
AGCTCCAGCACTTGCTGGAGCAGATGGGAGCCGCCTCCCGCGTGGGCGTCCCGGAGCCTGGCCAGCTGCACTTCA
ACCAGTGTTTAACTGCTGAAGAGATCTTTTCCCTTCATGGCTTTTCAAATGCTACCCAAATAACCAGCTCCAAATTCT
CTGTCATCTGTCCAGCAGTCTTACAGCAATTGAACTTTCACCCATGTGAGGATCGGCCCAAGCACAAAACAAGACC
AAGTCATTCAGAAGTTTGGGGATATGGATTCCTGTCAGTGACGATTATTAATCTGGCATCTCTCCTCGGATTGATTT
TGACTCCACTGATAAAGAAATCTTATTTCCCAAAGATTTTGACCTTTTTTGTGGGGCTGGCTATTGGGACTCTTTTTT
CAAATGCAATTTTCCAACTTATTCCAGAGGCATTTGGATTTGATCCCAAAGTCGACAGTTATGTTGAGAAGGCAGT
TGCTGTGTTTGGTGGATTTTACCTACTTTTCTTTTTTGAAAGAATGCTAAAGATGTTATTAAAGACATATGGTCAGA
ATGGTCATACCCACTTTGGAAATGATAACTTTGGTCCTCAAGAAAAAACTCATCAACCTAAAGCATTACCTGCCATC
AATGGTGTGACATGCTATGCAAATCCTGCTGTCACAGAAGCTAATGGACATATCCATTTTGATAATGTCAGTGTGG
TATCTCTACAGGATGGAAAAAAAGAGCCAAGTTCATGTACCTGTTTGAAGGGGCCCAAACTGTCAGAAATAGGGA
CGATTGCCTGGATGATAACGCTCTGCGATGCCCTCCACAATTTCATCGATGGCCTGGCGATTGGGGCTTCCTGCAC
CTTGTCTCTCCTTAGCGGACTCAGTACTTCCATAGCAATCCTATGTGAGGAGTTTCCCCACGAGTTAGGAGACTTTG
TGATCCTACTCAATGCAGGGATGAGCACTCGACAAGCCTTGCTATTCAACTTCCTTTCTGCATGTTCCTGCTATGTT
GGGCTAGCTTTTGGCATTTTGGTGGGCAACAATTTCGCTCCAAATATTATATTTGCACTTGCTGGAGGCATGTTCCT
CTATATTTCTCTGGCAGATATGTTTCCAGAGATGAATGATATGCTGAGAGAAAAGGTAACTGGAAGAAAAACCGA
TTTCACCTTCTTCATGATTCAGAATGCTGGAATGTTAACTGGATTCACAGCCATTCTACTCATTACCTTGTATGCAGG
AGAAATCGAATTGGAGTAATCTAGA

Template name: SLC39A8, Mutagenesis instruction: Variant name: HUMAN_SLC39A8_Q332A, Variant sequence:
GGTACCACCATGGCCCCGGGTCGCGCGGTGGCCGGGCTCCTGTTGCTGGCGGCCGCCGGCCTCGGAGGAGTGGC
GGAGGGGCCAGGGCTAGCCTTCAGCGAGGATGTGCTGAGCGTGTTCGGCGCGAATCTGAGCCTGTCGGCGGCGC
AGCTCCAGCACTTGCTGGAGCAGATGGGAGCCGCCTCCCGCGTGGGCGTCCCGGAGCCTGGCCAGCTGCACTTCA
ACCAGTGTTTAACTGCTGAAGAGATCTTTTCCCTTCATGGCTTTTCAAATGCTACCCAAATAACCAGCTCCAAATTCT
CTGTCATCTGTCCAGCAGTCTTACAGCAATTGAACTTTCACCCATGTGAGGATCGGCCCAAGCACAAAACAAGACC
AAGTCATTCAGAAGTTTGGGGATATGGATTCCTGTCAGTGACGATTATTAATCTGGCATCTCTCCTCGGATTGATTT
TGACTCCACTGATAAAGAAATCTTATTTCCCAAAGATTTTGACCTTTTTTGTGGGGCTGGCTATTGGGACTCTTTTTT
CAAATGCAATTTTCCAACTTATTCCAGAGGCATTTGGATTTGATCCCAAAGTCGACAGTTATGTTGAGAAGGCAGT
TGCTGTGTTTGGTGGATTTTACCTACTTTTCTTTTTTGAAAGAATGCTAAAGATGTTATTAAAGACATATGGTCAGA
ATGGTCATACCCACTTTGGAAATGATAACTTTGGTCCTCAAGAAAAAACTCATCAACCTAAAGCATTACCTGCCATC
AATGGTGTGACATGCTATGCAAATCCTGCTGTCACAGAAGCTAATGGACATATCCATTTTGATAATGTCAGTGTGG

TATCTCTACAGGATGGAAAAAAAGAGCCAAGTTCATGTACCTGTTTGAAGGGGCCCAAACTGTCAGAAATAGGGA
CGATTGCCTGGATGATAACGCTCTGCGATGCCCTCCACAATTTCATCGATGGCCTGGCGATTGGGGCTTCCTGCAC
CTTGTCTCTCCTTGCCGGACTCAGTACTTCCATAGCAATCCTATGTGAGGAGTTTCCCCACGAGTTAGGAGACTTTG
TGATCCTACTCAATGCAGGGATGAGCACTCGACAAGCCTTGCTATTCAACTTCCTTTCTGCATGTTCCTGCTATGTT
GGGCTAGCTTTTGGCATTTTGGTGGGCAACAATTTCGCTCCAAATATTATATTTGCACTTGCTGGAGGCATGTTCCT
CTATATTTCTCTGGCAGATATGTTTCCAGAGATGAATGATATGCTGAGAGAAAAGGTAACTGGAAGAAAAACCGA
TTTCACCTTCTTCATGATTCAGAATGCTGGAATGTTAACTGGATTCACAGCCATTCTACTCATTACCTTGTATGCAGG
AGAAATCGAATTGGAGTAATCTAGA

Template name: SLC39A8, Mutagenesis instruction: Variant name: HUMAN_SLC39A8_L334F, Variant sequence:
GGTACCACCATGGCCCCGGGTCGCGCGGTGGCCGGGCTCCTGTTGCTGGCGGCCGCCGGCCTCGGAGGAGTGGC
GGAGGGGCCAGGGCTAGCCTTCAGCGAGGATGTGCTGAGCGTGTTCGGCGCGAATCTGAGCCTGTCGGCGGCGC
AGCTCCAGCACTTGCTGGAGCAGATGGGAGCCGCCTCCCGCGTGGGCGTCCCGGAGCCTGGCCAGCTGCACTTCA
ACCAGTGTTTAACTGCTGAAGAGATCTTTTCCCTTCATGGCTTTTCAAATGCTACCCAAATAACCAGCTCCAAATTCT
CTGTCATCTGTCCAGCAGTCTTACAGCAATTGAACTTTCACCCATGTGAGGATCGGCCCAAGCACAAAACAAGACC
AAGTCATTCAGAAGTTTGGGGATATGGATTCCTGTCAGTGACGATTATTAATCTGGCATCTCTCCTCGGATTGATTT
TGACTCCACTGATAAAGAAATCTTATTTCCCAAAGATTTTGACCTTTTTTGTGGGGCTGGCTATTGGGACTCTTTTTT
CAAATGCAATTTTCCAACTTATTCCAGAGGCATTTGGATTTGATCCCAAAGTCGACAGTTATGTTGAGAAGGCAGT
TGCTGTGTTTGGTGGATTTTACCTACTTTTCTTTTTTGAAAGAATGCTAAAGATGTTATTAAAGACATATGGTCAGA
ATGGTCATACCCACTTTGGAAATGATAACTTTGGTCCTCAAGAAAAAACTCATCAACCTAAAGCATTACCTGCCATC
AATGGTGTGACATGCTATGCAAATCCTGCTGTCACAGAAGCTAATGGACATATCCATTTTGATAATGTCAGTGTGG
TATCTCTACAGGATGGAAAAAAAGAGCCAAGTTCATGTACCTGTTTGAAGGGGCCCAAACTGTCAGAAATAGGGA
CGATTGCCTGGATGATAACGCTCTGCGATGCCCTCCACAATTTCATCGATGGCCTGGCGATTGGGGCTTCCTGCAC
CTTGTCTCTCCTTCAGGGATTCAGTACTTCCATAGCAATCCTATGTGAGGAGTTTCCCCACGAGTTAGGAGACTTTG
TGATCCTACTCAATGCAGGGATGAGCACTCGACAAGCCTTGCTATTCAACTTCCTTTCTGCATGTTCCTGCTATGTT
GGGCTAGCTTTTGGCATTTTGGTGGGCAACAATTTCGCTCCAAATATTATATTTGCACTTGCTGGAGGCATGTTCCT
CTATATTTCTCTGGCAGATATGTTTCCAGAGATGAATGATATGCTGAGAGAAAAGGTAACTGGAAGAAAAACCGA
TTTCACCTTCTTCATGATTCAGAATGCTGGAATGTTAACTGGATTCACAGCCATTCTACTCATTACCTTGTATGCAGG
AGAAATCGAATTGGAGTAATCTAGA

6. CDG Mutants

FLAG tag inserted after a putative signal peptide (aa 1-22) in the N-terminal region:
> hSLC39A8 Signal NFLAG_pcDNA3.1(+)
> hSLC39A8 A391T Signal NFLAG:
>hSLC39A8_V33M_pcDNA3.1+_Signal NFLAG pcDNA3.1(+)
>hSLC39A8_G38R_pcDNA3.1+_Signal NFLAG pcDNA3.1(+)
>hSLC39A8_C113S_pcDNA3.1+_Signal NFLAG pcDNA3.1(+)
>hSLC39A8_G204C_pcDNA3.1+_Signal NFLAG pcDNA3.1(+)
>hSLC39A8_S335T_pcDNA3.1+_Signal NFLAG pcDNA3.1(+)
>hSLC39A8_I340N_pcDNA3.1+_Signal NFLAG pcDNA3.1(+)

> **hSLC39A8 Signal NFLAG_pcDNA3.1(+)**
GGTACCACCATGGCCCCGGGTCGCGCGGTGGCCGGGCTCCTGTTGCTGGCGGCCGCCGGCCTCGGAGGAGTGGC
GGATTACAAGGATGACGACGATAAGGAGGGGCCAGGGCTAGCCTTCAGCGAGGATGTGCTGAGCGTGTTCGGC
GCGAATCTGAGCCTGTCGGCGGCGCAGCTCCAGCACTTGCTGGAGCAGATGGGAGCCGCCTCCCGCGTGGGCGT
CCCGGAGCCTGGCCAGCTGCACTTCAACCAGTGTTTAACTGCTGAAGAGATCTTTTCCCTTCATGGCTTTTCAAATG
CTACCCAAATAACCAGCTCCAAATTCTCTGTCATCTGTCCAGCAGTCTTACAGCAATTGAACTTTCACCCATGTGAG
GATCGGCCCAAGCACAAAACAAGACCAAGTCATTCAGAAGTTTGGGGATATGGATTCCTGTCAGTGACGATTATT
AATCTGGCATCTCTCCTCGGATTGATTTTGACTCCACTGATAAAGAAATCTTATTTCCCAAAGATTTTGACCTTTTTT
GTGGGGCTGGCTATTGGGACTCTTTTTTCAAATGCAATTTTCCAACTTATTCCAGAGGCATTTGGATTTGATCCCAA
AGTCGACAGTTATGTTGAGAAGGCAGTTGCTGTGTTTGGTGGATTTTACCTACTTTTCTTTTTTGAAAGAATGCTAA
AGATGTTATTAAAGACATATGGTCAGAATGGTCATACCCACTTTGGAAATGATAACTTTGGTCCTCAAGAAAAAAC
TCATCAACCTAAAGCATTACCTGCCATCAATGGTGTGACATGCTATGCAAATCCTGCTGTCACAGAAGCTAATGGA
CATATCCATTTTGATAATGTCAGTGTGGTATCTCTACAGGATGGAAAAAAAGAGCCAAGTTCATGTACCTGTTTGA
AGGGGCCCAAACTGTCAGAAATAGGGACGATTGCCTGGATGATAACGCTCTGCGATGCCCTCCACAATTTCATCG
ATGGCCTGGCGATTGGGGCTTCCTGCACCTTGTCTCTCCTTCAGGGACTCAGTACTTCCATAGCAATCCTATGTGAG
GAGTTTCCCCACGAGTTAGGAGACTTTGTGATCCTACTCAATGCAGGGATGAGCACTCGACAAGCCTTGCTATTCA
ACTTCCTTTCTGCATGTTCCTGCTATGTTGGGCTAGCTTTTGGCATTTTGGTGGGCAACAATTTCGCTCCAAATATTA
TATTTGCACTTGCTGGAGGCATGTTCCTCTATATTTCTCTGGCAGATATGTTTCCAGAGATGAATGATATGCTGAGA
GAAAAGGTAACTGGAAGAAAAACCGATTTCACCTTCTTCATGATTCAGAATGCTGGAATGTTAACTGGATTCACAG
CCATTCTACTCATTACCTTGTATGCAGGAGAAATCGAATTGGAGTAATCTAGA

> **hSLC39A8 A391T Signal NFLAG:**
GGTACCACCATGGCCCCGGGTCGCGCGGTGGCCGGGCTCCTGTTGCTGGCGGCCGCCGGCCTCGGAGGAGTGGC
GGATTACAAGGATGACGACGATAAGGAGGGGCCAGGGCTAGCCTTCAGCGAGGATGTGCTGAGCGTGTTCGGC
GCGAATCTGAGCCTGTCGGCGGCGCAGCTCCAGCACTTGCTGGAGCAGATGGGAGCCGCCTCCCGCGTGGGCGT
CCCGGAGCCTGGCCAGCTGCACTTCAACCAGTGTTTAACTGCTGAAGAGATCTTTTCCCTTCATGGCTTTTCAAATG
CTACCCAAATAACCAGCTCCAAATTCTCTGTCATCTGTCCAGCAGTCTTACAGCAATTGAACTTTCACCCATGTGAG
GATCGGCCCAAGCACAAAACAAGACCAAGTCATTCAGAAGTTTGGGGATATGGATTCCTGTCAGTGACGATTATT
AATCTGGCATCTCTCCTCGGATTGATTTTGACTCCACTGATAAAGAAATCTTATTTCCCAAAGATTTTGACCTTTTTT
GTGGGGCTGGCTATTGGGACTCTTTTTTCAAATGCAATTTTCCAACTTATTCCAGAGGCATTTGGATTTGATCCCAA
AGTCGACAGTTATGTTGAGAAGGCAGTTGCTGTGTTTGGTGGATTTTACCTACTTTTCTTTTTTGAAAGAATGCTAA
AGATGTTATTAAAGACATATGGTCAGAATGGTCATACCCACTTTGGAAATGATAACTTTGGTCCTCAAGAAAAAAC
TCATCAACCTAAAGCATTACCTGCCATCAATGGTGTGACATGCTATGCAAATCCTGCTGTCACAGAAGCTAATGGA
CATATCCATTTTGATAATGTCAGTGTGGTATCTCTACAGGATGGAAAAAAAGAGCCAAGTTCATGTACCTGTTTGA
AGGGGCCCAAACTGTCAGAAATAGGGACGATTGCCTGGATGATAACGCTCTGCGATGCCCTCCACAATTTCATCG
ATGGCCTGGCGATTGGGGCTTCCTGCACCTTGTCTCTCCTTCAGGGACTCAGTACTTCCATAGCAATCCTATGTGAG
GAGTTTCCCCACGAGTTAGGAGACTTTGTGATCCTACTCAATGCAGGGATGAGCACTCGACAAGCCTTGCTATTCA
ACTTCCTTTCTGCATGTTCCTGCTATGTTGGGCTAGCTTTTGGCATTTTGGTGGGCAACAATTTCACCCCAAATATTA
TATTTGCACTTGCTGGAGGCATGTTCCTCTATATTTCTCTGGCAGATATGTTTCCAGAGATGAATGATATGCTGAGA
GAAAAGGTAACTGGAAGAAAAACCGATTTCACCTTCTTCATGATTCAGAATGCTGGAATGTTAACTGGATTCACAG
CCATTCTACTCATTACCTTGTATGCAGGAGAAATCGAATTGGAGTAATCTAGA

>hSLC39A8_V33M_pcDNA3.1+_Signal NFLAG pcDNA3.1(+)
GGTACCACCATGGCCCCGGGTCGCGCGGTGGCCGGGCTCCTGTTGCTGGCGGCCGCCGGCCTCGGAGGAGTGGC
GgattacaaggatgacgacgataagGAGGGGCCAGGGCTAGCCTTCAGCGAGGATATGCTGAGCGTGTTCGGCGCGAAT
CTGAGCCTGTCGGCGGCGCAGCTCCAGCACTTGCTGGAGCAGATGGGAGCCGCCTCCCGCGTGGGCGTCCCGGA
GCCTGGCCAGCTGCACTTCAACCAGTGTTTAACTGCTGAAGAGATCTTTTCCCTTCATGGCTTTTCAAATGCTACCC
AAATAACCAGCTCCAAATTCTCTGTCATCTGTCCAGCAGTCTTACAGCAATTGAACTTTCACCCATGTGAGGATCGG

CCCAAGCACAAAACAAGACCAAGTCATTCAGAAGTTTGGGGATATGGATTCCTGTCAGTGACGATTATTAATCTGG
CATCTCTCCTCGGATTGATTTTGACTCCACTGATAAAGAAATCTTATTTCCCAAAGATTTTGACCTTTTTTGTGGGGC
TGGCTATTGGGACTCTTTTTTCAAATGCAATTTTCCAACTTATTCCAGAGGCATTTGGATTTGATCCCAAAGTCGAC
AGTTATGTTGAGAAGGCAGTTGCTGTGTTTGGTGGATTTTACCTACTTTTCTTTTTTGAAAGAATGCTAAAGATGTT
ATTAAAGACATATGGTCAGAATGGTCATACCCACTTTGGAAATGATAACTTTGGTCCTCAAGAAAAAACTCATCAA
CCTAAAGCATTACCTGCCATCAATGGTGTGACATGCTATGCAAATCCTGCTGTCACAGAAGCTAATGGACATATCC
ATTTTGATAATGTCAGTGTGGTATCTCTACAGGATGGAAAAAAAGAGCCAAGTTCATGTACCTGTTTGAAGGGGCC
CAAACTGTCAGAAATAGGGACGATTGCCTGGATGATAACGCTCTGCGATGCCCTCCACAATTTCATCGATGGCCTG
GCGATTGGGGCTTCCTGCACCTTGTCTCTCCTTCAGGGACTCAGTACTTCCATAGCAATCCTATGTGAGGAGTTTCC
CCACGAGTTAGGAGACTTTGTGATCCTACTCAATGCAGGGATGAGCACTCGACAAGCCTTGCTATTCAACTTCCTTT
CTGCATGTTCCTGCTATGTTGGGCTAGCTTTTGGCATTTTGGTGGGCAACAATTTCGCTCCAAATATTATATTTGCA
CTTGCTGGAGGCATGTTCCTCTATATTTCTCTGGCAGATATGTTTCCAGAGATGAATGATATGCTGAGAGAAAAGG
TAACTGGAAGAAAAACCGATTTCACCTTCTTCATGATTCAGAATGCTGGAATGTTAACTGGATTCACAGCCATTCTA
CTCATTACCTTGTATGCAGGAGAAATCGAATTGGAGTAATCTAGA

>hSLC39A8_G38R_pcDNA3.1+_Signal NFLAG pcDNA3.1(+)
GGTACCACCATGGCCCCGGGTCGCGCGGTGGCCGGGCTCCTGTTGCTGGCGGCCGCCGGCCTCGGAGGAGTGGC
GgattacaaggatgacgacgataagGAGGGGCCAGGGCTAGCCTTCAGCGAGGATGTGCTGAGCGTGTTCCGGGCGAAT
CTGAGCCTGTCGGCGGCGCAGCTCCAGCACTTGCTGGAGCAGATGGGAGCCGCCTCCCGCGTGGGCGTCCCGGA
GCCTGGCCAGCTGCACTTCAACCAGTGTTTAACTGCTGAAGAGATCTTTTCCCTTCATGGCTTTTCAAATGCTACCC
AAATAACCAGCTCCAAATTCTCTGTCATCTGTCCAGCAGTCTTACAGCAATTGAACTTTCACCCATGTGAGGATCGG
CCCAAGCACAAAACAAGACCAAGTCATTCAGAAGTTTGGGGATATGGATTCCTGTCAGTGACGATTATTAATCTGG
CATCTCTCCTCGGATTGATTTTGACTCCACTGATAAAGAAATCTTATTTCCCAAAGATTTTGACCTTTTTTGTGGGGC
TGGCTATTGGGACTCTTTTTTCAAATGCAATTTTCCAACTTATTCCAGAGGCATTTGGATTTGATCCCAAAGTCGAC
AGTTATGTTGAGAAGGCAGTTGCTGTGTTTGGTGGATTTTACCTACTTTTCTTTTTTGAAAGAATGCTAAAGATGTT
ATTAAAGACATATGGTCAGAATGGTCATACCCACTTTGGAAATGATAACTTTGGTCCTCAAGAAAAAACTCATCAA
CCTAAAGCATTACCTGCCATCAATGGTGTGACATGCTATGCAAATCCTGCTGTCACAGAAGCTAATGGACATATCC
ATTTTGATAATGTCAGTGTGGTATCTCTACAGGATGGAAAAAAAGAGCCAAGTTCATGTACCTGTTTGAAGGGGCC
CAAACTGTCAGAAATAGGGACGATTGCCTGGATGATAACGCTCTGCGATGCCCTCCACAATTTCATCGATGGCCTG
GCGATTGGGGCTTCCTGCACCTTGTCTCTCCTTCAGGGACTCAGTACTTCCATAGCAATCCTATGTGAGGAGTTTCC
CCACGAGTTAGGAGACTTTGTGATCCTACTCAATGCAGGGATGAGCACTCGACAAGCCTTGCTATTCAACTTCCTTT
CTGCATGTTCCTGCTATGTTGGGCTAGCTTTTGGCATTTTGGTGGGCAACAATTTCGCTCCAAATATTATATTTGCA
CTTGCTGGAGGCATGTTCCTCTATATTTCTCTGGCAGATATGTTTCCAGAGATGAATGATATGCTGAGAGAAAAGG
TAACTGGAAGAAAAACCGATTTCACCTTCTTCATGATTCAGAATGCTGGAATGTTAACTGGATTCACAGCCATTCTA
CTCATTACCTTGTATGCAGGAGAAATCGAATTGGAGTAATCTAGA

>hSLC39A8_C113S_pcDNA3.1+_Signal NFLAG pcDNA3.1(+)
GGTACCACCATGGCCCCGGGTCGCGCGGTGGCCGGGCTCCTGTTGCTGGCGGCCGCCGGCCTCGGAGGAGTGGC
GgattacaaggatgacgacgataagGAGGGGCCAGGGCTAGCCTTCAGCGAGGATGTGCTGAGCGTGTTCGGCGCGAAT
CTGAGCCTGTCGGCGGCGCAGCTCCAGCACTTGCTGGAGCAGATGGGAGCCGCCTCCCGCGTGGGCGTCCCGGA
GCCTGGCCAGCTGCACTTCAACCAGTGTTTAACTGCTGAAGAGATCTTTTCCCTTCATGGCTTTTCAAATGCTACCC
AAATAACCAGCTCCAAATTCTCTGTCATCTGTCCAGCAGTCTTACAGCAATTGAACTTTCACCCAAGCGAGGATCGG
CCCAAGCACAAAACAAGACCAAGTCATTCAGAAGTTTGGGGATATGGATTCCTGTCAGTGACGATTATTAATCTGG
CATCTCTCCTCGGATTGATTTTGACTCCACTGATAAAGAAATCTTATTTCCCAAAGATTTTGACCTTTTTTGTGGGGC
TGGCTATTGGGACTCTTTTTTCAAATGCAATTTTCCAACTTATTCCAGAGGCATTTGGATTTGATCCCAAAGTCGAC
AGTTATGTTGAGAAGGCAGTTGCTGTGTTTGGTGGATTTTACCTACTTTTCTTTTTTGAAAGAATGCTAAAGATGTT
ATTAAAGACATATGGTCAGAATGGTCATACCCACTTTGGAAATGATAACTTTGGTCCTCAAGAAAAAACTCATCAA
CCTAAAGCATTACCTGCCATCAATGGTGTGACATGCTATGCAAATCCTGCTGTCACAGAAGCTAATGGACATATCC

ATTTTGATAATGTCAGTGTGGTATCTCTACAGGATGGAAAAAAAGAGCCAAGTTCATGTACCTGTTTGAAGGGGCC
CAAACTGTCAGAAATAGGGACGATTGCCTGGATGATAACGCTCTGCGATGCCCTCCACAATTTCATCGATGGCCTG
GCGATTGGGGCTTCCTGCACCTTGTCTCTCCTTCAGGGACTCAGTACTTCCATAGCAATCCTATGTGAGGAGTTTCC
CCACGAGTTAGGAGACTTTGTGATCCTACTCAATGCAGGGATGAGCACTCGACAAGCCTTGCTATTCAACTTCCTTT
CTGCATGTTCCTGCTATGTTGGGCTAGCTTTTGGCATTTTGGTGGGCAACAATTTCGCTCCAAATATTATATTTGCA
CTTGCTGGAGGCATGTTCCTCTATATTTCTCTGGCAGATATGTTTCCAGAGATGAATGATATGCTGAGAGAAAAGG
TAACTGGAAGAAAAACCGATTTCACCTTCTTCATGATTCAGAATGCTGGAATGTTAACTGGATTCACAGCCATTCTA
CTCATTACCTTGTATGCAGGAGAAATCGAATTGGAGTAATCTAGA

>hSLC39A8_G204C_pcDNA3.1+_Signal NFLAG pcDNA3.1(+)
GGTACCACCATGGCCCCGGGTCGCGCGGTGGCCGGGCTCCTGTTGCTGGCGGCCGCCGGCCTCGGAGGAGTGGC
GgattacaaggatgacgacgataagGAGGGGCCAGGGCTAGCCTTCAGCGAGGATGTGCTGAGCGTGTTCGGCGCGAAT
CTGAGCCTGTCGGCGGCGCAGCTCCAGCACTTGCTGGAGCAGATGGGAGCCGCCTCCCGCGTGGGCGTCCCGGA
GCCTGGCCAGCTGCACTTCAACCAGTGTTTAACTGCTGAAGAGATCTTTTCCCTTCATGGCTTTTCAAATGCTACCC
AAATAACCAGCTCCAAATTCTCTGTCATCTGTCCAGCAGTCTTACAGCAATTGAACTTTCACCCATGTGAGGATCGG
CCCAAGCACAAAACAAGACCAAGTCATTCAGAAGTTTGGGGATATGGATTCCTGTCAGTGACGATTATTAATCTGG
CATCTCTCCTCGGATTGATTTTGACTCCACTGATAAAGAAATCTTATTTCCCAAAGATTTTGACCTTTTTTGTGGGGC
TGGCTATTGGGACTCTTTTTTCAAATGCAATTTTCCAACTTATTCCAGAGGCATTTGGATTTGATCCCAAAGTCGAC
AGTTATGTTGAGAAGGCAGTTGCTGTGTTTTGCGGATTTTACCTACTTTTCTTTTTTGAAAGAATGCTAAAGATGTT
ATTAAAGACATATGGTCAGAATGGTCATACCCACTTTGGAAATGATAACTTTGGTCCTCAAGAAAAAACTCATCAA
CCTAAAGCATTACCTGCCATCAATGGTGTGACATGCTATGCAAATCCTGCTGTCACAGAAGCTAATGGACATATCC
ATTTTGATAATGTCAGTGTGGTATCTCTACAGGATGGAAAAAAAGAGCCAAGTTCATGTACCTGTTTGAAGGGGCC
CAAACTGTCAGAAATAGGGACGATTGCCTGGATGATAACGCTCTGCGATGCCCTCCACAATTTCATCGATGGCCTG
GCGATTGGGGCTTCCTGCACCTTGTCTCTCCTTCAGGGACTCAGTACTTCCATAGCAATCCTATGTGAGGAGTTTCC
CCACGAGTTAGGAGACTTTGTGATCCTACTCAATGCAGGGATGAGCACTCGACAAGCCTTGCTATTCAACTTCCTTT
CTGCATGTTCCTGCTATGTTGGGCTAGCTTTTGGCATTTTGGTGGGCAACAATTTCGCTCCAAATATTATATTTGCA
CTTGCTGGAGGCATGTTCCTCTATATTTCTCTGGCAGATATGTTTCCAGAGATGAATGATATGCTGAGAGAAAAGG
TAACTGGAAGAAAAACCGATTTCACCTTCTTCATGATTCAGAATGCTGGAATGTTAACTGGATTCACAGCCATTCTA
CTCATTACCTTGTATGCAGGAGAAATCGAATTGGAGTAATCTAGA

>hSLC39A8_S335T_pcDNA3.1+_Signal NFLAG pcDNA3.1(+)
GGTACCACCATGGCCCCGGGTCGCGCGGTGGCCGGGCTCCTGTTGCTGGCGGCCGCCGGCCTCGGAGGAGTGGC
GgattacaaggatgacgacgataagGAGGGGCCAGGGCTAGCCTTCAGCGAGGATGTGCTGAGCGTGTTCGGCGCGAAT
CTGAGCCTGTCGGCGGCGCAGCTCCAGCACTTGCTGGAGCAGATGGGAGCCGCCTCCCGCGTGGGCGTCCCGGA
GCCTGGCCAGCTGCACTTCAACCAGTGTTTAACTGCTGAAGAGATCTTTTCCCTTCATGGCTTTTCAAATGCTACCC
AAATAACCAGCTCCAAATTCTCTGTCATCTGTCCAGCAGTCTTACAGCAATTGAACTTTCACCCATGTGAGGATCGG
CCCAAGCACAAAACAAGACCAAGTCATTCAGAAGTTTGGGGATATGGATTCCTGTCAGTGACGATTATTAATCTGG
CATCTCTCCTCGGATTGATTTTGACTCCACTGATAAAGAAATCTTATTTCCCAAAGATTTTGACCTTTTTTGTGGGGC
TGGCTATTGGGACTCTTTTTTCAAATGCAATTTTCCAACTTATTCCAGAGGCATTTGGATTTGATCCCAAAGTCGAC
AGTTATGTTGAGAAGGCAGTTGCTGTGTTTGGTGGATTTTACCTACTTTTCTTTTTTGAAAGAATGCTAAAGATGTT
ATTAAAGACATATGGTCAGAATGGTCATACCCACTTTGGAAATGATAACTTTGGTCCTCAAGAAAAAACTCATCAA
CCTAAAGCATTACCTGCCATCAATGGTGTGACATGCTATGCAAATCCTGCTGTCACAGAAGCTAATGGACATATCC
ATTTTGATAATGTCAGTGTGGTATCTCTACAGGATGGAAAAAAAGAGCCAAGTTCATGTACCTGTTTGAAGGGGCC
CAAACTGTCAGAAATAGGGACGATTGCCTGGATGATAACGCTCTGCGATGCCCTCCACAATTTCATCGATGGCCTG
GCGATTGGGGCTTCCTGCACCTTGTCTCTCCTTCAGGGACTCACCACTTCCATAGCAATCCTATGTGAGGAGTTTCC
CCACGAGTTAGGAGACTTTGTGATCCTACTCAATGCAGGGATGAGCACTCGACAAGCCTTGCTATTCAACTTCCTTT
CTGCATGTTCCTGCTATGTTGGGCTAGCTTTTGGCATTTTGGTGGGCAACAATTTCGCTCCAAATATTATATTTGCA
CTTGCTGGAGGCATGTTCCTCTATATTTCTCTGGCAGATATGTTTCCAGAGATGAATGATATGCTGAGAGAAAAGG

TAACTGGAAGAAAAACCGATTTCACCTTCTTCATGATTCAGAATGCTGGAATGTTAACTGGATTCACAGCCATTCTA
CTCATTACCTTGTATGCAGGAGAAATCGAATTGGAGTAATCTAGA

>hSLC39A8_I340N_pcDNA3.1+_Signal NFLAG pcDNA3.1(+)
GGTACCACCATGGCCCCGGGTCGCGCGGTGGCCGGGCTCCTGTTGCTGGCGGCCGCCGGCCTCGGAGGAGTGGC
GgattacaaggatgacgacgataagGAGGGGCCAGGGCTAGCCTTCAGCGAGGATGTGCTGAGCGTGTTCGGCGCGAAT
CTGAGCCTGTCGGCGGCGCAGCTCCAGCACTTGCTGGAGCAGATGGGAGCCGCCTCCCGCGTGGGCGTCCCGGA
GCCTGGCCAGCTGCACTTCAACCAGTGTTTAACTGCTGAAGAGATCTTTTCCCTTCATGGCTTTTCAAATGCTACCC
AAATAACCAGCTCCAAATTCTCTGTCATCTGTCCAGCAGTCTTACAGCAATTGAACTTTCACCCATGTGAGGATCGG
CCCAAGCACAAAACAAGACCAAGTCATTCAGAAGTTTGGGGATATGGATTCCTGTCAGTGACGATTATTAATCTGG
CATCTCTCCTCGGATTGATTTTGACTCCACTGATAAAGAAATCTTATTTCCCAAAGATTTTGACCTTTTTTGTGGGGC
TGGCTATTGGGACTCTTTTTTCAAATGCAATTTTCCAACTTATTCCAGAGGCATTTGGATTTGATCCCAAAGTCGAC
AGTTATGTTGAGAAGGCAGTTGCTGTGTTTGGTGGATTTTACCTACTTTTCTTTTTTGAAAGAATGCTAAAGATGTT
ATTAAAGACATATGGTCAGAATGGTCATACCCACTTTGGAAATGATAACTTTGGTCCTCAAGAAAAAACTCATCAA
CCTAAAGCATTACCTGCCATCAATGGTGTGACATGCTATGCAAATCCTGCTGTCACAGAAGCTAATGGACATATCC
ATTTTGATAATGTCAGTGTGGTATCTCTACAGGATGGAAAAAAAGAGCCAAGTTCATGTACCTGTTTGAAGGGGCC
CAAACTGTCAGAAATAGGGACGATTGCCTGGATGATAACGCTCTGCGATGCCCTCCACAATTTCATCGATGGCCTG
GCGATTGGGGCTTCCTGCACCTTGTCTCTCCTTCAGGGACTCAGTACTTCCATAGCAAACCTATGTGAGGAGTTTCC
CCACGAGTTAGGAGACTTTGTGATCCTACTCAATGCAGGGATGAGCACTCGACAAGCCTTGCTATTCAACTTCCTTT
CTGCATGTTCCTGCTATGTTGGGCTAGCTTTTGGCATTTTGGTGGGCAACAATTTCGCTCCAAATATTATATTTGCA
CTTGCTGGAGGCATGTTCCTCTATATTTCTCTGGCAGATATGTTTCCAGAGATGAATGATATGCTGAGAGAAAAGG
TAACTGGAAGAAAAACCGATTTCACCTTCTTCATGATTCAGAATGCTGGAATGTTAACTGGATTCACAGCCATTCTA
CTCATTACCTTGTATGCAGGAGAAATCGAATTGGAGTAATCTAGA

Supplementary Information: Cell Culture Reagents

Critical reagents

Reagents

Reagent	Description	Catalog number
L-WRN Conditioned media	Conditioned media to enrich for stem cell growth and expansion	Analytical Biological Services C01-0770
Advanced DMEM/F12	Primary culture media	Gibco 12634
Pen/Strep	Primary culture media	Gibco 15140122
L-Glut	Primary culture media	Gibco 25030081
FBS	Primary culture media	Sigma F6178-500ml
Y-27632	Rho kinase inhibitor; used to improve cell viability after passage	Tocris 1254
DAPT	Gamma secretase inhibitor; for differentiation media	Millipore 565784
TPP 24-well plates	Cell culture plates	Sigma Z707791
Matrigel	Extracellular matrix	Corning 354234

PBS		Hyclone SH300028.02
0.5M EDTA	For passaging	Invitrogen 15575-020
70µm cell strainers	For passaging	Corning 08-771-2 (352350)
10X Trypsin solution	For passaging	Sigma T4549
DMEM/F12	Wash media for passaging	Sigma D6421-500ml
PC Transwell inserts	0.4 µm polycarbonate membrane; for submerged culture	Corning 3413
40µm cell strainers	For seeding TWs	Corning 05-771-1 (352340)
Attachment factor	For seeding TWs	Thermo S006100
Manganese		Sigma M4880
White clear bottom 96well plates	Assay plate for Apotox-Glo	Corning 3903
Qiazol	Lysis buffer for RNA extraction	Qiagen 79306
Direct-zol RNA mini prep kit	RNA extraction	Zymo research R2052
High-Capacity cDNA Reverse Transcription Kit	cDNA synthesis kit	Thermo 4374966
TaqMan universal PCR master mix	2X qPCR reaction mix for TLDA	Applied Biosystems 4304437
DMSO	Compound control	Sigma D2650
Collagenase type I	Establishment of spheroids	Invitrogen 17100-017
Gentamycin	Establishment of spheroids	Sigma G1397

Compounds

JNJ	BID	Notes
16535207	9351027, 48780907	Efavirenz R (inactive)
26892567	47806864, 43128694	Efavirenz S (active)
74701315	50444093	Efavirenz series analog, Cmpd 9

Media recipes

Primary culture media

Reagent	Volume
Advanced DMEM/F12	500 ml
100X L-Glutamine	6.25 ml
100X Pen/Strep	6.25 ml
FBS (heat inactivated)	125 ml

Washing media

Reagent	Volume
Advanced DMEM/F12 with HEPES	500 ml
100X L-Glutamine	5 ml
100X Pen/Strep	5 ml
FBS (heat inactivated)	50 ml

Collagenase solution

Reagent	Volume
Collagenase type I	20 mg
Gentamycin	10 ul
Washing Media	10 ml

PBS-EDTA solution 0.5 mM – for passaging

Reagent	Volume
PBS	500 ml
0.5M EDTA	500 µl

1X Trypsin-EDTA solution

Reagent	Volume
0.5 mM PBS-EDTA solution	9 ml
10X Trypsin	1 ml

50% L-WRN conditioned medium

Reagent	Volume
Primary Culture Media	40 ml
100% L-WRN media	40 ml

DAPT+LPS differentiation media

Reagent	Volume
Primary culture media	5 ml
25 mM DAPT	2 µl (10 µM final)
1 mg/ml LPS	5 µl (1 µg/ml final)
